# Identification of Mendel's White Flower Character

**DOI:** 10.1371/journal.pone.0013230

**Published:** 2010-10-11

**Authors:** Roger P. Hellens, Carol Moreau, Kui Lin-Wang, Kathy E. Schwinn, Susan J. Thomson, Mark W. E. J. Fiers, Tonya J. Frew, Sarah R. Murray, Julie M. I. Hofer, Jeanne M. E. Jacobs, Kevin M. Davies, Andrew C. Allan, Abdelhafid Bendahmane, Clarice J. Coyne, Gail M. Timmerman-Vaughan, T. H. Noel Ellis

**Affiliations:** 1 The New Zealand Institute for Plant and Food Research Ltd, Auckland, New Zealand; 2 Department of Crop Genetics, John Innes Centre, Norwich, United Kingdom; 3 The New Zealand Institute for Plant and Food Research Ltd, Palmerston North, New Zealand; 4 The New Zealand Institute for Plant and Food Research Ltd, Christchurch, New Zealand; 5 URGV, Evry, France; 6 United States Department of Agriculture-Agricultural Research Service Western Regional Plant Introduction Station, Pullman, Washington, United States of America; The University of North Carolina at Chapel Hill, United States of America

## Abstract

**Background:**

The genetic regulation of flower color has been widely studied, notably as a character used by Mendel and his predecessors in the study of inheritance in pea.

**Methodology/Principal Findings:**

We used the genome sequence of model legumes, together with their known synteny to the pea genome to identify candidate genes for the *A* and *A2* loci in pea. We then used a combination of genetic mapping, fast neutron mutant analysis, allelic diversity, transcript quantification and transient expression complementation studies to confirm the identity of the candidates.

**Conclusions/Significance:**

We have identified the pea genes *A* and *A2*. *A* is the factor determining anthocyanin pigmentation in pea that was used by Gregor Mendel 150 years ago in his study of inheritance. The *A* gene encodes a bHLH transcription factor. The white flowered mutant allele most likely used by Mendel is a simple G to A transition in a splice donor site that leads to a mis-spliced mRNA with a premature stop codon, and we have identified a second rare mutant allele. The *A2* gene encodes a WD40 protein that is part of an evolutionarily conserved regulatory complex.

## Introduction

The segregation of flower color in the progeny of pea crosses is well known in genetics because of Mendel's experiments [1], but it is less widely known that about 70 years previously Knight had studied the inheritance of flower color in pea [2]. Probably Mendel was aware of Knight's work and it may have helped him to choose his material for study. According to Fisher [3] it was in the spring of 1860 that Mendel counted the segregation ratios for this pigmentation character, immortalized in his famous ratio *A* + 2*Aa* + *a* [1]; *A* later became the symbol for the gene that determines the accumulation of anthocyanin pigmentation throughout the plant, most notably in flowers.

White flowered cultivated forms of pea are common and were available to Knight and Mendel, but wild peas have purple flowers, presumably as did the earliest cultivated forms. This raises the question of when white flowered types arose. An early description of ‘white peas’ in agriculture appears in Ruralia Commoda, written at the end of the 13th century or beginning of the 14th century by Pietro de Crescenzi in which he describes when to sow ‘large white’ peas [4]. In this context white probably refers to the seed coat, a component of the phenotype of *a* mutants, so by inference it refers to the existence of white flowered peas in agriculture; therefore white flowered forms have been in existence for at least 700 years, but the precise date of their origin remains unknown.

The purple color that accumulates in wild type pea flowers is due to anthocyanin pigments; compounds derived from phenylalanine. Mutations in either structural or regulatory genes have been shown to lead to a loss of pigmentation in several plant species [5], [6], [7], [8]. Genes corresponding to either MYB or basic-helix-loop-helix (bHLH) transcription factors or WD40 proteins are known regulators of anthocyanin biosynthesis [9] and previous investigations in pea have suggested that the white flower character determined by the recessive allele *a*, is due to the disruption of a regulatory gene [10]. Pea has not been the subject of a genome sequencing project, but its genome is known to be extensively collinear with that of *Medicago truncatula* [11], [12], for which genome sequence data is available [13]. We investigated whether any regions of the *M. truncatula* genome containing candidate genes were syntenic with the *A* locus in pea. For example, the four genes, *Legume Anthocyanin Production* (*LAP*)*1–4*, with sequence similarity to the MYB anthocyanin regulatory clade [14] are located on *M. truncatula* chromosomes 5, 7 and 8, none of which is syntenic with linkage group II of pea. This analysis eliminated all but one candidate bHLH gene that we studied further.

## Results

### The *A* gene is a bHLH transcription factor homolog

**Figure 1:**
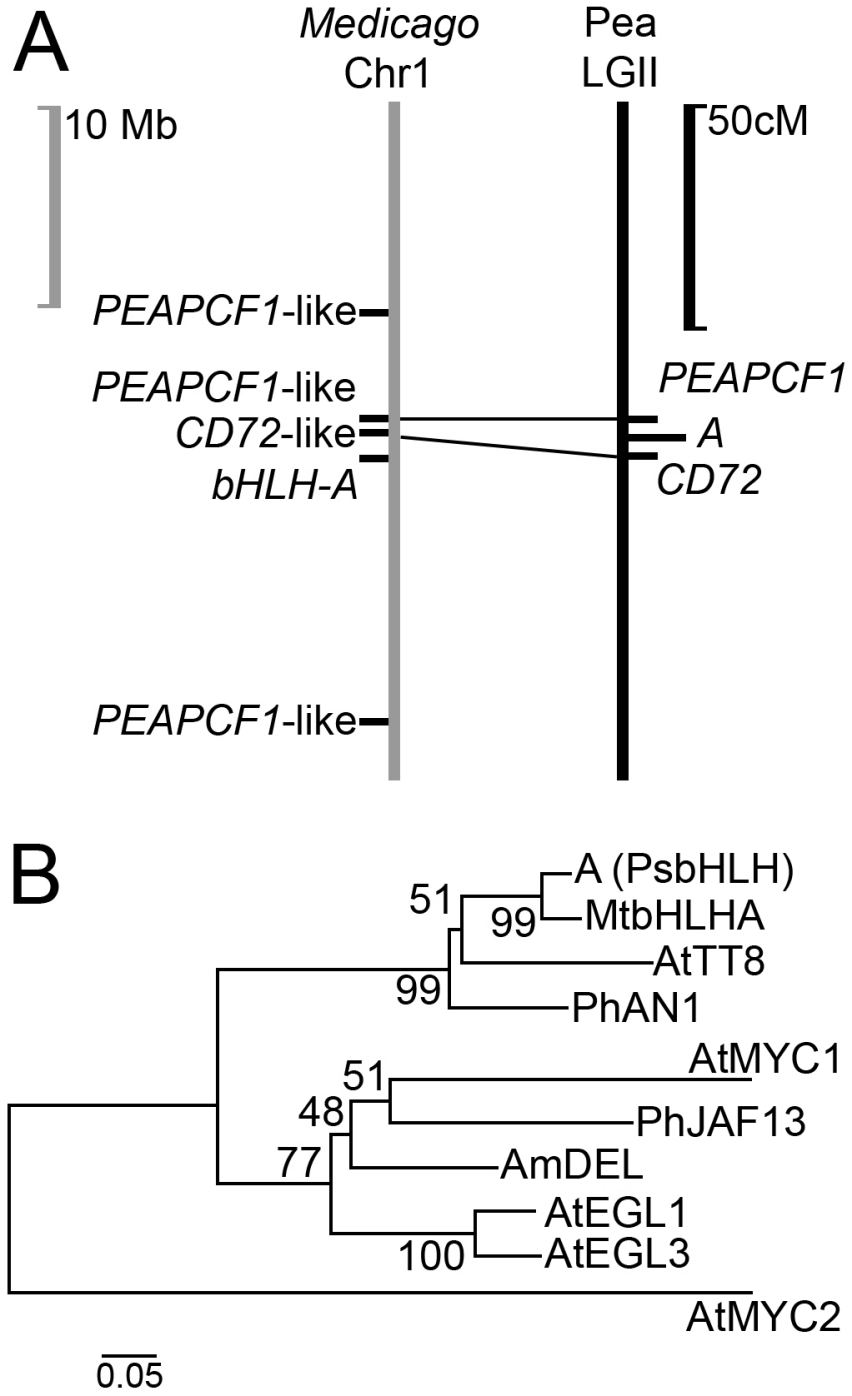
The *A* locus of pea. (*A*) A region of synteny between the genetic map of pea and the whole genome sequence of *M. truncatula* was identified using the sequences corresponding to two markers (PEAPCF1 [GU176398] and PsCD72 [Y11207]), closely linked to the *A* locus. A scale in Mb is given to the left, and in cM (Haldane) to the right. (*B*) Minimum evolution tree of the N-terminal region of the inferred amino acid sequence of bHLH proteins involved in regulation of phenylpropanoid biosynthesis: *Antirrhinum majus*: AmDEL [AAA32663], *Petunia hybrida*; PhJAF13 [AAC39455] and PhAN1 [AAG25928], *Arabidopsis thaliana*: AtEGL3 [NP_680372], AtEGL1 [Q9CAD0], AtTT8 [CAC14865] and AtMYC1 [NM_116272.3], *Medicago truncatula*: MtbHLHA [GU132940] and pea A (PsbHLH) [GU132941]. The tree is rooted with AtMYC2 [NP_174541]. Partial amino acid sequence alignments are presented in Fig. S6.

Two cDNA-RFLP markers (CD72 and PEAPCF1) are closely linked to the *A* locus on linkage group II of pea [15] and the corresponding sequences [Y11207 & GU176398] identify one contiguous, syntenic and collinear region of the *M. truncatula* genome sequence (Fig. 1A). A bHLH transcription factor gene lies within 1 Mb of the region defined by these pea markers (Fig. S1). In petunia, mutations of the *bHLH* gene *ANTHOCYANIN1* (*AN1*) are responsible for a white flower phenotype [6] and similar genes have been linked to anthocyanin accumulation or patterning in antirrhinum [5], maize [16] and *Arabidopsis thaliana* [17].

Degenerate primers designed to the *M. truncatula bHLH* gene were used to amplify corresponding sequences from pea and to identify an insertion/deletion (indel) distinguishing the parents of a mapping population [15] that segregates for purple versus white flower color. The indel in this pea *bHLH*-like sequence was shown to co-segregate with anthocyanin pigmentation in this population of 74 recombinant inbred lines (40 purple, 34 white, chi sq 0.49, n.s.).

Primers specific to the pea *bHLH* gene were then used to identify BAC clones derived from the purple flowered accession PI 269818 [18] and the white flowered cultivar Caméor. Two BACs, each over 150 kb, were sequenced and used to define a gene model for the *A* locus in pea and to identify the nature of the mutation in the white flowered variety. BAC 112D23 from the line PI 269818 contains an intact *bHLH* gene (Fig. S2 *A*); the gene model consists of seven exons in positions that are conserved with respect to *TRANSPARENT TESTA8* (*TT8*; AT4G09820), the closest homolog in *A. thaliana*. Amino acid sequence alignment of the N-terminal region of several bHLH proteins identifies the bHLH from pea to be in the same clade as AN1 and TT8 (Fig. 1*B*).

**Figure 2:**
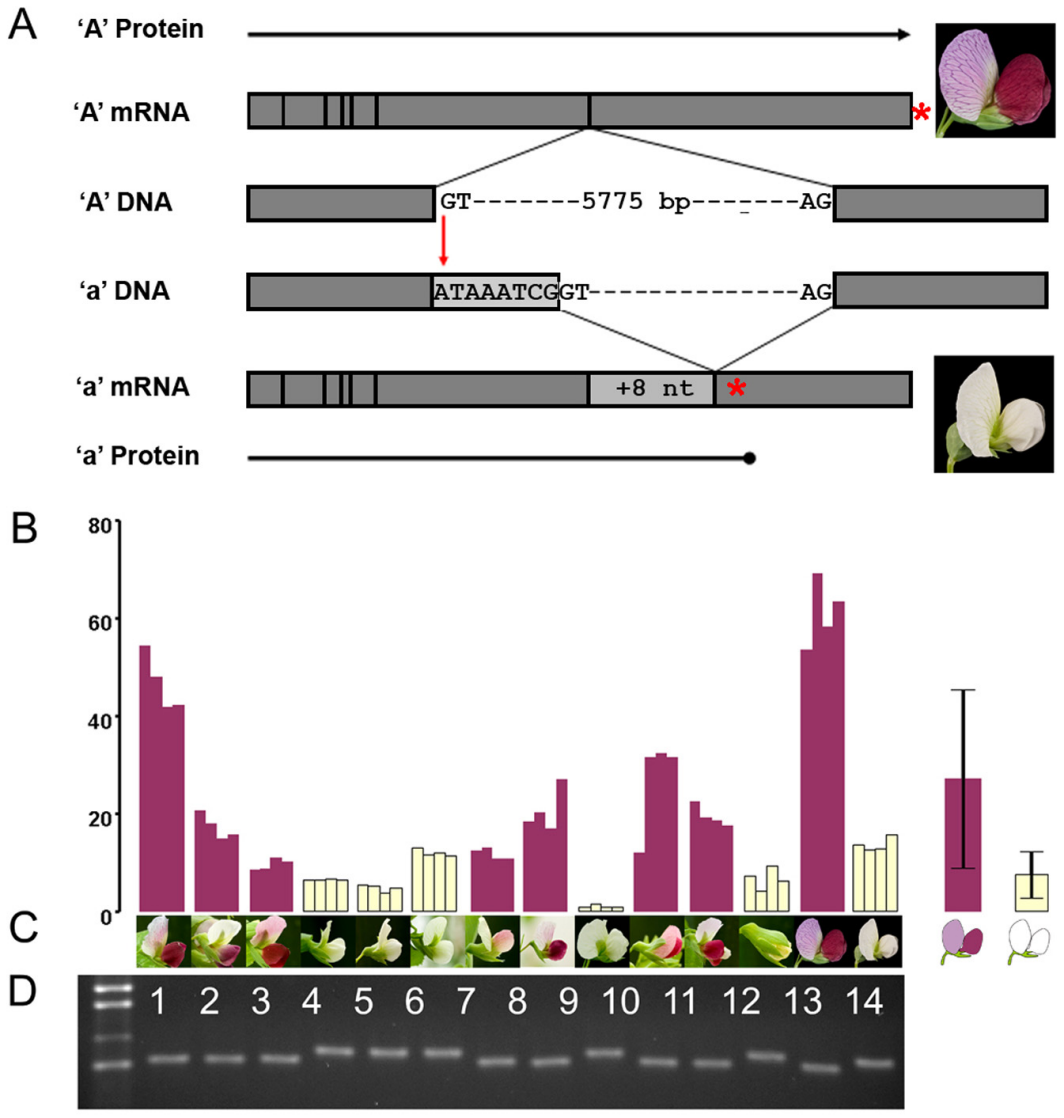
Molecular characterization of the *a* mutation in white flowered pea cultivars. (*A*) Schematic diagram showing the main features of the *bHLH* gene and its expression products (not to scale). In Caméor, a white flowered pea cultivar of genotype *a*, there is a single G to A mutation at the intron 6 splice donor site that disrupts the GT sequence required for normal intron processing. In the DNA, exons 6 and 7 are shown as grey boxes that flank the intron 6 splice donor and acceptor sequences. In the RNA, the vertical lines represent exon junctions and the light grey box represents the 8 nucleotide (nt) insertion in the *a* mRNA that results from mis-splicing of intron 6. The red stars show the position of the stop codon in the predicted protein, highlighting the premature termination in the white flowered cultivar. (*B*) Quantitative RT-PCR of *bHLH* in petal RNA from pigmented and white flowered Plant Inventory (PI) lines, 1; PI 174320, 2; PI 343331, 3; PI 343958, 4; PI 195404, 5; PI 279825, 6; PI 411143, 7; PI 174921, 8; PI 166159, 9; PI 116844, 10; PI 272204, 11; PI 155109, 12; PI 169603, 13; JI 2822, 14; Caméor. Transcript abundance relative to EF1α from pea was calculated according to the method of Pfaffl [36]. Individual experimental replications are shown. To the right of the figure the average for *A* and *a* alleles is shown with error bars showing one standard deviation (*A*: µ = 26.8, SD = 18.0, n = 8; *a*: µ = 7.5, SD = 4.7, n = 6). (*C*) Flowers from the twelve PI lines, JI 2822 and Caméor used in this analysis. (*D*) RT-PCR products spanning the exon6-exon7 junction. The molecular weight standard is shown in the far left. All eight colored flowered peas show a 79 bp amplification product and all white flowered peas show a larger, 87 bp product.

The equivalent *bHLH* gene, corresponding to an *a* allele, on BAC 452H2 from the white flowered cultivar Caméor, showed over 92% sequence identity to the *A* allele (Fig. S2 *A*). Sixteen SNPs were found when the predicted open reading frames of the *bHLH* sequences were aligned; three lead to amino acid changes and thirteen are silent mutations (Fig. S2 *B*,*C*). One of these silent mutations is a single base change, from G to A, in the splice donor site of intron 6 (Fig. 2*A* and Fig. S2 *A*,*B*). This G to A change was found only in white flowered lines while the other SNPs predicted to lead to amino acid changes were found in both colored and white flowered lines.

### A mis-spliced transcript is generated from an *a* allele

In plants, the GT splice donor site is present in almost all introns [19] and disruption of the sequence often leads to mis-splicing [20]. The sequence of a cDNA from the white flowered line Caméor has an additional eight nucleotides that the gene model assigns to intron 6. This is consistent with the G to A transition disrupting the splice donor site of intron 6 and the spliceosome identifying the next available GT motif, eight nucleotides downstream (Fig. 2*A*). These additional eight nucleotides are predicted to result in a frame-shift and a premature stop in the encoded polypeptide after the inclusion of eight amino acids unrelated to the wild-type (Fig. S2 *C*).

The presence of a premature stop codon may reduce mRNA accumulation through nonsense-mediated decay. Accordingly, we studied transcript accumulation from the *A* locus in eight white and eight colored flowered lines. This showed that the amount of bHLH RNA in white flowers was generally lower than in colored flowers (Fig. 2*B*, *C*). Some white flowered lines accumulated more transcript than colored flower lines, suggesting that other factors in the background may be more important than nonsense-mediated decay. Amplification across the exon 6–7 boundary confirmed an increased fragment length in the cDNA of these white flowered lines, consistent with the proposed mis-splicing (Fig. 2*D*).

Intron six has ∼5770 bases with ∼190 single nucleotide changes. The synonymous mutation rate for pea has been estimated as 7±2.6×10^−9^ nucleotides/yr [21], which implies that these *bHLH* alleles diverged ∼4 million years ago. This is unlikely to be the divergence time of the mutation itself, but the divergence time between two lineages, one of which subsequently acquired the mutation responsible for the white flowered phenotype.

### The classes and distribution of *a* alleles in pea cultivars

We were interested in whether we could find any examples of *a* alleles likely to have been available to Knight or Mendel. The John Innes *Pisum* accessions JI 2462 (Knight's Marrow) and JI 2479 (Knight's Dwarf White) are white flowered cultivars, and represent selections of Knight's material; these two lines carry the G to A transition at the splice donor site, providing further evidence that this *a* allele was available in Europe at the beginning of the 19^th^ century.

A systematic search of exotic *P. sativum* germplasm [22] identified nine white flowered lines. One of these, the Ethiopian accession JI 1987, did not carry the expected G to A mutation at the splice donor site. Sequencing the JI 1987 gene and cDNA revealed an additional nucleotide in exon 6 that would result in a frame shift with a premature stop codon (Fig. S3*A*). Sequence close to the intron 6 splice donor site showed that the JI 1987 *a* allele is similar to the wild type allele in JI 281 which is also an Ethiopian *P. sativum*. This is consistent with the JI 1987 *a* allele having arisen in Ethiopia, so it is unlikely to have been used by Mendel.

We analyzed the sequence of the *A* gene in 148 accessions in order to obtain an estimate of the frequency of *a* alleles that are explained by the splice site variant. This set, which comprised 88 white flowered and 60 colored flowered lines, includes 129 single plants from the pea refined core collection of the Plant Inventory (PI) lines held at USDA ARS, as well as PI 269818 and Caméor used for the construction of BAC libraries, together with 17 lines from the John Innes *Pisum* germplasm collection. All the lines with colored flowers had an intact splice donor site for intron 6 as expected. Of the white flowered lines, 78 carried the intron 6 splice site mutation, indicating that it is common (Fig. *S3B*). The sequenced region of these was identical for all but one accession (Fig. *S3C*). The 10 white flowered lines (including JI 1987) that did not carry the deviant splice donor site were of diverse provenance; three were collected in Ethiopia (JI 1987  =  PI 358622, PI 193578 & PI 331413), one in Uganda (PI 343824), one in Mexico (PI 142775) and one in French Guinea (PI 184784). Two lines are from breeders material (PI 266070, from Sweden and one, PI 269825, from the UK). PI 206861 is from the USA and PI 198074 from Germany. Of these 10 white flowered lines 7 carry the JI 1987 indel and three do not. Sequence data groups the three uncharacterized white flowered lines with known wild types (Fig. *S3C*) so it is possible that these carry wild type *A* alleles.

### The *A2* gene

Synteny analysis was used to identify gene candidates for *A2* [23]. Both MYB and WD40-like genes are located in the central region of *M. truncatula* chromosome 3, syntenic with LgIII in pea where the *A2* locus resides. BAC clone MTH2-174D3 corresponds to sequence AC146760 which encodes a MYB transcription factor. Analysis of the corresponding sequence from pea failed to identify significant differences between *A2* and *a2* lines.

The nearby BAC clone MTH2-28N4 (CR940305), contains sequence that encodes a WD40-like protein. The corresponding pea sequence was obtained from JI 2822 (Fig. S4) and primers designed to the 3′ end of the gene failed to generate an amplicon from FN 3690, a white flowered fast neutron mutant generated in the line JI 2822 [24]; this mutation is allelic to *a2*. The white flowered fast neutron mutant FN 3171 is in the same complementation group and carries a 22 bp deletion in the predicted WD40 coding sequence (Fig. S4), while PCR screens suggest that FN 3690 has a rearrangement with breakpoints within the gene. Two *a2* mutant lines (JI 2673 and JI 3062) were compared to their corresponding wild types, cv Melrose (JI 2208) and JI 3061. The *a2* mutant lines carried mutations in this WD40 gene; a G to A SNP (in JI 2673) that generates a W112Stop codon change in the N terminal domain of the predicted WD40 protein and a 5 bp deletion (in JI 3062) that makes a synonymous change at codon 222 generating a premature stop at codon 223. These four alleles of *WD40* in *a2* mutant lines suggest that *A2* corresponds to a WD40 protein.

We characterized the *WD40* gene in the three PI lines for which no mutation in the *A* gene has been found (PI193578, PI198074 and PI266070). These sequences were identical to that of JI 2822 except for a 115 nucleotide deletion in PI 198074 located 227 nucleotides upstream of the predicted ATG initiation codon. Clarification of the consequences of this deletion requires additional investigation.

### Rescuing the white flowered mutant phenotype

**Figure 3:**
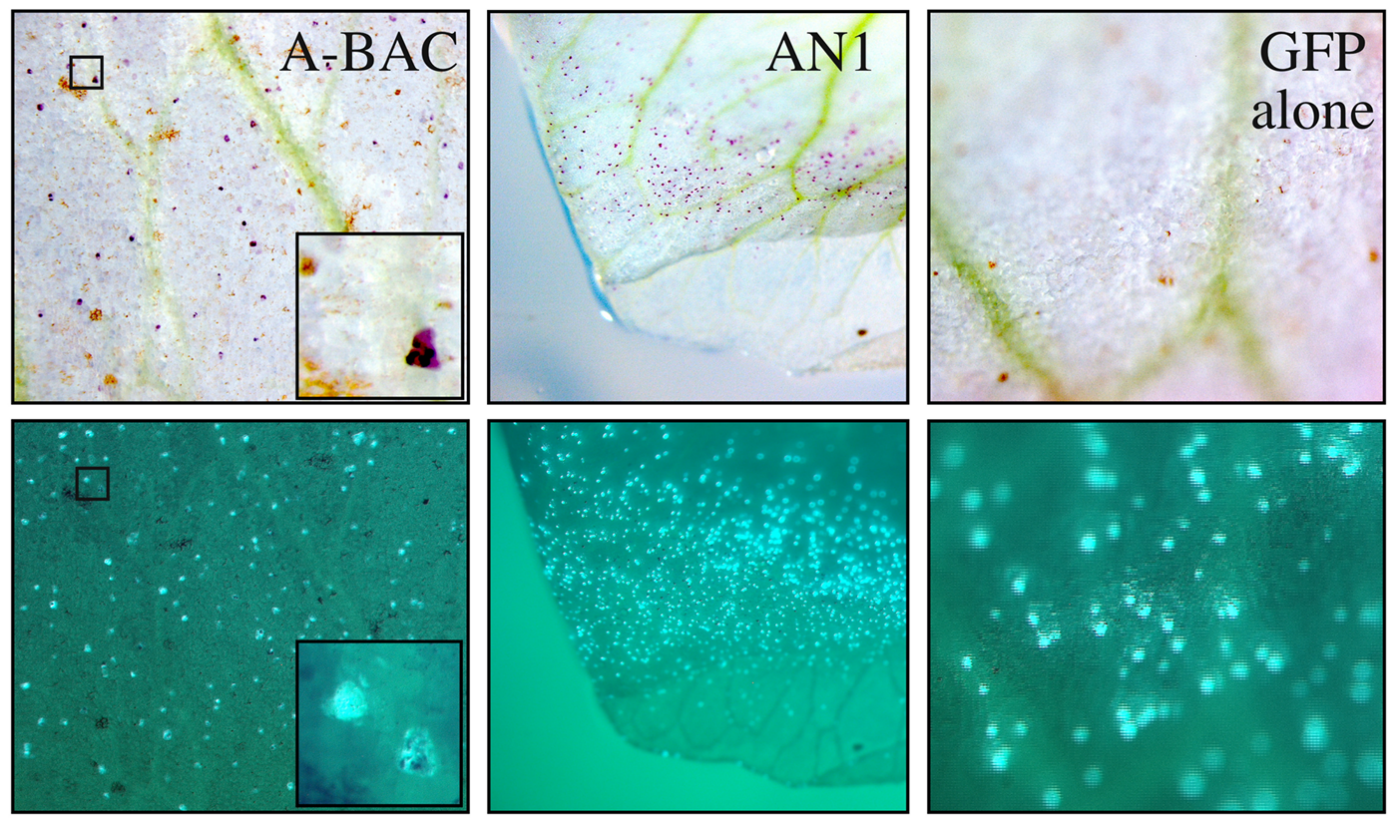
Complementation of white pea petals by particle bombardment. Particle bombardment of petals of Greenfeast (PI 250447 that carries the G to A splice donor mutation) with [left to right] the BAC containing the pea *bHLH* gene (A-BAC), over-expression cassettes for PhAN1 [AAG25928] (AN1) and GFP with no transcription factor (GFP alone). All experiments included an over-expression cassette of green fluorescent protein (GFP) co-precipitated onto the gold particle prior to bombardment. Anthocyanin accumulation is shown (*upper panels*). Fluorescence due to expression of the GFP protein (*lower panels*) was recorded two days after bombardment.

In order to test the hypothesis that the *bHLH* gene we have identified does indeed correspond to the *A* gene we complemented the white flower phenotype using transient over-expression (Fig. 3). Forty-eight hours after bombardment of white flowered pea petals with a positive control construct for petunia *AN1* numerous purple-colored cells were seen (Fig. 3). Similarly, bombardment with BAC DNA from the purple flowered line PI 269818, which includes a functional *bHLH* gene, also generated cells that accumulated anthocyanin (Fig. 3). In contrast, bombardment with a *green fluorescent protein* (*GFP*) construct alone did not restore pigmentation (Fig. 3). Bombardment with a construct for an anthocyanin MYB regulator of petunia, *AN2*, induced some colored cells, but comparatively few compared to the *bHLH* genes (Fig. S5). All pigmentation gene constructs were co-bombarded with *GFP*, so that fluorescence under blue light indicated those cells which had received *GFP* DNA.

## Discussion

We have established that a gene encoding a bHLH transcription factor is present at the *A* locus in pea and that this *bHLH* gene is disrupted in two distinct *a* alleles. We were able to rescue pigment formation by particle bombardment with the wild type gene or the orthologous *bHLH* gene from petunia. Uimari and Strommer [25] showed that maize *bHLH* genes (*R-S* and *Lc* from maize) can direct anthocyanin synthesis in pea when bombarded into petals of *a* genotypes. Surprisingly, their study also showed that these *bHLH* genes rescued the phenotype of *a2* mutants. The failure of complementation of either *a* or *a2* by *Antirrhinum* and petunia *bHLH* genes further complicated interpretation [25]. Our results, including complementation of the *a* mutation with BAC 112D23 from PI 269818, establish *A* as a *bHLH* gene. The identification of *A2* as a WD40 protein is of interest because the conservation of a transcription factor complex including bHLH and WD40 proteins in the regulation of anthocyanin pigmentation in plants suggests that a MYB transcription factor is probably also present in the complex in pea. If this MYB transcription factor is encoded by a single gene we would expect that mutations in the gene encoding this protein would also give a white flowered phenotype.

The cultivar Greenfeast carries the *a* allele with the intron 6 splice donor site mutation, and we know that the transcript from this *a* allele includes 8 nucleotides not present in the wild type due to mis-splicing. From our transcript abundance experiments (Fig. 2) it is not clear whether these transcripts exhibit nonsense-mediated decay. Transcript abundance in *a* lines is usually lower than in wild type lines, but the variance in transcript abundance in diverse *A* lines is much higher than for *a* lines, and the lowest abundance measured for an *A* allele is lower than the highest value for an *a* allele (Fig. 2). The process that regulates this transcript abundance is not clear, nor is the reason for the generally low transcript abundance in *a* genotypes. Intron 6 is the final intron in this gene, which would be consistent with the message evading the nonsense-mediated decay pathway.

There are many sequence variants of dominant *A* alleles, represented by 14 different haplotypes from the region around the intron 6 splice donor site. This variation is consistent with what is already known about the age of alleles in *Pisum* [21] and the diversity of *Pisum* germplasm [22]. Two allelic forms of the gene confer a white flowered phenotype. One, the intron 6 splice donor site mutation, is the predominant allele found in cultivars we have examined and the other is found in land races or breeders lines. Alleles in white flowered types are represented by a restricted set of haplotypes; for the intron 6 splice donor site mutation there are just two haplotypes in the vicinity of the mutation (Fig. *S3C*). One *a* haplotype is found in 77 accessions and the other is represented by a single sequence of an accession (PI 210558) that was collected in China. Interestingly, sequence downstream of the splice donor site in this allele matches that of a group of *A* alleles (Fig. S3C) consistent with the PI 210558 allele having arisen from a recombination event that occurred within 14 nucleotides of the splice site SNP.

Seven sequences represent the JI 1987 indel variant (Fig. *S3C*,*D*). Of these white flowered types three are of African origin, two from Ethiopia. The geographic distribution of *a* haplotype variation is consistent with a recent origin; once probably in Africa, and once in Eurasian peas, with the latter being subsequently adopted in cultivated forms. The available sequences of three white flowered individuals for which we have not identified a corresponding mutation can be classified along with two *A* haplotypes, which suggests these three may have wild type *A* alleles. One of these (PI 198074) may represent a promoter mutation of *A2* or all three may correspond to a mutation at another as yet unidentified locus.

### Mendel's allele

Both Mendel [1] and Knight [2] studied the segregation of white flower color in pea. It has been remarked that Mendel likely knew of Knight's work [26], and it is striking that in Mendel's 1866 paper he states three reasons for choosing pea as an experimental organism that are the same three reasons, in the same order, that Knight gave in 1799. We do not have a legacy of germplasm from Mendel's work, but Knight was a breeder and two lines, Knight's Marrow and Knight's White Dwarf, are held in the John Innes *Pisum* germplasm collection. Both these white flowered accessions carry the intron 6 splice donor site mutation, so this allele used by Knight was present in European peas prior to Mendel's studies. Since we know that the mis-spliced *a* allele was available and prevalent in cultivated material, we propose that this is the molecular basis of the mutation that was used by Mendel in his experiments on inheritance 150 years ago [1].

## Materials and Methods

### Plant Material

The following JIC lines were used: colored flower lines were, JI 15, JI 281, JI 813 and JI 2822, white flowered lines were JI 4, JI 399, JI 1194, JI 1201, Torsdag (JI 992) the Greenfeast accessions: JI 504, JI 1189, JI 1229 and JI 2737 and Knight's lines: JI 2462 and JI 2479. Exotic white flowered lines were; JI 232, JI 616, JI 817, JI 1497, JI 1512, JI 1782, JI 1987, JI 2647 and JI 3003; these correspond to white flowered *P.sativum* accessions from structure sub-groups that lie close to the trifurcation of *P.sativum, P.fulvum* and *P.elatius* [22].

A selection of PI lines from USDA ARS (see USDA ARS Germplasm Resources Information Network at: http://www.ars-grin.gov) were used for PCR experiments: PI 174320, PI 343331, PI 343958, PI 195404, PI 279825, PI 411143, PI 174921, PI 166159, PI 116844, PI 272204, PI 155109 and PI 169603.

The RIL mapping population derived from the JI 281×JI 399 cross corresponds to the accessions numbered JI 2868 to JI 2939.

In addition, cultivar Caméor (white) and line PI 269818 (colored) [18] were used for the construction of BAC libraries, sequence analysis and construction of the gene model.

### Bioinformatics

A TBLASTN [27] search of the *M. truncatula* genome pseudomolecule using the chromosome visualization tool CViT BLAST identified sequences that showed similarity between pea and *M. truncatula* (http://www.medicago.org/).

Full length *bHLH* sequences were aligned (Fig. S6) using ClustalW (opening = 15, extension = 0.3) in Vector NTI 9.0 (Invitrogen). Conserved motifs were extracted and re-aligned as above. Phylogenetic and molecular evolutionary analyses were conducted using MEGA [28] version 4.0.2. A Minimum Evolution tree was generated by the Neighbor-Joining method with level 1 Close-Neighbor-Interchange. Bootstrap support was tested with 1000 replications (76752 random seeds).

### Amplification and mapping of the pea *bHLH* gene

Primers RPH-445 – RPH-454 (Table S1) were designed to the Mt*bHLH* gene [GU132940] and used in low annealing temperature PCR with pea DNA templates (JI 281 and JI 399): 94°C, 5 min followed by 35 cycles of 30 s at 94°C, 45 s at 50°C and 150 s at 72°C. Amplification fragments were cloned into pGEMT-Easy (Promega) according to the manufacturer's instructions. Sequences from the amplification product of RPH-447/RPH-448 identified PCR amplicon length polymorphisms between JI 281 and JI 399 to which primers PsbHLHintF and PsbHLHintR (Table S1) were designed. These were used to determine the map location of the *PsbHLH* gene in a recombinant inbred line population derived from the cross between JI 281 and JI 399 [15].

### BAC analysis

BAC clone 452H2 was identified using primers PsbHLHintF and PsbHLHintR. BAC clone 112D23 was identified from BAC pools [18] by screening nitrocellulose filters with a probe amplified from PI 269818 using primers RPH_453 and RPH_452 (Table S1).

Sanger sequencing of pea BAC clones 112D23 and 452H2 was performed by GATC (Germany) and sequences were assembled using both ContigExpress and the Staden Pregap package as previously described [29], integrated with Phred and Phrap [30] for base calling and assembly and Gap4 [31] for editing. The assemblies were subsequently aligned and compared using MUMmer [32] and Mauve [33]. Transcript sequences were aligned to the genome sequence using MUSCLE [34]. Further sequencing with primers PsbHLHA-F1 to -F15 and PsbHLHA-R1 to -R15 (Table S1) was necessary to confirm the assembly of the *bHLH* gene in BAC 452H2.

The sequence GU132941 corresponds to the BAC clone 112D23 derived from PI 269818 (*A*) and GU132942 to the BAC clone 452H2 derived from cv Cameor (*a*).

### Allele characterization in PI lines

PCR products containing the junction of exon 6 and intron 6 of 129 PI lines (Fig. *S3B*) were amplified with primers bHLH_5432_L and bHLH_6089_R (Table S1). The resulting sequence is presented in Fig. *S3C*. These sequences are available as accessions HQ245159-HQ245315.

### qRT-PCR analysis

Total RNA was isolated from flower tissue of both colored and white flowered pea varieties (Plant RNA Reagent, Invitrogen). All RNA samples were treated with DNase (DNA-free Kit, Ambion) to remove contaminating DNA. First strand cDNA synthesis was carried out using SuperScript III and Oligo dT according to manufacturer's instructions (Invitrogen).

Quantitative real-time PCR (qPCR) amplification of cDNA and analysis was carried out using the LightCycler 480 System (Roche), with LightCycler software version 1.5. Primers KL244F and KL246R (Table S1) and LightCycler 480 SYBR Green I Master Mix (Roche) were used in 10 µl total reaction volumes following the manufacturer's methods. qPCR conditions were 5 min at 95°C, followed by 40 cycles of 10 s at 95°C, 10 s at 60°C, and 20 s at 72°C, followed by 65°C to 95°C melting curve detection. Primers PsEF1F and PsEF1R, were used to amplify pea elongation factor 1α (EF1α, X96555). This was selected as a reference gene because of its consistent transcript level among varieties. The qPCR efficiency of each primer pair was obtained by analyzing the standard curve of a cDNA serial dilution of the corresponding gene.

### PCR analysis of cDNA length variation

PCR analysis of lines examined for mRNA accumulation was carried out using Platinum Taq (Invitrogen). Primers KL255F and KL256R were designed to amplify a cDNA region from 40 bp upstream to 39 bp downstream of the intron 6 splice donor site. Reaction conditions were 95°C, 5 min followed by 35 cycles of 30 s at 95°C, 30 s at 57°C and 20 s at 72°C. PCR products were separated on 3% agarose gels along with 1 kb ladder (Invitrogen) and stained with ethidium bromide.

### Particle bombardment experiments

DNA was bombarded into the abaxial surface of wing petals from white flowered Greenfeast grown in the glasshouse. DNA (5 µg with 2 µg of internal control GFP construct) was precipitated onto 5 mg of gold particles (1.0 µm diameter, in 50 µl sterile water), and tissue was surface sterilized prior to bombardment [7]. Tissue was bombarded twice with 5 µl of the gold suspension using a particle inflow gun [35] (Kiwi Scientific, Levin, New Zealand) with the conditions: 30 ms burst of helium (200 kPA) within a vacuum of −90 kPA and tissue at a range of 12 cm. After bombardment tissue was incubated on MS medium at 20±3°C in a 16-h-light/8-h-dark photoperiod with 20–50 µmol⋅m^−2^s^−1^ cool-white fluorescent light. Constructs were pPN73 (petunia AN2 Myb), pKES14 (petunia AN1 bHLH), pRT99GFP (m-GFP5-ER). The BAC DNA used was 112D23 from the purple flowered line PI 269818.

## Supporting Information

Figure S1 Synteny between the pea genetic map and *M. truncatula* genome sequence. PEAPCF1 [GU176398] and CD72 [Y11207] flank the A locus in the pea genetic map. In the *M. truncatula* genome, BACs with the best BLAST matches to these pea genes are indicated by arrows along with BAC contigs and the genetic markers that were used to assemble the physical map. BAC contig and scaffold assembly for v3.0 of the *M. truncatula* genome can be found on the Medicago genome website (03-15-2009 update), http://www.medicago.org/genome/contig_viewer.php?accession=AC150981.
(0.42 MB DOC)

Figure S2 Sequence alignment of the A gene. (A) Alignment of genomic DNA of pea bHLH genes from colored flowered A line PI 269818 BAC 112D23 [GU132941] and white flowered a cultivar Caméor BAC 452H2 [GU132942]. The sequence shown for the exons is that of PI 269818 as this is known to be a functional allele. Exons 1–7 are marked and nucleotide differences between PI 269818 (A) and Caméor (a) alleles are highlighted. The G to A mutation in a at base 5005 that leads to the mis-splicing of intron 6 is marked with an asterisk (*). (B) Alignment of predicted mRNA from PI 269818 (A) and Caméor (a) genotypes. ATG start codons and TAG stop codons are marked and in bold. Nucleotide differences are highlighted. The first 8 bases of intron 6 that are included in mRNA of the Caméor (a) genotype are marked with asterisks (********). (C) Alignment of the predicted bHLH coding sequence from purple flowered A (PI 269818 and JI 2822) and white flowered a genotypes (Caméor and JI 1987). Amino acid sequence differences are highlighted and stop codons are marked with asterisks (*).
(0.14 MB DOC)

Figure S3 Sequence divergence of the bHLH gene in germplasm and exotic pea lines. (A) Sequence of exotic pea lines. For JI 4 and 9 exotic white flowered pea lines; JI 232, JI 616, JI 817, JI 1497, JI 1512, JI 1782, JI 1987, JI 2647 and JI 3003 the G to A mutation is highlighted and marked with *. Three independent individuals of the white flowered JI 1987 (n1, n2 and n3) do not have the G to A transition, they do, however, have a number of SNPs that are highlighted in light blue and an additional A residue in exon 6, identified by the # and highlighted in red. (B) Association of pea flower color with the G to A mutation at base 5005 (highlighted) in 148 lines (including 129 single plant representatives from the refined core of PI lines see USDA ARS Germplasm Resources Information Network at: http://www.ars-grin.gov). All colored flower lines (n = 60) carry the G allele that corresponds to correct splicing of intron 6, while most white flowered lines (n = 78) have the A mutation, resulting in mis-splicing. Ten white flowered lines carry the G allele, and seven of these have been found to carry the JI 1987 indel allele. For three white flowered lines neither mutation has been identified. (C) Flower color phenotype and alignment of SNPs at the junction between exon 6 and intron 6 of the bHLH gene of 148 pea lines. Within a window of 145 nt including the splice donor site (at nt 22) there are 21 SNPs and one indel (GTTGTATTTAG, present only in PI 273209) that are shown. The first SNP is in exon 6 and the second is the G/A transition at the intron 6 splice donor site. Polymorphic nucleotides are colored to make the haplotypes easy to see. The column to the left indicates flower color pink = colored flower, white = white. The lines with the splice donor site mutation and the JI 1987 indel are indicated. The three sequences for which a mutation in the A gene has not been identified are arrowed. To the right an unweighted UPGMA tree based on these sequences is shown and the accessions have been ordered according to a neighbor joining tree constructed on the basis of this tree. The order of accessions (top to bottom) is: PI 102888, PI 116056, PI 125840, PI 143485, PI 116944, PI 125839, PI 198735, PI 207508, PI 220174, PI 220189, PI 222071, PI 222117, PI 340130, PI 210558, PI 103058, PI 116844, PI 117264, PI 121352, PI 124478, PI 134271, PI 156720, PI 163126, PI 163129, PI 164182, PI 164548, PI 164612, PI 169603, PI 171810, PI 172339, PI 173840, PI 179459, PI 179970, PI 180696, PI 181799, PI 181801, PI 181958, PI 184130, PI 193584, PI 195404, PI 197044, PI 201390, PI 203066, PI 203067, PI 203068, PI 203069, PI 206838, PI 209507, PI 210561, PI 210568, PI 210569, PI 210571, PI 210583, PI 212031, PI 212917, PI 221697, PI 242028, PI 244093, PI 248181, PI 257244, PI 261624, PI 263027, PI 269798, PI 269821, PI 271035, PI 271511, PI 279825, PI 280603, PI 280611, PI 280616, PI 285715, PI 286431, PI 286607, PI 343987, PI 411143, JI 232, JI 616, JI 871, JI 1497, JI 1512, JI 1792, JI 2647, JI 3003, JI 4, Cameor, JI 504, JI 1189, JI 1229, JI 2737, JI 2462, JI 2479, Torsdag, PI 169608, PI 180693, PI 184128, PI 193590, PI 195020, PI 195631, PI 257592, PI 269782, PI 272204, PI 272218, PI 285719, PI 331414, PI 340128, PI 343331, PI 270536, PI 280619, PI 193578, PI 179450, PI 142775, PI 184784, PI 206861, PI 269825, PI 331413, PI 343824, JI 1987, PI 109866, PI 137118, PI 137119, PI 140298, PI 155109, PI 156647, PI 164972, PI 174320, PI 180329, PI 180702, PI 197990, PI 198072, PI 204306, PI 343958, PI 198074, PI 266070, PI 117998, PI 179451, PI 203064, PI 164971, PI 188698, PI 242027, PI 269818, PI 162909, PI 179722, PI 165949, PI 166084, PI 166159, PI 174921, PI 164779, PI 271033, PI 273209. (D) FASTA sequence of the region between exon 6 and intron 6 that was used for tree construction.
(0.37 MB DOC)

Figure S4 Annotated sequence of the A2 gene from JI2822 and showing differences in various lines. The sequence from nt 160 to nt 217 (bold italic) is a direct duplication, only one copy of which is found in the PI 198074 allele. From nucleotide 530 to 644 (bold italic) is also deleted in the PI 198074 allele. Note the GCACA sequence is repeated at either end of this deleted segment. The G to A SNP at nucleotide 1207 in JI2673 that changes TGG (W) to TGA (stop) is indicated in bold, cv Melrose in which this mutation arose has the sequence TGG as expected. The deletions from nucleotide 1360 to 1381 in FN3171 and from nucleotide 1510 to 1514 in JI 3062 are highlighted in bold italics. The line JI 3061, which carries the progenitor allele of this mutation, has the same sequence as JI 2822 at that point. The encoded amino acid sequence and other features of note are indicated. Four SNPs identified in the lines PI 193578 PI 198074 and PI 266070 are indicated in bold and ‘in PIs’ (all carry the same SNP with respect to JI2822).
(0.04 MB DOC)

Figure S5 Complementation of white pea petals by particle bombardment. Particle bombardment of petals of Greenfeast (PI 250447 that carries the G to A splice donor mutation) with [left to right], over-expression cassettes for PhAN1 (AN1), reproduced from Figure 3 for comparison, and PhAN2. All experiments included an over-expression cassette of green fluorescent protein (GFP) co-precipitated onto the gold particle prior to bombardment. Anthocyanin accumulation is shown (upper panels). Fluorescence due to expression of the GFP protein (lower panels) was recorded two days after bombardment.
(4.74 MB DOC)

Figure S6 Alignment of the predicted amino acid sequence of the N-terminal region of bHLH proteins.
(0.04 MB DOC)

Table S1 A) Primers used in the identification and characterization of the bHLH gene from pea (Ps) [GU132941] and *M. truncatula* (Mt) [GU132940]. (B) Primers used in the identification and characterization of the WD40 gene from pea.
(0.11 MB DOC)
