# Supplementary material for: Identification of Mendel's White Flower Character
Source: PLoS One. 2010 Oct 11;5(10):e13230. doi: 10.1371/journal.pone.0013230 (PMC2952588; doi:10.1371/journal.pone.0013230)

**Figure S1. Synteny between the pea genetic map and *M. truncatula* genome sequence.** *PEAPCF1* [GU176398] and *CD72* [Y11207] flank the *A* locus in the pea genetic map. In the *M. truncatula* genome, BACs with the best BLAST matches to these pea genes are indicated by arrows along with BAC contigs and the genetic markers that were used to assemble the physical map. BAC contig and scaffold assembly for v3.0 of the M. truncatula genome can be found on the Medicago genome website (03-15-2009 update) <http://www.medicago.org/genome/contig_viewer.php?accession=AC150981>


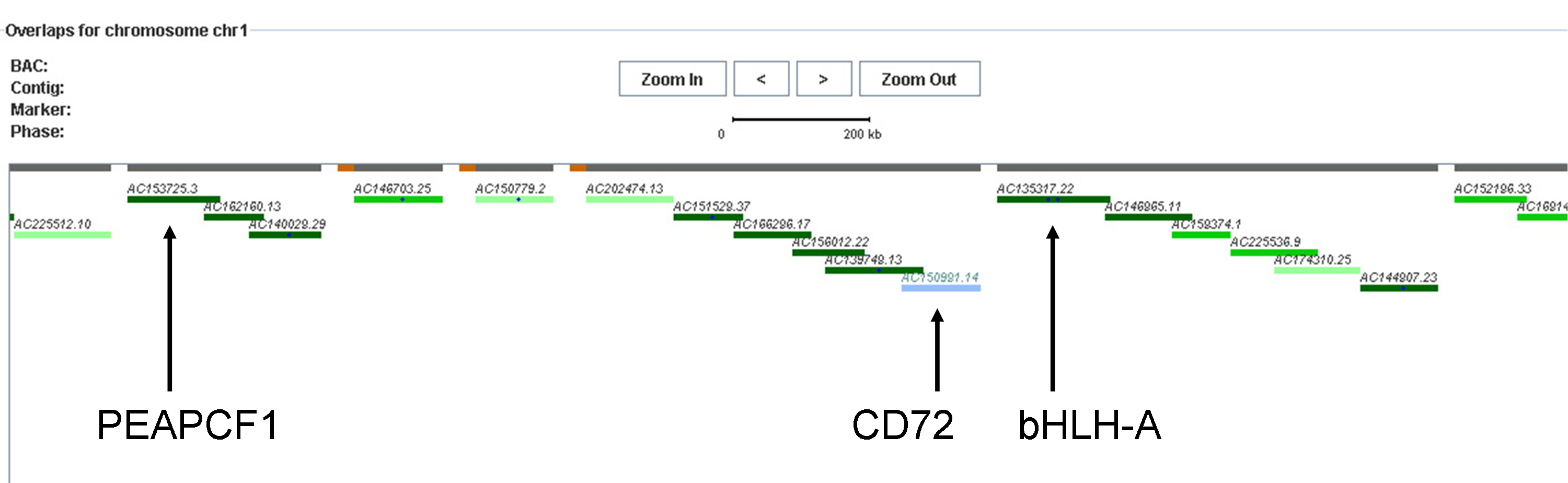

Supplement: Figure S1 — Synteny between the pea genetic map and M. truncatula genome sequence. PEAPCF1 [GU176398] and CD72 [Y11207] flank the A locus in the pea genetic map. In the M. truncatula genome, BACs with the best BLAST matches to these pea genes are indicated by arrows along with BAC contigs and the genetic markers that were used to assemble the physical map. BAC contig and scaffold assembly for v3.0 of the M. truncatula genome can be found on the Medicago genome website (03-15-2009 update), http://www.medicago.org/genome/contig_viewer.php?accession=AC150981. (0.42 MB DOC) [file pone.0013230.s001.doc]
