# Supplementary material for: Identification of Mendel's White Flower Character
Source: PLoS One. 2010 Oct 11;5(10):e13230. doi: 10.1371/journal.pone.0013230 (PMC2952588; doi:10.1371/journal.pone.0013230)
Supplement: Figure S2 — Sequence alignment of the A gene. (A) Alignment of genomic DNA of pea bHLH genes from colored flowered A line PI 269818 BAC 112D23 [GU132941] and white flowered a cultivar Caméor BAC 452H2 [GU132942]. The sequence shown for the exons is that of PI 269818 as this is known to be a functional allele. Exons 1–7 are marked and nucleotide differences between PI 269818 (A) and Caméor (a) alleles are highlighted. The G to A mutation in a at base 5005 that leads to the mis-splicing of intron 6 is marked with an asterisk (*). (B) Alignment of predicted mRNA from PI 269818 (A) and Caméor (a) genotypes. ATG start codons and TAG stop codons are marked and in bold. Nucleotide differences are highlighted. The first 8 bases of intron 6 that are included in mRNA of the Caméor (a) genotype are marked with asterisks (********). (C) Alignment of the predicted bHLH coding sequence from purple flowered A (PI 269818 and JI 2822) and white flowered a genotypes (Caméor and JI 1987). Amino acid sequence differences are highlighted and stop codons are marked with asterisks (*). (0.14 MB DOC) [file pone.0013230.s002.doc]

**Figure S2.** Sequence alignment of the *A* gene. (*A*) Alignment of genomic DNA of pea *bHLH* genes from colored-flowered *A* cultivar PI 269818 BAC112D23 [GU132941] and white flowered a cultivar Caméor BAC 452H2 [GU132942]. The sequence shown for the exons is that of PI 269818 as this is known to be a functional allele. Exons 1-7 are marked and nucleotide differences between PI 269818 (*A*) and Caméor (*a*) alleles are highlighted The G to A mutation in a at base 5005 that leads to the mis-splicing of intron 6 is marked with an asterisk (*).

1 100

*a*  (1) ---TTTTCTGATCTGTCGATGATGGGCCTGATGACAAATTATTTTGGGTTACTTGGCACATCCTGTGCCTTAGCTATTGTTTTTTTTAAAGTTGTATTCT

*A*  (1) GACTTTTCTGATCTGTCGATGATGGGCCTGATGACAAATTATTTTGGGTTACTTGGCACATCCTGTGCCTTAGCTATTGTTTTTTTTAAAGTTGTATTCT

101 200

*a*  (98) CCTTGCCCTTTTTCCTTGTATATATATTTCTATATCATTACTTCGCCTTTTAAGAAAAGAATTTAAACAAAGTATTGATTCTTATAAATTAATAGTTTAA

*A*  (101) CCTTGCCCTTTTTCCTTGTATATATATTTCTATATCATTACTTCGCCTTTTAAGAAAAGAATTTAAACAAAGTATTGATTCTTATAAATTAATAGTTTAA

201 300

*a*  (198) AAGAAATCACTCACATTTAAAATTAAAAAATATAGTCATTTAAATAGACCTAATAATGTTAATTTTTTTTAACCTTATAAAACTAACTATATTATTTTAT

*A*  (201) AAGAAATCACTCACATTTAAAATTAAAAAATATAGTCATTTAAATAGACCTAATAATGTTAATTTTTTTTAACCTTATAAAACTAACTATATTATTTAAT

301 400

*a*  (298) TTCTGTAAGACATGTTAAACTATCATATATTTCAAAAACATCATTGGCTAGTATCAATTGTTTTAATATATTTTAATTTAAAAAACTCAAATAATTCCCA

*A*  (301) TTCTGTAAGACATGTTAAACTATCATATATTTCAAAAACATCAT--GCTAGTACCAATTTTTTTAATATATTTTAATTTAAAAAACTCAAATAATTCCCA

401 500

*a*  (398) TCAAATTTATACTAAACTTTCATAATTTTTAAAATTAAAGATAAATTTTAATAATAATATTTTTTATTAAATAGACAATTTGTTGTTAGCTCTTGTCATT

*A*  (399) TCAAATTTATACTAAACTTTCATAATTTTTAAAATTAAAGATAAATTTTAATAATAATATTTTTTATTAAATAGACAATTTCTTGTTAGCTCTTGTCATT

501 600

*a*  (498) TTGAAAAAGGAAAAACAAGTTGGGTATGCATAGATTTCAATGTTCAAATATGGTAACTGTTGTGAAAATTAGTTTCACAACTAACTAACAAACCACAACT

*A*  (499) TTGAAAAAGGAAAAACAAGTTGGGTATGCATAGATTTCAATGTTCAAATATGGTAACTGTTGTGAAAATTAGTTTCACAACTAACTAACAAACCACAACT

601 700

*a*  (598) GTGATACTGGTACATATTATACATATAGAACCCAAAAACTGTCTGGTAGACCAACCAGTCAAAAATACTCCATAATCTCATACCCATCCATGCTACTCCT

*A*  (599) GTGATACTGGTACATATTATACATATAGAACCCAAAAACTGTCTGGTAGACCAACCAGTCAAAAATACTCCATAATCTCATACCCATCCATGCTACTCCT

701 800

*a*  (698) TATAATTTCATATCTCTTTTCTCTTTTCTTAACCAACAAACCTTCTTCCATAATCTCAACTACAAGCCACACCTGCAAGTCTACACATATATATATACTC

*A*  (699) TATAATTTCATATCTCTTTTCTCTTTTCTTAACCAACAAACCTTCTTCCATAATCTCAACTACAAGCCACACCTGCAAGTCTACACATATATATATACTC

801 |---5’UTR start 900

*a*  (798) CTTCGTTGAAAGTTCAGCTTACCACATGTTCTTTCCTTTAATTTTGCTGTTTCTCGCTTTTTTACTACTTTTGAAAGTTATGATTCTTTCTCAGAGAACT

*A*  (799) CTTCGTTGAAAGTTCAGCTTACCACATGTTCTTTC-TTTAATTTTGCTGTTTCTCGCTTTTTTACTACTTTTGAAAGTTATGATTCTTTCTCAGAGAACT

901 1000

*a*  (898) GAATGAGCCAAGAAGAATGACTGCTCCAACTCCGGAAAACGGTTGTAATAAGCTTCAAAACATGTTGCAGGCTGCAGTGCAATCGGTTCAATGGACTTAT

*A*  (898) GAATGAGCCAAGAAGAATGACTGCTCCAACTCCGGAAAACGGTTGTAATAAGCTTCAAAACATGTTGCAGGCTGCAGTGCAATCGGTTCAATGGACTTAT

Exon 1 ATGACTGCTCCAACTCCGGAAAACGGTTGTAATAAGCTTCAAAACATGTTGCAGGCTGCAGTGCAATCGGTTCAATGGACTTAT

1001 1100

*a*  (998) AGCCTCTTCTGGCAAATTTGCCCACAACAATTGTACAAATTTTTGTATTTTTTTTTATTTGGTCTTATACCAAAACACACTTTACTAAACTATATTATAA

*A*  (998) AGCCTCTTCTGGCAAATTTGCCCACAACAATTGTACAAATTTTTGTATTTTTTTT-ATTTGGTCTTATACCAAAACACACTTTACTAAACTATATTATAA

Exon 1 AGCCTCTTCTGGCAAATTTGCCCACAACAATT

1101 1200

*a*  (1098) TTATTTTGAATTGCATTTTAGGATACTGGTTTGGGGCGATGGATATTACAACGGAGCAATTAAGACACGAAAGACAGTGCAACCAATGGAGGTTAGTGCA

*A*  (1097) TTATTTTGAATTGCATTTTAGGATACTGGTTTGGGGCGATGGATATTACAACGGAGCAATTAAGACACGAAAGACAGTGCAACCAATGGAGGTTAGTGCA

Exon 2 GATACTGGTTTGGGGCGATGGATATTACAACGGAGCAATTAAGACACGAAAGACAGTGCAACCAATGGAGGTTAGTGCA

1201 1300

*a*  (1198) GAAGAAGCTTCTCTACAAAGAAGCCAGCAACTAAGAGAGCTGTATGAATCGTTATCGGCCGGAGAGACAAATCCGCCAACGCGTCGACCGTGTGCTTCTT

*A*  (1197) GAAGAAGCTTCTCTACAAAGAAGCCAGCAACTAAGAGAGCTGTATGAATCGTTATCGGCCGGAGAGACAAATCCGCCAACGCGTCGACCGTGTGCTTCTT

Exon 2 GAAGAAGCTTCTCTACAAAGAAGCCAGCAACTAAGAGAGCTGTATGAATCGTTATCGGCCGGAGAGACAAATCCGCCAACGCGTCGACCGTGTGCTTCTT

1301 1400

*a*  (1298) TGTCGCCTGAAGACTTAACAGAATCTGAATGGTTTTATTTGATGTGTGTCTCTTTCTCATTTCCTCCTGGTGTCGGGTATAATTCACCTTCTTCTATTAT

*A*  (1297) TGTCGCCTGAAGACTTAACAGAATCTGAATGGTTTTATTTGATGTGTGTCTCTTTCTCATTTCCTCCTGGTGTCGGGTATAATTCACCTTCTTCTATTAT

Exon 2 TGTCGCCTGAAGACTTAACAGAATCTGAATGGTTTTATTTGATGTGTGTCTCTTTCTCATTTCCTCCTGGTGTCGG

1401 1500

*a*  (1398) TTGTTACCTAGCTATAGCTCTCTCTCTTTTCATACTATGAATTTCATTCACCTATACTATATAGCTTTATCTTTAATGTACAACAGAAAATATTTAATAA

*A*  (1397) TTGTTACCTAGCTATAGCTCTCTCTCTTTTCATACTATGAATTTCATTCACCTATACTATATAGCTTTATCTTTAATGTACAACAGAAAATATTTAATAA

1501 1600

*a*  (1498) TATTTTAATATTTGAAAATTGTGATTGATTAACCATTTAAAATACGGTTGTAAACACACAATGCATGACAATTGAATTTGTATTTTTAGTCCAAAATTCA

*A*  (1497) TATTTTAATATTTGAAAATTGTGATTGATTAACCATTTAAAATACGGTTGTAAACACACAATGCATGACAATTGAATTTGTATTTTTAGTCCAAAATTCA

1601 1700

*a*  (1598) ACTGTCTTCTTTCACGGTGGTGATTGTGGTGTTCAATATGTGGAGGTTGGCTACTATGCTCATTTGACCACCATCACCTCTATTATTAATTCTTTTTTCA

*A*  (1597) AGTGTCTTCTTTCACGGTGGTGATTGTGGTGTTCAATATGTGGAGGTTGGCTACTATGCTCATTTGACCACCATCACCTCTATTATTAATTCTTTTTTCA

1701 1800

*a*  (1698) AATAGTCTATCGTTTAGAATTTAAAATGAATAAATAGGATGTCACGGTTCGAACTCGCAGAACTACCGCTGGATTAACTGGACATCATTATTATTAAATT

*A*  (1697) AATAGTCTTTCGTTTAGAATTTAAAATGAATAAATAGGATGTCACTGTTCGAACTCGCAGAAGTACAGTTGGATTAACTGGACATCATTATTATTAAATT

1801 1900

*a*  (1798) AATAGTAACTTAATGTTTTTTAAAGATAAATTAGTTATTTATTTAAAGATTTTCAATAAGATTTTAAATAGGTGTAATAGATGAAACATTAACCTCATTT

*A*  (1797) AACAGTAACTTAATGCTTTTTAAAGATAAATTAGTTATTTATTTAAAGATTTTCAATAAGATTCTACATAGGTGTAATAGATGAAACATTAACCTCATTT

1901 2000

*a*  (1898) TAAATTTTACTTATAGTTTACAATTATTTCTGTATAATAATTTATACTCTCATGAAGCATAATAATATTGAAATAATTTCGTTTGTAAATTATTTTTAAA

*A*  (1897) TAAATTTTACTTATAGTTTACAATTATTTCTGTATAATAATTTATACTCTCATGAAGCATAATAATATTGAAATAATTTCGTTTGTAAATTATATTTAAA

2001 2100

*a*  (1998) CTAAAAAATATTAATATCATTAGACTGATCATGAGTATTCAAATTTAAATTTAAAATATTTTAATTGTATGTGAATTTATAATAATTAATATGATTTCGT

*A*  (1997) CTAAAAAATATTAATATCATTAGACTGATCATGAGTATTCAAATTTAAATTTAAAATATTTTAATTGTATGTGAATTTATAATAATTAATATGATTTCGT

2101 2200

*a*  (2098) GTAGCTGGTATATTAATATGCTTATAGGCACTATATATGTGTGTACCTAGCTAGGTGATGAGAAGAGAAAAATTATAGTCATTTTTGGTGAGAGAGAAAA

*A*  (2097) GTAGCTGGTATATTAATATGCTTATAGGCACTATATATGTGTGTACCTAGCTAGGTGATGAGAAGAGAAAAATTATAGTCATTTTTGGTGAGAGAGAAAA

2201 2300

*a*  (2198) GGGGAGGATCTAGGTAGCTATTGTGAAATGCATGTGATCGAGTTTTAATGCAAAAAAAGCATGCAGCTGTAAAAATTTAATAACATACCAATTATGTTAA

*A*  (2197) GGGGAGGATCTAGGTAGCTATTGTGAAATGCATGTGATCGAGTTTTAATGCAAAAAAAGCATGCAGCTGTAAAAATTTAATAACATACCAATTATGTTAA

2301 2400

*a*  (2298) CATAGTTTTTGAAAACAAAATCTTTCCTTTCTTTTGTTAGAACAAAATTGATGACTGTTAAATCATTTTCTCATAAAAGAGCTTTGGCTTGAAAGTCATG

*A*  (2297) CATAGTTTTTGAAAACAAAATCTTTCCTTTCTTTTGTTAGAACAAAATTGATGACTGTTAAATCATTTTCTCATAAAAGAGCTTTGGCTTGAAAGTCATG

2401 2500

*a*  (2398) AATTTATACATTGTTTTCCCTCCGGTGGGATATGTATTTTGGTGAAGCATGCAGACTGTCACAGAGGCCCCTTGTGAGTTTGCTTAAATGCAACATCATC

*A*  (2397) AATTTATACATTGTTTTCCCTCCGGTGGGATATGTATTTTGGTGAAGCATGCAGACTGTCACAGAGGCCCCTTGTGAGTTTGCTTAAATGCAACATCATC

2501 2600

*a*  (2498) ACCTTCCAATTTATGAACTCTCACCAACAAAATAAAACATATTCAACATTTCATCTTAAGTTTTCGCTTACTAAGATACAATTAATCACCGTTTAACTGT

*A*  (2497) ACCTTCCAATTTATGAACTCTCACCAACAAAATAAAACATATTCAACATTTCATCTTAAGTTTTCGCTTACTAAGATACAATTAATCACCGTTTAACTGT

2601 2700

*a*  (2598) CAACTGTCGCAAAACATCGCTATTGTTTAGCTGTCACAAATATTTTATGAAATCTTATTTTGTCACAGTGTAATAGACATATATTTTATCACATTATTTA

*A*  (2597) CAACTGTCGCAAAACATCGCTATTGTTTAACTGTCACAGATATTTTATGAAATCTTATTCTGTCATAGTATAATAGACATATATTTTATCACATTATTTA

2701 2800

*a*  (2698) ACTATATTTTTTAATCTTACGCGGTATCTCGACTTGCACGCACTCATAATTAATAAAGTTTTCCATACAATATATATTCACGTATGGCTAGTAACAAATA

*A*  (2697) ACTATATTTTTGAATCTTACGCGGTATATCGACTTGCACGCACTCATAATTAATAAAGTTTTCCATACAATATATATTCACGTATGGCTAGTAACAAATA

2801 2900

*a*  (2798) GGCCAAAAAGGTAGCTAAAAACTTTATTACTTGATTCTATACTAGATACAATATGTTTTTGAGAGTAAAGTAAATGAAAAAGTACAATATTGAAAATGAG

*A*  (2797) GGCCAAAAAGGTAGCTAAAAACTTTATTACTTGATTCTATACTAGATACAATATGTTTTTGAGAGTAAAGTAAATGAAAAAGTACAATATTGAAAATGAG

2901 3000

*a*  (2898) AGTTTGTGTTAGATTATACTTGAGGAATATATATTCAGAGTATTCTATCGAATGAGAGTTTTTTTTTTTAATGTTGGTGAAGTATTTAGGTGCACATTAT

*A*  (2897) AGTTTGTGTTAGATTATACTTGAGGAATATATATTCAGAGTATTCTATCGAATGAGAGTTTTTTTTTT-AATGTTGGTGAAGTATTTAGGTGCACATTAT

3001 3100

*a*  (2998) TATATGTTTGAATAGGACATTTAGTTACTTACTTCAAGAAATCAGACAAATATGAATGCATTTTCAGACTTCAAAATAAATAACTTAGCTGTAAAAAAAT

*A*  (2996) TATATGTTTGAATAGGACATTTAGTTACTTACTTCAAGAAATCAGACAAATATGAATGCATTTTCAGACTTCAAAATAAATAACTTAGCTGTAAAAAAAT

3101 3200

*a*  (3098) CCACACTATTTTTACTACCATTCTACATTTAGAACTTTTCAACATCACATTTCACTCTCTTTGTCTCTATCAAAGTTTAACTTTTCATACATCATTAGTA

*A*  (3096) CCACACTATTTTTACTACCATTCTACATTTAGAACTTTTCAACATCACATTTCACTCTCTTTGTCTCTATCAAAGTTTAACTTTTCATACATCATTAGTA

3201 3300

*a*  (3198) TTTGGATGATACCTGTTTTTATCATAGCTTCATATAGTTTCTCCTATATTGCTAATAATTCATTTTGCAACAACATCAAAATGATAACATCAATTGGTCA

*A*  (3196) TTTGGATGATACCTGTTTTTATCATAGCTTCATATAGTTTCTCCTATATTGCTAATAATTCATTTTGCAACAACATCAAAATGATAACATCAATTGGTCA

3301 3400

*a*  (3298) CTGTATATGCATATTCCTTACAGTGAGACTAATTCGTTCTAGATTATTATAGAATAAGAGGTAGATATATAGATAGGATATAAATGTAGGTTATTCTATC

*A*  (3296) CTGTATATGCATATTCCTTACAGTGAGACTAATTCGTTCTAGATTATTATAGAATAAGAGGTAGATATATAGATAGGATATAAATGTAGGTTATTCTATC

3401 3500

*a*  (3398) AATAATAATATATACTTAGTTACATTACATATATACCAAAGCTAGCTATATGTATATATATTTGATTTGATGACAAAGAAAAAGCATATTATAGTGGAGA

*A*  (3396) AATAATAATATATACTTAGTTACATTACATATATACCAAAGCTAGCTATATGTATATATATTTGATTTGATGACAAAGAAAAAGCATATTATAGTGGAGA

3501 3600

*a*  (3498) GGGGTATAATAAATTGATGTTTTTCTTTTTCTTTTTTGAAAAACTTGTATTATTATAACTTTTGAAATTATTATTTTTGGTAGAGTAAAGAAATAAGTTA

*A*  (3496) GGGGTATAATAAATTGATGTTTTTCTTTTTCTTTTTTGAAAAACTTGTATTATTATAACTTTTGAAATTATTATTTTTGGTAGAGTAAAGAAATAAGTTA

3601 3700

*a*  (3598) TTTCATATGTTTATAAGGTTGCCAGGAAAGGCATATGCTAGGAGGCAACATGTATGGCTCACGGGTGCAAATGAGGTGGATAGCAAAACATTTTCAAGAG

*A*  (3596) TTTCATATGTTTATAAGGTTGCCAGGAAAGGCATATGCTAGGAGGCAACATGTATGGCTCACGGGAGCAAATGAGGTGGATAGCAAAACATTTTCAAGAG

Exon 3 GTTGCCAGGAAAGGCATATGCTAGGAGGCAACATGTATGGCTCACGGGAGCAAATGAGGTGGATAGCAAAACATTTTCAAGAG

3701 3800

*a*  (3698) CTATTTTAGCCAAGGTAATCTCCGATTAACATATAATTAATATTAATTATTTTGTCCGATTAAACCAACTTAGTTCGTAAAATAGTAAGTATCTGTCATT

*A*  (3696) CTATTTTAGCCAAGGTAATCTCCGATTAACATATAATTAATATTAATTATTTTGTCCGATTAAACCAATTTAGTTCGTAAAATAGTAAGTATCTGTCATT

Exon 3 CTATTTTAGCCAAG

3801 3900

*a*  (3798) AGATTATTAAAAAAATATTAATTACTTGTATTAAGAGGATATTTTATTATTCATTTTTTTTATGAAGTTGAGAAATTGAAAAACACAACAACTTTATCAA

*A*  (3796) AGATTATTAAAAAAATATTAATTACTTGTATTAAGAGGATATTTTATTATTGATTTTTTTTATGAAGTTGAGAAATTGAAAAACACAACAACTTTATCAA

3901 4000

*a*  (3898) TTAAGCTACTTACTTTGGCAAGTTTCCATTTAAAAAGAAAGTTTTTAGAAGTTGCTTTCATACTATGGATTTATTGATCATTATGAAGCTTAATTTACTA

*A*  (3896) TTAAGCTACTTACTTTGGCAAGTTTCCATTTAAAAAGAAAGTTCTTAGAAGTTGCTTTCATACTATGGATTTATTGATCATTATGAAGCTTAATTTACTA

4001 4100

*a*  (3998) ACAAACTTATCTTTCCCATGGCTTCTCAATGATGACTACTTTTTCTACTGGCTATAGAGTGCTAATATACAGGTAACAAGATTTTCAACTAGTTTTATCC

*A*  (3996) ACAAACTTATCTTTCCCATGGCTTCTCAATGATGACTACTTTTTCTACTGGCTATAGAGTGCTAATATACAGGTAACATGATTTTCAACTAGTTTTATCC

Exon 4 AGTGCTAATATACAG

4101 4200

*a*  (4098) ATTTTTAACTTTAATTTTGTACTCCATTAAATTCTAATGAAAATCATAAAATAAACAAAATAGTACTTTTTTTATCTACAGACTGTGGTATGCATTCCTG

*A*  (4096) ATTTTTAACTTTAATTTTGTACTCCATTAAATTCTAATGAAAATCATAAAATAAACAAAATAGTACTTTTTTTATCTACAGACTGTGGTATGCATTCCTG

Exon 5 ACTGTGGTATGCATTCCTG

4201 4300

*a*  (4198) TGTTGGATGGTGTCGTTGAGATTGGCACAACTGATAAGGTAACATACTTTCTATTAATAGAATAATTTTGTGGATTGAAATGCAATAAATAAATATACAT

*A*  (4196) TGTTGGATGGTGTCGTTGAGATTGGCACAACTGATAAGGTAACATACTTTCTATTAATAGAATAATTTTGTGGATTGAAATGCAATAAATAAATATACAT

Exon 5 TGTTGGATGGTGTCGTTGAGATTGGCACAACTGATAAG

4301 4400

*a*  (4298) AAACTTTGAAAAATAATTAATTTTTTATTGAAATTTCAAAGTAATAAATAATATAACACAAACAAATATTTAAAATGTGATGTTTAATAGTTTAATAATC

*A*  (4296) AAACTTTGAAAAATAATTAATTTTTTATTGAAATTTCAAAATAATAAATAATATAACACAAACAAATATTTAAAAGGTGATGTTTAATAGTTTAATAATC

4401 4500

*a*  (4398) ACATTAAGACGGTTCAGTTCAGTTGATAGTTGTGTAGAGACATCAGACATATGAACCGTTTAATTATTATTTTTATGTTT--TTTTTTTTAAGGTTCAAG

*A*  (4396) ACATTAAGATAGTTC-------------GTTGTGTAGAGACCTCAGACATATGAACCGTTTAATTATTATTTTTATGTTTATTTTTTTTTAAGATTCAAG

Exon 6 ATTCAAG

4501 4600

*a*  (4496) AAGATCTTAATTTCATCAAACACGTGAGGAGTTTCTTCATAGACCACCACTCTTTGCCACCAAAGCCAGCACTCTCTGAACACTCAACCTCCAATCCGAC

*A*  (4483) AAGATCTTAATTTCATCAAACACGTGAGGAGTTTCTTCATAGACCACCACTCTTTGCCACCAAAGCCAGCACTCTCAGAACACTCAACCTCCAATCCGAC

Exon 6 AAGATCTTAATTTCATCAAACACGTGAGGAGTTTCTTCATAGACCACCACTCTTTGCCACCAAAGCCAGCACTCTCAGAACACTCAACCTCCAATCCGAC

4601 4700

*a*  (4596) TTACTCAACCGATCACATTCCTGCCATTATGTACACAGTGGCAGACCCAGCCTCCACGACAATTCCCAATCAAGACGATATGGATGAAGATGAGGAAGAG

*A*  (4583) TTACTCAACCGATCACATTCCTGCCATTATGTACACAGTGGCAGACCCAGCCTCCACGGCAATTCCCAATCAAGACGATATGGATGAAGATGAGGAAGAG

Exon 6 TTACTCAACCGATCACATTCCTGCCATTATGTACACAGTGGCAGACCCAGCCTCCACGGCAATTCCCAATCAAGACGATATGGATGAAGATGAGGAAGAG

4701 4800

*a*  (4696) GATGACGAAGATGACGAAGTTGAATCTGGATCTGAAGATGAAACCAACCAAGGTCATAACCAACACGCAACCTCTATAATAGAGGCTGCGGAACCGAGTG

*A*  (4683) GATGATGAAGATGACGAAGTTGAATCTGGATCCGAAGATGAAACCAATCAAGGTCATAACCAACACGCAACCTCTATAATAGAGGCTGCGGAACCGAGTG

Exon 6 GATGATGAAGATGACGAAGTTGAATCTGGATCCGAAGATGAAACCAATCAAGGTCATAACCAACACGCAACCTCTATAATAGAGGCTGCGGAACCGAGTG

4801 4900

*a*  (4796) AACTCATGCAAATTGAAATGCCCGATGATATTCGGATCGGGTCACCCAACGATGGGTCGAATAATTTGGACTCGGATTTTCATTTGTTGGCCGTTAGTAA

*A*  (4783) AACTCATGCAAATTGAAATGCCCGATGATATTCGGATCGGGTCACCCAACGACGGGTCAAATAATTTAGACTCGGATTTTCATTTGTTGGCCGTTAGTAA

Exon 6 AACTCATGCAAATTGAAATGCCCGATGATATTCGGATCGGGTCACCCAACGACGGGTCAAATAATTTAGACTCGGATTTTCATTTGTTGGCCGTTAGTAA

4901 5000

*a*  (4896) TCAAGGAAACCCATCAAGACAAATTGACTCATATACAACCGAGAGATGGGGTCCAATCGAAGAACCTCTCGATGATTCACTACAAGTTCAATTATCATCT

*A*  (4883) TCAAGGAAACCCATCAAGACAAATTGACTCATATACAACTGAGAGATGGGGTCCAATCGAAGAACCTCTCGATGATTCACTACAAATTCAATTATCATCT

Exon 6 TCAAGGAAACCCATCAAGACAAATTGACTCATATACAACTGAGAGATGGGGTCCAATCGAAGAACCTCTCGATGATTCACTACAAATTCAATTATCATCT

5001 5100

*

*a*  (4996) TCAGATAAATCGGTAATTAACAAATTTAAATTTCAAATATGTTTTGAATCCTTGTATTTAGGTTTGTCCTAACTCCTAGCCCTAACGAACTTCACTAAAT

*A*  (4983) TCAGGTAAATCGGTAATTAACAAATTTAAATTTCAAATATGTTTTGATTCCTTGTATTTAGGTTTGTCCTAACTCCTAACCCTAACAAACTTCCCTAAAT

Exon 6 TCAG

5101 5200

*a*  (5096) TTTATGTGCCAAATAAATTTATAAAAAAAAACATAAAGACATGATGCGGTGGACTCACAAACTGAAGCATACAAGTTATAAAGTTTTTGAAAGCGAAATA

*A*  (5083) TTTATGTGCCAAGTAAATTTATAAAAAAAAACATAAAGACATGATGCGGTGGACTCACAAACTGAAGCATACAAGTTATAAAGTTTTTGAAAGCGAAATA

5201 5300

*a*  (5196) ACGTCTTAAATTTTTTTAAATATTATAATCGAATATTTCTACGACTAACAACTGAACTGAATTAACGGAATAATTAATCTATCATATATTGATTTAAGCG

*A*  (5183) ACTTCTTTAATTTTTTTAAATATTATAATCGAATATTTCTACGACTAACAACTGAACTGAATTAACGGAATAATTAATCTATCATATATTGATTTAAGTG

5301 5400

*a*  (5296) AATTGGACCACATCAGGGTACATAGAGTTTGGGGGGCTAGCTAGTGATTAGATCCAAAGTTTTTTTCACCACATATCAATGTGGTCCATCCTAATTAAGT

*A*  (5283) AATTGGACCACATCAGGGTACATAGAGTTTGGGGGGCTAGCTAGTGATTAGATCCAAAGTTTTTCTCACCACATATCAATGTGGTCCATCCTAATTAAGT

5401 5500

*a*  (5396) CCATTGTCTACCCCATTTTTGGTGGGGTCCCCAATCTATGAAAAATCGAATTCATTTTACTGCTCACTCACCAAAAATGTTCAACAGCCCCACGGTACAT

*A*  (5383) CCATTGTCTACCCCATTTTTGGTGGGGTCCCCAATCTATGAAAAATCGAATTCATTTTACTGCTCACTCACCAAAAATGTTCAACAGCCCCACGGTACAT

5501 5600

*a*  (5496) TATACAAGTAATAGTAATACAACAACATACATTAAACTTATATAGTAGTAATAAAACTTCTACTTTACTTCAATAATTATAAAAGTGTCGAGTGTAAATT

*A*  (5483) TATACAAGTAATAGTAATACAACAACATACATTAAACTTATATAGTAGTAATAAAACTTCTACTTTACTTCAATAATTATAAAAGTGTCGAGTGTAAATT

5601 5700

*a*  (5596) TGGATTGTCTTCAACTAGGTACTATTTTGTGGGCCTTAAAACAGTAGTCGGAGTGTGTTAAGAAGCAGTGTCTGGTGAATGGTGATTAAGAAAAAAAAAT

*A*  (5583) TGGATTGTCTTCAACTAGGTACTATTTTGTGGGCCTTAAAACAGTAGTCGGAGTGTGTTAAGAAGCAGTGTCTGGTGAATGGTGATTAAGAAAAAAAAAT

5701 5800

*a*  (5696) TGCAGATAGCTACAGAATCTGACAATGGCAGGTGCTAACCGGGGAAAGATAATTTGATCATCTTTGAAAGCTATGGCTCAGAATTTTTCATGTTTGCAAC

*A*  (5683) TGCAGATAGCTACAGAATCTGACAATGGCAGGTGCTAACCGGGGAAAGATAATTTGATCATCTTTGAAAGCTATGGCTCAGAATTTTTCATGTTTGCAAC

5801 5900

*a*  (5796) CAAAGAGGAAAACTAGAAAAAACATTAATTGCATGCATACGGAATTTGTCTGAACCAAAAAATACATTTTGAAATTAAGACTACAATTCGTACGTACTAG

*A*  (5783) CAAAGAGGAAAACTAGAAAAAACATTAATTGCATGCATACGGAATTTGTCTGAACCAAAAAATACATTTTGAAATTAAGACTACAATTCGTACGTACTAG

5901 6000

*a*  (5896) TTTATTTATGTTATCTTCACTTCTAAAAGAACCAGCAATTTGGAGGAGTATATACTCTTTTTTTCTTGATGGAAAACCAACACACATGAAATTTGAATGG

*A*  (5883) TTTATTTATGTTATCTTCACTTCTAAAAGAACCAGCAATTTGGAGGAGTATATACTCTTTTTTTCTTGATGGAAAACCAACACACATGAAATTTGAATGG

6001 6100

*a*  (5996) TAATGGTAGGTTCATAAACATGATTTCAAATATGAACTGTCATTTTCAACATCCTTTGAGACATTATAAGTTCAACTAGTTTTAAATTGTTCATATATAC

*A*  (5983) TAATGGTAGGTTCATAAACATGATTTCAAATATGAACTGTCATTTTCAACATCCTTTGAGACATTATAAGTTCAACAAGTTTTAAATTGTTCATATATAC

6101 6200

*a*  (6096) AAAAAAGTTTAATAGATATCCACTTCATTAAAGTTATTTGGTAAATTTATTGAATAACAAATATATATGATTTTTATACTCTATAGATTAAATACATCAT

*A*  (6083) AAAAAAGTTTAATAGATATCCACTTCATTAAAGTTATTTGGTAAATTTATTGAATAACAAATATATATGATTTTTATACTCTATAGATTAAATACATCAT

6201 6300

*a*  (6196) TTATTTATCTTAGTTGTTAATCCAAAACCATTTCTCTCTAAAATGTTACTCTCTCTTAATAAGTGTCATCTTTCAAATTTTTATTTGTCTAAAATTATTT

*A*  (6183) TTATTTATCTTAGTTGTTAATCCAAAACCATTTCTCTCTAAAATGTTAGTCTCTCTCAATAAGTGTCATCTTTCAAATTTTTATTTGTCTAAAATTAGTT

6301 6400

*a*  (6296) CTCCTTTTAGAATACCAATGTGATATTTATTAGTTTTTTCCCACTAACTTAACTTTATTTATTGTATTTTAATTTATGTAACTATTCTACTACCTATTAT

*A*  (6283) GTCCTTTTAGAATACCAATGTGATATTTATTAGTTTTTTTCCATTAACTTAACTTTATTTATTGTATTTTAATTTATGTAACTATTCTACTACCTATTAT

6401 6500

*a*  (6396) TAATAAGGTTATTTTAGTAAATGATACGATTTTTATCATTGAAATCAACACAATTAATCATTATTTTTAAAATGTGAAAATCTCAAAGAAAATACATATT

*A*  (6383) TAATAAGGTTATTTTAGTAAATGATACAATTTTTATCATTGAAATCAACACAATTAATCATTATTTTTAAAATATGAAAATCTCAAAGAAAATACCTATC

6501 6600

*a*  (6496) GTAAGATTGATGAAGTAAGTTATTAGTTATTA---GTAGATTAACAACTAACTAGCTAAATTAGTTAATTAGATGGGTTGGTTACTTAACCAACAAGATA

*A*  (6483) GTAAGATTGATGAAGTAAGTTATTAGTTATTATTAGTAGATTAATAACTAACTAACTAAATTAATTAATTAGATGGGTTGGTTACTTAACCAAGAAGATA

6601 6700

*a*  (6593) TATAAAGTTATTGTATAGTTCAGTTTTCAATTACTTCAATAATTCGTTCACTTTTAGCACAACTCCATCTTCTTTATTGTGTATAAATTGTTGGTG----

*A*  (6583) TATAAAGTTATTGTATAGTTAAGTTTTCAATTACTTCAATAATTCGTTCACTTTTAGCACAACTCCATCTTCTTTATTGTGTATAAATTGTTGCTTGCAC

6701 6800

*a*  (6689) --------ACAACAGAGTTGTGCTAATAATTGGCATTGAGAGCATGGTTTTATCTTAGAGAAACGCGAGAGAATTGCGAGGGTCATACCATTTGGAAACA

*A*  (6683) CACAAGAAACAATAGAGTTGTGCTAATAATTGGGATTGAGAGCATGGTTTTATCTTAGAGAAACGCGAGAGAATTGCGAGTGTCATACCATCTGGAAACA

6801 6900

*a*  (6781) AAGAGTGAGGGTAAGTTTTATCTTCTTGATCGGCAAGAATGAACATAAGTGGTGGCTTTTCGTCAAACATATTGATTCTAGATGGAGAAAATTGAAAAAG

*A*  (6783) AATAGTGAGGGTAAGTTTTATCTTCTTGATCGGCAAGAATGAACATAAATGGTGGCTTTTCGTCAAACATATTGATTCTAGATGGAGAAAATTGAAAAAG

6901 7000

*a*  (6881) ATGAAGTGATTTGATGAAACCGGAAGTATAAACCACAAAAGTGCAAAGAGTCACATTCAAACAAAGAAAAGTTGAAGAATAATGATCGTTAAGTTTTGTT

*A*  (6883) ATGAAGTGATTTGATGAAACCGGAAGTATAAACCACAAAAGTGCAAAGAGTCACATTCAAACAAAGAAAAGTTGAAGAATAATGATCGTAAAGTTTTGTT

7001 7100

*a*  (6981) CTACATTCAAGAATATGTGGATACTCATCACCAGGCCCGACCATGTGCATGTGCAACATGTGCTGCAGCACAGGGCCTCACATTTTAGAAGGCTCCAAAA

*A*  (6983) CTACATTCAAGAATATGTGGATACTCATCACAAGGCTCGGCCCTATGCATGTGTAACATGTGCTGCAGCATAGGGCCTCACATTTTAAAAGGCTCCAAAA

7101 7200

*a*  (7081) TTTAAAAAATTTAATTTTGTCTTTATAAATATTAATAAAAATAAATAAAATATTATTTAAAAAATTCAATAATTAAAAAAAATTATGATAAAAATTTAAT

*A*  (7083) TTTGAAAATTTTAATTTT-TCTTTATAAATATTAGTAAAAATAAATAAAATATTATTAAAAAAATTCAATAATTAAAAAAAATTATGATAAAAATTTAAT

7201 7300

*a*  (7181) ACATACAAAAATTAAGATTGATCAAAACTCAAAATAATTATAATTAAATTTATTTTTAATTAATACATAATTATATTTTTGTAATATATTTTTAATTATT

*A*  (7182) ACATACAAAAATTAAGATTGACCAAAACTCAAAATAATTATAATTAAATTTATTTTTAATTAATACATAATTATATTTTTGATATATATTTTTAATTATT

7301 7400

*a*  (7281) ATAATTGTA-TTTTTTAAAAAATTTGAACCTATTTTTAAAATTAGAACAGGGCCTCCGATATGATTGGATCGGCCCTGCTCATCACTTTTAAAAGATCTC

*A*  (7282) ATAATTGTATTTTTTTAAAAA-TTTGAACCTATTTTTAAAATTAGAACGAGACCTCCGATATGATTGGGTCGACCCTGTTCATCACTTTTAAAAGATCTC

7401 7500

*a*  (7380) TAAGGTAAAAAGATCAAAAGAGACATGTGATATTACAGAGGAGTACCAAGATGGCGGGGAAAACGTGAAGCTTCAATCTTTAAGAATGAAGTACCAATTG

*A*  (7382) TAAGGTAAAAAGATCAAAAGAGACATGCGATATTATTGAGGAGTACCAAGATGGCAGGGGAAACGTGAAGCTTCAATCTTTAAGAATGAAGTACCAATTT

7501 7600

*a*  (7480) ATGCGGATGGAAGAAGATCAAAAGATGAGTGCTCACCTCTTTAAATTGATTGCAGTGGTGAATCAAGTGAAGACTTATGGTGAGACTATTGTTGATCAAC

*A*  (7482) ATGCGGATGGAAGAAGATCAGAAGATGAGTGATCACCTCTTTAAATTGATTGCAGTGGTGAATCAAGTGAAGACTTATGGTGAGACTATTGTTGATCAAC

7601 7700

*a*  (7580) AAGTGGTTAAGAAAGTAATGAGATCCCTGACTTCAAGATTTTATTTTGTAGTGGTGGTAATTCAAGAACCAAAAGATTTGATGACCATGAAAATCGAGGA

*A*  (7582) AAGTGGTTAAGAAAGTAATGAGATCCCTGACTTCAAGATTTTATTTTGTAGTGGTGGTAATTCAAGAACCAAAAGATTTGATGACCATGAAAATCGAGGA

7701 7800

*a*  (7680) GCTGTTGAATACTTTGGAGACTCATGAACATGTGGTGATTGATAAAGAAGCATAGATTTCGGTGCAACAAGTCTTGCAAGCTCATGTGATGAAGAAATAT

*A*  (7682) GCTGTTGAATACTTTGGAGACTCATGGACATGTGGTGATTGATAGAGAAACATAGATTTCGGTGCAACAAGTCTTGCAAGCTCATGTGATGAAGAAATAT

7801 7900

*a*  (7780) GGTCAATAAAGGAAGTTAAAGAGAAAAAAAAAATAGAAAGCTTCAATAGTGGAAGTTAGTCTAATAGTAGCAAATCTAAGGTTGATGACAAAGTTGGATC

*A*  (7782) GGTCAATAAAGGAAGTTAA------ATAAAAAATAGAAAGCTTTAATAGTGGAAGTTAGTCTAATAGTAGCAAATCTAAGGTTGATGACAAAGTTGGATC

7901 8000

*a*  (7880) TGACAAGAGAGGGGAAGGTTCTGGAACCAACAAATTAAAGATTAAGGAATTTGATAAAAATAAAGGAAAGTTTTGTGCTACAGTTATGAGAATTATGAAT

*A*  (7876) TGACAAGAGAGGGGAAGGTTCTAGAACTAACAAATTAAAGATGAAGGAATTTGATAAAAATAAAGGAAAGTTTTGTGCTATAATTATGAGAATTATGAAT

8001 8100

*a*  (7980) ATTTTGTTGATGAATGTTGGCACTAGAAATAAGGCAATAAAAATAGGTGTACATAGCTCTTGATGATGACTATGATTTAGAATCAATTTTTCTAATAGCC

*A*  (7976) ATTTTGTTGATGAATGTTGGCACTAGAAATAAGGCAATAAAAATAGGTGTACATAGCTCTTGATGATGACTATGATTTAGAATCAATTTTTCTAATAGCC

8101 8200

*a*  (8080) ATAGAAGGAAACCTATGTATGAGTTTTGATTCTGGGTGTTAAATCACATGAGTGGAATAAATAATGTCTCACAAAATTTGATACAAATAACGGTTGCCTT

*A*  (8076) ATAGAAGGAAACCTATGTATGAGTTTTGATTCTGGGTGTTAAATCACATGAGTGGAACAAATAATGTCTCACAAAGTTTGATACAAATAACGGTTGCCTT

8201 8300

*a*  (8180) TCACAAGAAGCCGTGGATAACAATGTCATCATCATAAAGGATGGCATGAATGTACATGTTTAAAAGGTAATTTGTGTACATGGTATATGTTAAGAAACAA

*A*  (8176) TCACAAGAAGCCGTGGACAACAATGTCATCATCATAAAGGATGACATGAATGTACACGTTTAAAAGGTAATTTATGTACATGGTATATGTTAAGAAACAA

8301 8400

*a*  (8280) GTGTGAGTTGAAAGTCACACATTACTTAGAAAAATAGAGGTTGAGAACTTTATAAGTGAGAGGACCTATATACTTAATGCCTTGAGGGTTTTGATGAATA

*A*  (8276) GTGTGAGTTGAAAGTCACACATTACTTAGAAAAATAGAGGTTGAGAACTTTATAAGTGAGAGGACCTATATACTTAATGCCTTGAGGGTTTTGATGAATA

8401 8500

*a*  (8380) TGTGATGTCTCTCTCACTTGAGTGTTTATCTTTATCCAATATGGTTGTTCCCCTCACTATCCAATAATGATATTAAAGTTGATGGTTCCAGTAAGAGAAT

*A*  (8376) TGTGATCTCTCTCTCACTTGTGTGTTTCTCTTTATCCAATATGGTTGTTCCCCTTACTATCCAATAATGGTATTAAAGTTGATGGTTCAAGTAAGAGAAT

8501 8600

*a*  (8480) GACCCCTTATATCAAAAGTCTTCCTAACATGGTGGTGGTTGGCGACGTGTCCTGTTGAAGTGCACGTGACGTGGTATGGGTCTCAACGTTTGTACAAGTG

*A*  (8476) GGCCCCTTATATCAAAAGTCTTCCTAATATGGTGGTGGTTGGCGACGTGTCCTGTTGAAGTGCACGTGACGTGGTATGGGTCTCAACGGTTGTACAAGTG

8601 8700

*a*  (8580) TATGTACTTACACTTTAGAGAGCAATTATTAAGATACAAGCGTGAGTTACAAGTTTAGTATGATGTGCAACTTGATGAGTACTGAGAAACTAGTCAAAAA

*A*  (8576) TATGTACTTACACTTTAGAGAGCAATTATTAAGATACAAGCGTGAGTTACAAGTTTAGTATGATGTACAACTTGATGAGTACTGAGAAACTAGTCGAAAA

8701 8800

*a*  (8680) GGTATTTTTAATTACCATGAATAGTAATTCACTACAATTGTTTGATCCTCACTAGAAGTTGGTACTAAAGTAGACCTTCTCAAAGAATATAACATTCAAA

*A*  (8676) TGTATTTTTAATTACCATGAATAATAATTCACTACAATTGTTTGATCCTCACAAGAAGCCAGTACTAAAGTAGACCTTCTCAAAGAATATGGCATTCAAA

8801 8900

*a*  (8780) CGTAGCATTCTAACTATAAAGGAAGTATGTTTGTCAATAACTACTCATTATGAACTTGTGATTTGACATATGAGGTATGACCACTTAATTTTTAGAAGCC

*A*  (8776) CATAGCATTCTAACTATAAAGGAAGTATGTTTGTCAATAACTACTCATTATAAACTTGTGATTTGACATATGAGGTATGGCCACTTAATTTTTAGAAGCC

8901 9000

*a*  (8880) TAATTCAATTAAGTTCTAAGATTTTAGAATATTACCTACCTAAATTCAATTCAAATATATTGTCTTATGAAGCTGGTTTGAGAGGCGAACATAGCATATT

*A*  (8876) TAATCCAATTAAGTTCTAAGATTTTAGAATATTACCTACCTAAATTGAATTCAAATATATTGTCTTATGAAGCTGGTTTGAGAGGCGAACATAGCATATT

9001 9100

*a*  (8980) ATCCTTTATAATAGACATGCCTAACAAAACAAATAATGCACTAAAGATTGTGCATTCTAATATATGCGGATATTTTGAGGTATCACCATAACGCGGGATA

*A*  (8976) ATCCTTTATAATAGACATGCCTAACAGAACAAATAATGCACTAAAGATTGTGCATTCTAATATATGCGGATCTTTTGAGGTATCACCATAACGTGGGATA

9101 9200

*a*  (9080) AATTATTTAATAACTTTTGTTAATGAGCATACAAGAATGATGTGGCTATATACCATCAAGCTCAAAAGAAAAGCTCTTGAAGTGTTCAAGAAATTCAACA

*A*  (9076) AATTATTTAATAACTTT-GTTAATGAGCATACAAGAATGACGTGGCTATATACCATCAAGCTCAAAAGAGAAGCTCTTGAAGTGTTCAAGAAATTCAGCA

9201 9300

*a*  (9180) CATTAATTGAGAAGGAGAGTGGGAAGTCAATAAACGTTTTAAGGACTGTGGTGGTGGAGAGTATAACTTAAAAAAATTTGAATCATTTTACATTAGTGAA

*A*  (9175) CATTAATTGAGAAGGAGAGTGAAAAGTCAATAAACGTTTTGAGGACTGTGGTGGTGGAGAGTATAACTTAAAAGAATTTGAATCATTTTACATTAGTGAA

9301 9400

*a*  (9280) TGCATAATACATAATGTCACATCACCTTATGCTCCTC-------TGGTCTTTCAGAAAGAAGAAATATGGTCATACTTGACATGGTCAAGAGTATTCTAA

*A*  (9275) TGCATAATACATAATGTCACATCACCTTATGCTCCTCAACTTAATGGTCTTGCAGAAAGAAGAAACATGGTTATACTTGACATGGCTAAGAGTATTCTAA

9401 9500

*a*  (9373) AACACAAGAGCATGCCTTGTAATGGGGTGAAGCAATCAATGCAACTACTTATATACTAAACAAATGCTCCACAAAAAAGATGAGCATGAAAGCGCCAAAA

*A*  (9375) AACACAAGAACATGTCTCATAATGGGGTGAAGCAATCAATGCAACTATTTATATACTAAACAAATGCTCCACAAAAAAGATAGGCATAAAAGCGCTCAAA

9501 9600

*a*  (9473) GGTGCTTGGAGTGAAAGAAAATCAAATGTGAAGCATTTTAAAGTATTTTGATCATTAGGTTTCAAGCACATATCTGATCAAAGAAGAAGTAATTTGGATG

*A*  (9475) GACGCTTGGAGTGAAAGAAAATCAAATGTGAAGAATTTTAAAGTATTTAGATCATTAGGTTTCAAGCACATACCTTATCAAAGAAGAAGTAACTTTGATG

9601 9700

*a*  (9573) ACGAAAATGAAAGCATGGTATTCATAAGATATCATTAAATATGATTTTATAAGATATACGATCATGTGATTCAAAAT-TGCACATCATTAGATATGTGTT

*A*  (9575) ACCAAAATGAAAGCATGGTATTCATAAGATATCATTAAACATGATTCTATAAGATATACAATCATGTGATTCAAAAGGTGCACATCATTAGATATGTGAT

9701 9800

*a*  (9672) TTTTCAATAAATCACATGCCTGAAAATGGCTAGACACACTGAAATAAAGAAGTAATTTCAATAATGTCTTTGTTGAACCTAATAAGAGTGAATGGTCATA

*A*  (9675) TTTTCAATGAATCACATGCCTGGAATTGGCTAGACACACTGAAATAAAGAAGTAATTTCAATAATGTCTTTATTGAACCTAATAAGAGTGAATGGTCATG

9801 9900

*a*  (9772) TGAACAAATTATTGTTGGAGACACTAGAGTAGCCAATGATAGACCAATAGAAGCTCAAACACATGTGAGGCCTTAGAGAACAAGGAAATTTCCTAGTAGA

*A*  (9775) TGAATAAATTGTTGTTGGAGACACTAGAGTAGCTAATGATAGACCAATAGAAGCTCAAACACGTGTGAGGCCTTAGAGAACAAGGAAAGTTCCTAGTAGA

9901 10000

*a*  (9872) GTTACAAATTGTGAGTTACACCCTGGTTGTGAAGTCAACAATAAAGGTGAACTCGTTCATTTTTCTTTGTTAGTTGATATTGTTTCAATCGGCTATGACA

*A*  (9875) GTTACAAATTGTGAGTTACGCCCTAGTTGTGAAGTCAACAATAAAGGTGAATTCGTTCATTTTTCTTTGTTAGTTGATATTGTTTCAATCGACTATGACA

10001 10100

*a*  (9972) AGGCAATAAAAAAAGTATGGAAAAAGATTGTGATTGAGGAACTTAAGTCAATTTAAAAGAATCATGTTGGGGAGTTGGTTACTCTGCTCGAAAAGAAGAA

*A*  (9975) AGGCAATAAAAAAAGTATGGAAGAAGACTGTGATTGAGGAACTTAAGTCAATTTAAAAGAATCATGTTAGGGAGTTGGTTACTCTGCTTGAAAAGAAGAA

10101 10200

*a*  (10072) AGAATTTTTAAGGTTAAAATGAACCTAGCTGATACAATTTTAAAGCATAAAGCAAGGTTGGTAGTTAGAGACTTCTTACAGAAGTATGGTATTTACTATA

*A*  (10075) AGAATTTTTAAAGTTAAAATGAACCTAGCTGATACAATTTTAAAGCATAAAGCAAGGTTGATAGTTAGAGGCTTCTTACAGAAGTATGGTATTTACTATA

10201 10300

*a*  (10172) ATGAAGTGTTTTCTCGTGTAATAAGACTTGGGACAACGAGACTAGTTACGAAAATAGCTAGTATCGAAAATTGGTCCCTTTACCACTTAAATGTCAAAGT

*A*  (10175) ATGAAGTGTTTTCTCGTGTGATAAGATTTGGGACAACGAGACTAGTTACGAAAATAGTTAGTATCGAGAATTGGTCCCTTTACCACTTAAATGTGAAAGT

10301 10400

*a*  (10272) CTGCATTCTTAAAAGGTCCATAAGAAGAAACAAATTTTGTCA-TCAACCACCAAATTTTTAGATAAAAGGAAAAGAGAGAACATAATGTACAATTTACCT

*A*  (10275) TTGCATTCTTAAAAAGTCCATAAGAAGAAACAATTTTTGTCACTCAACCACAAAATTTTTAGATAAAAGGAAAAGAGAGAACATAATGTACAATTTATCC

10401 10500

*a*  (10371) GAAGCTTTGTATAGATCGAAGCAAATCCTTAGAATTTGAAACAAAAGAATTGATGATTTTTTTATTCTACTGAGATTGAAAAGTGAACACTTAGTAAAGT

*A*  (10375) AAAGTTTTGTATAGATTGAAGCAAATCCTTAGAATTTGAAACAAAAGAATTGACAATTTTTTTATTCTACTGAGATTGAAAAGTGAACACTTAGTAAAGT

10501 10600

*a*  (10471) GTATGTGAAAAGTTCTAGAAAGTTCAAAGATTGTGATGATATTCTTATATACTCGTCACCCTTAACCATGCATAAGTTTTCATTTAAGTATTTTTGTAGA

*A*  (10475) GTATGTGAAAAGTTCTAGAAAGTTCAATGATTGTGATGATATTCTTATATACTCGTCACCCTTAACCATGCATAAGTTTTCATTTAAGTATTTTTGTAGA

10601 10700

*a*  (10571) TTTCAATCTTCATAAAATCAAACTTACTAAATTGTGTTTCTAAAATTTAGAATAATATTTTCTTTCATGATAAATTTTATATATAAATAATTTGTTTATT

*A*  (10575) TTTCAATCTTCATAAAATCAAACTTACTAAATTGTGTTTCTAAAATTTAGAATAATATTTTCTTTCATGATAAATTTTATTTATAAATAATTTGTTTATT

10701 10800

*a*  (10671) CATAAAAATATAATTGATTAGAAGACTTAACATACAAATATTATTCCATTTGTAGTAGAATCTCAGACATTGACATTTCTTTATTCTTTCTTTCTATGCA

*A*  (10675) CATAAAAATATAATT--------------ACATACAAATATTATTCCATTTGTAGTAGAATCTCAGACATTGACATTTCTTTATTCTTTCTTTCTATGCA

10801 10900

*a*  (10771) GTACTTCATCATCCATTAGAAGACTTAACACAAGAAGACACACACTACTCTCAAACAGTAACCACCATTCTCCAAAACCAATGGATCGATTCACCTTCCA

*A*  (10761) GTACTTCATCATCCATTAGAAGACTTAACACAAGAAGACACACACTACTCTCAAACAGTAACCACCATTCTCCAAAACCAATGGATCGATTCACCTTCCA

Exon 7 TACTTCATCATCCATTAGAAGACTTAACACAAGAAGACACACACTACTCTCAAACAGTAACCACCATTCTCCAAAACCAATGGATCGATTCACCTTCCA

10901 11000

*a*  (10871) TCAACTACATCAACTACTCCACCCAATCATCTTTCACCACCTGGACCAACCACCACTTCCACCCGCCGCCGCCGCCGGACCCTGCCACCTCCCAGTGGCT

*A*  (10861) TCAACTACATCAACTACTCCACCCAATCATCTTTCACCACCTGGACCAACCACCACTTCCACCCGCCGCCGCCGCCGGACCCTGCCACCTCCCAATGGCT

Exon 7 TCAACTACATCAACTACTCCACCCAATCATCTTTCACCACCTGGACCAACCACCACTTCCACCCGCCGCCGCCGCCGGACCCTGCCACCTCCCAATGGCT

11001 11100

*a*  (10971) CCTCAAATACATCCTCTTCACCGTCCCATACCTCCACACCAAGAACCACGACGAAACCTCTCCACAAACACGCGACACCGCCGGAGTCAACAGCAACGAT

*A*  (10961) CGTCAAATACATCCTCTTCACCGTCCCATACCTCCACACCAAGAACCACGACGAAACCTCTCCACAAACACGCGACACCGCCGGAGTCAACAGCAACGAT

Exon 7 CGTCAAATACATCCTCTTCACCGTCCCATACCTCCACACCAAGAACCACGACGAAACCTCTCCACAAACACGCGACACCGCCGGAGTCAACAGCAACGAT

11101 11200

*a*  (11071) CCATCCGCCAGGCTACGCGGCAAGGGAACTCCCCAAGACGAACTCAGCGCGAACCATGTCCTCGCTGAGCGACGGAGGAGAGAGAAACTCAACGAGAGGT

*A*  (11061) CCATCCGCCAGGCTACGCGGCAAGGGAACTCCCCAAGACGAACTCAGCGCGAACCATGTCCTCGCTGAGCGACGGAGGAGAGAGAAACTCAACGAGAGGT

Exon 7 CCATCCGCCAGGCTACGCGGCAAGGGAACTCCCCAAGACGAACTCAGCGCGAACCATGTCCTCGCTGAGCGACGGAGGAGAGAGAAACTCAACGAGAGGT

11201 11300

*a*  (11171) TCATAATTCTAAGATCATTGGTTCCTTTTGTTACGAAGATGGATAAAGCTTCTATCTTAGGCGACACGATCGAGTATTTGAAACAGCTTCGGAGAAAGAT

*A*  (11161) TCATAATTCTAAGATCATTGGTTCCTTTTGTTACGAAGATGGATAAAGCTTCTATCTTAGGCGACACGATCGAGTATTTGAAACAGCTTCGGAGAAAGAT

Exon 7 TCATAATTCTAAGATCATTGGTTCCTTTTGTTACGAAGATGGATAAAGCTTCTATCTTAGGCGACACGATCGAGTATTTGAAACAGCTTCGGAGAAAGAT

11301 11400

*a*  (11271) TCAAGATCTCGAGACACGTAACCGTCAGATGGAGTCCGAGAAAAGTGGAGTAACCGTTTTGGTGGGTCCCACTGAGAAGAAAAAAGTGAGGATTGTGGAA

*A*  (11261) TCAAGATCTCGAGACACGTAACCGTCAGATGGAGTCCGAGAAAAGTGGAGTAACCGTTTTGGTGGGTCCCACTGAGAAGAAAAAAGTGAGGATCGTGGAA

Exon 7 TCAAGATCTCGAGACACGTAACCGTCAGATGGAGTCCGAGAAAAGTGGAGTAACCGTTTTGGTGGGTCCCACTGAGAAGAAAAAAGTGAGGATCGTGGAA

11401 11500

*a*  (11371) GGGAATGGCACTGGTGGGGGCGTTAGAGCGAAAGCAGTTGAGGTTGTTGCGTCGGTTCAGGTTTCGATTATAGAGAGTGATGCTTTGTTGGAGATTGAAT

*A*  (11361) GGGAATGGCACTGGTGGGGGCGTCAGAGCGAAAGCAGTTGAGGTTGTTGCGTCGGTTCAGGTTTCGATTATAGAGAGTGATGCTTTGTTGGAGATTGAAT

Exon 7 GGGAATGGCACTGGTGGGGGCGTCAGAGCGAAAGCAGTTGAGGTTGTTGCGTCGGTTCAGGTTTCGATTATAGAGAGTGATGCTTTGTTGGAGATTGAAT

11501 11600

*a*  (11471) GTTTACAAAGAGAAGGGTTGTTGTTGGATGTTATGATGATGTTGAGAGAGTTGAGAATTGAGGTTATTGGAGTTCAATCTTCGCTCAACAATGGCGTTTT

*A*  (11461) GTTTACAAAGAGAAGGGTTGTTGTTGGATGTTATGATGATGTTGAGAGAGTTGAGAATTGAGGTTATTGGAGTTCAATCTTCGCTCAACAATGGCGTTTT

Exon 7 GTTTACAAAGAGAAGGGTTGTTGTTGGATGTTATGATGATGTTGAGAGAGTTGAGAATTGAGGTTATTGGAGTTCAATCTTCGCTCAACAATGGCGTTTT

11601 11700

*a*  (11571) CGTCGCGGAATTGAGGGCTAAGGTTAAGGAAAATGGTAATGGGAAGAAAGTTAGTATTGTGGAAGTTAAGAGAGCGCTTAACCAAATTATACCTCATAAT

*A*  (11561) CGTCGCGGAATTGAGGGCTAAGGTTAAGGAAAATGGTAATGGGAAGAAAGTTAGTATTGTGGAAGTTAAGAGAGCGCTTAACCAAATTATACCTCATAAT

Exon 7 CGTCGCGGAATTGAGGGCTAAGGTTAAGGAAAATGGTAATGGGAAGAAAGTTAGTATTGTGGAAGTTAAGAGAGCGCTTAACCAAATTATACCTCATAAT

11701 11800

*a*  (11671) AATATTTAGTGTATGTTTGAATTCACAGCGAGTTTGGCAAAATCACAAAATCACAATGATTGTTGTGATTCTGTCAAACTCGCTGTTAATCCAAACATAG

*A*  (11661) AATATTTAGTGTATGTTTGAATTCACAGCGAGTTTGGCAAAATCACAAAATCACAATGATTGTTGTGATTCTGTCAAACTCGTTGTTAATCCAAACATAG

Exon 7 AATATTTAG

11801 11900

*a*  (11771) TCTAATCAATGATACATCATTTGTATACATTGACTTATAATATGTACATAAAACTCTCGACTCGAGTCTGATGATATGAAGTGAAGTGAAGTCTAATGAT

*A*  (11761) TCTAATCAATGATACATCATTTGTATACATTGACTTATAATATGTACATAAAACTCTCGACTCGAGTCTGATGATATGAAGTGAAGTGAAGTCTAATGAT

11901 11932

*a*  (11871) AAACAACATCTGCGGATTTGGATTCTCCCCCG

*A*  (11861) AAACAACATCTGCGGATTTGGATTCTCCCCCG

(*B*) Alignment of predicted mRNA from PI 269818 (*A*) and Caméor (*a*) genotypes. ATG start codons and TAG stop codons are marked and in bold. Nucleotide differences are highlighted. The first 8 bases of intron 6 that are included in mRNA of the Caméor (*a*) genotype are marked with asterisks (********).

1 **ATG**

*a*  (1) -CCACATGTTCTTTCCTTTAATTTTGCTGTTTCTCGCTTTTTTACTACTTTTGAAAGTTATGATTCTTTCTCAGAGAACTGAATGAGCCAAGAAGA**ATG**A

*A*  (1) ACCACATGTTCTTTC-TTTAATTTTGCTGTTTCTCGCTTTTTTACTACTTTTGAAAGTTATGATTCTTTCTCAGAGAACTGAATGAGCCAAGAAGA**ATG**A

101 200

*a*  (100) CTGCTCCAACTCCGGAAAACGGTTGTAATAAGCTTCAAAACATGTTGCAGGCTGCAGTGCAATCGGTTCAATGGACTTATAGCCTCTTCTGGCAAATTTG

*A*  (100) CTGCTCCAACTCCGGAAAACGGTTGTAATAAGCTTCAAAACATGTTGCAGGCTGCAGTGCAATCGGTTCAATGGACTTATAGCCTCTTCTGGCAAATTTG

201 300

*a*  (200) CCCACAACAATTGATACTGGTTTGGGGCGATGGATATTACAACGGAGCAATTAAGACACGAAAGACAGTGCAACCAATGGAGGTTAGTGCAGAAGAAGCT

*A*  (200) CCCACAACAATTGATACTGGTTTGGGGCGATGGATATTACAACGGAGCAATTAAGACACGAAAGACAGTGCAACCAATGGAGGTTAGTGCAGAAGAAGCT

301 400

*a*  (300) TCTCTACAAAGAAGCCAGCAACTAAGAGAGCTGTATGAATCGTTATCGGCCGGAGAGACAAATCCGCCAACGCGTCGACCGTGTGCTTCTTTGTCGCCTG

*A*  (300) TCTCTACAAAGAAGCCAGCAACTAAGAGAGCTGTATGAATCGTTATCGGCCGGAGAGACAAATCCGCCAACGCGTCGACCGTGTGCTTCTTTGTCGCCTG

401 500

*a*  (400) AAGACTTAACAGAATCTGAATGGTTTTATTTGATGTGTGTCTCTTTCTCATTTCCTCCTGGTGTCGGGTTGCCAGGAAAGGCATATGCTAGGAGGCAACA

*A*  (400) AAGACTTAACAGAATCTGAATGGTTTTATTTGATGTGTGTCTCTTTCTCATTTCCTCCTGGTGTCGGGTTGCCAGGAAAGGCATATGCTAGGAGGCAACA

501 600

*a*  (500) TGTATGGCTCACGGGTGCAAATGAGGTGGATAGCAAAACATTTTCAAGAGCTATTTTAGCCAAGAGTGCTAATATACAGACTGTGGTATGCATTCCTGTG

*A*  (500) TGTATGGCTCACGGGAGCAAATGAGGTGGATAGCAAAACATTTTCAAGAGCTATTTTAGCCAAGAGTGCTAATATACAGACTGTGGTATGCATTCCTGTG

601 700

*a*  (600) TTGGATGGTGTCGTTGAGATTGGCACAACTGATAAGGTTCAAGAAGATCTTAATTTCATCAAACACGTGAGGAGTTTCTTCATAGACCACCACTCTTTGC

*A*  (600) TTGGATGGTGTCGTTGAGATTGGCACAACTGATAAGATTCAAGAAGATCTTAATTTCATCAAACACGTGAGGAGTTTCTTCATAGACCACCACTCTTTGC

701 800

*a*  (700) CACCAAAGCCAGCACTCTCTGAACACTCAACCTCCAATCCGACTTACTCAACCGATCACATTCCTGCCATTATGTACACAGTGGCAGACCCAGCCTCCAC

*A*  (700) CACCAAAGCCAGCACTCTCAGAACACTCAACCTCCAATCCGACTTACTCAACCGATCACATTCCTGCCATTATGTACACAGTGGCAGACCCAGCCTCCAC

801 900

*a*  (800) GACAATTCCCAATCAAGACGATATGGATGAAGATGAGGAAGAGGATGACGAAGATGACGAAGTTGAATCTGGATCTGAAGATGAAACCAACCAAGGTCAT

*A*  (800) GGCAATTCCCAATCAAGACGATATGGATGAAGATGAGGAAGAGGATGATGAAGATGACGAAGTTGAATCTGGATCCGAAGATGAAACCAATCAAGGTCAT

901 1000

*a*  (900) AACCAACACGCAACCTCTATAATAGAGGCTGCGGAACCGAGTGAACTCATGCAAATTGAAATGCCCGATGATATTCGGATCGGGTCACCCAACGATGGGT

*A*  (900) AACCAACACGCAACCTCTATAATAGAGGCTGCGGAACCGAGTGAACTCATGCAAATTGAAATGCCCGATGATATTCGGATCGGGTCACCCAACGACGGGT

1001 1100

*a*  (1000) CGAATAATTTGGACTCGGATTTTCATTTGTTGGCCGTTAGTAATCAAGGAAACCCATCAAGACAAATTGACTCATATACAACCGAGAGATGGGGTCCAAT

*A*  (1000) CAAATAATTTAGACTCGGATTTTCATTTGTTGGCCGTTAGTAATCAAGGAAACCCATCAAGACAAATTGACTCATATACAACTGAGAGATGGGGTCCAAT

1101 ******** **TAG** 1200

*a*  (1100) CGAAGAACCTCTCGATGATTCACTACAAGTTCAATTATCATCTTCAGATAAATCGTACTTCATCATCCATTAGAAGACTTAACACAAGAAGACACACACT

*A*  (1100) CGAAGAACCTCTCGATGATTCACTACAAATTCAATTATCATCTTCAG--------TACTTCATCATCCATTAGAAGACTTAACACAAGAAGACACACACT

1201 1300

*a*  (1200) ACTCTCAAACAGTAACCACCATTCTCCAAAACCAATGGATCGATTCACCTTCCATCAACTACATCAACTACTCCACCCAATCATCTTTCACCACCTGGAC

*A*  (1192) ACTCTCAAACAGTAACCACCATTCTCCAAAACCAATGGATCGATTCACCTTCCATCAACTACATCAACTACTCCACCCAATCATCTTTCACCACCTGGAC

1301 1400

*a*  (1300) CAACCACCACTTCCACCCGCCGCCGCCGCCGGACCCTGCCACCTCCCAGTGGCTCCTCAAATACATCCTCTTCACCGTCCCATACCTCCACACCAAGAAC

*A*  (1292) CAACCACCACTTCCACCCGCCGCCGCCGCCGGACCCTGCCACCTCCCAATGGCTCGTCAAATACATCCTCTTCACCGTCCCATACCTCCACACCAAGAAC

1401 1500

*a*  (1400) CACGACGAAACCTCTCCACAAACACGCGACACCGCCGGAGTCAACAGCAACGATCCATCCGCCAGGCTACGCGGCAAGGGAACTCCCCAAGACGAACTCA

*A*  (1392) CACGACGAAACCTCTCCACAAACACGCGACACCGCCGGAGTCAACAGCAACGATCCATCCGCCAGGCTACGCGGCAAGGGAACTCCCCAAGACGAACTCA

1501 1600

*a*  (1500) GCGCGAACCATGTCCTCGCTGAGCGACGGAGGAGAGAGAAACTCAACGAGAGGTTCATAATTCTAAGATCATTGGTTCCTTTTGTTACGAAGATGGATAA

*A*  (1492) GCGCGAACCATGTCCTCGCTGAGCGACGGAGGAGAGAGAAACTCAACGAGAGGTTCATAATTCTAAGATCATTGGTTCCTTTTGTTACGAAGATGGATAA

1601 1700

*a*  (1600) AGCTTCTATCTTAGGCGACACGATCGAGTATTTGAAACAGCTTCGGAGAAAGATTCAAGATCTCGAGACACGTAACCGTCAGATGGAGTCCGAGAAAAGT

*A*  (1592) AGCTTCTATCTTAGGCGACACGATCGAGTATTTGAAACAGCTTCGGAGAAAGATTCAAGATCTCGAGACACGTAACCGTCAGATGGAGTCCGAGAAAAGT

1701 1800

*a*  (1700) GGAGTAACCGTTTTGGTGGGTCCCACTGAGAAGAAAAAAGTGAGGATTGTGGAAGGGAATGGCACTGGTGGGGGCGTTAGAGCGAAAGCAGTTGAGGTTG

*A*  (1692) GGAGTAACCGTTTTGGTGGGTCCCACTGAGAAGAAAAAAGTGAGGATCGTGGAAGGGAATGGCACTGGTGGGGGCGTCAGAGCGAAAGCAGTTGAGGTTG

1801 1900

*a*  (1800) TTGCGTCGGTTCAGGTTTCGATTATAGAGAGTGATGCTTTGTTGGAGATTGAATGTTTACAAAGAGAAGGGTTGTTGTTGGATGTTATGATGATGTTGAG

*A*  (1792) TTGCGTCGGTTCAGGTTTCGATTATAGAGAGTGATGCTTTGTTGGAGATTGAATGTTTACAAAGAGAAGGGTTGTTGTTGGATGTTATGATGATGTTGAG

1901 2000

*a*  (1900) AGAGTTGAGAATTGAGGTTATTGGAGTTCAATCTTCGCTCAACAATGGCGTTTTCGTCGCGGAATTGAGGGCTAAGGTTAAGGAAAATGGTAATGGGAAG

*A*  (1892) AGAGTTGAGAATTGAGGTTATTGGAGTTCAATCTTCGCTCAACAATGGCGTTTTCGTCGCGGAATTGAGGGCTAAGGTTAAGGAAAATGGTAATGGGAAG

2001 **TAG** 2100

*a*  (2000) AAAGTTAGTATTGTGGAAGTTAAGAGAGCGCTTAACCAAATTATACCTCATAATAATATT**TAG**TGTATGTTTGAATTCACAGCGAGTTTGGCAAAATCAC

*A*  (1992) AAAGTTAGTATTGTGGAAGTTAAGAGAGCGCTTAACCAAATTATACCTCATAATAATATT**TAG**TGTATGTTTGAATTCACAGCGAGTTTGGCAAAATCAC

2101 2200

*a*  (2100) AAAATCACAATGATTGTTGTGATTCTGTCAAACTCGCTGTTAATCCAAACATAGTCTAATCAATGATACATCATTTGTATACATTGACTTATAATATGTA

*A*  (2092) AAAATCACAATGATTGTTGTGATTCTGTCAAACTCGTTGTTAATCCAAACATAGTCTAATCAATGATACATCATTTGTATACATTGACTTATAATATGTA

2201 2287

*a*  (2200) CATAAAACTCTCGACTCGAGTCTGATGATATGAAGTGAAGTGAAGTCTAATGATAAACAACATCTGCGGATTTGGATTCTCCCCCGC

*A*  (2192) CATAAAACTCTCGACTCGAGTCTGATGATATGAAGTGAAGTGAAGTCTAATGATAAACAACATCTGCGGATTTGGATTCTCCCCCGC

(*C*) Alignment of the predicted bHLH coding sequence from purple-flowered A (PI 269818 and JI 2822) and white flowered a genotypes (Caméor and JI 1987). Amino acid sequence differences are highlighted and stop codons are marked with asterisks (*).

*A*  JI 2822 (1) MTAPTPENGCNKLQNMLQAAVQSVQWTYSLFWQICPQQLILVWGDGYYNGAIKTRKTVQPMEVSAEEASLQRSQQLRELYESLSAGETNPPTRRPCASLS

*A*  PI 269818 (1) MTAPTPENGCNKLQNMLQAAVQSVQWTYSLFWQICPQQLILVWGDGYYNGAIKTRKTVQPMEVSAEEASLQRSQQLRELYESLSAGETNPPTRRPCASLS

*a*  Caméor (1) MTAPTPENGCNKLQNMLQAAVQSVQWTYSLFWQICPQQLILVWGDGYYNGAIKTRKTVQPMEVSAEEASLQRSQQLRELYESLSAGETNPPTRRPCASLS

*a*  JI 1987 (1) MTAPTPENGCNKLQNMLQAAVQSVQWTYSLFWQICPQQLILVWGDGYYNGAIKTRKTVQPMEVSAEEASLQRSQQLRELYESLSAGETNPPTRRPCASLS

101 200

*A*  JI 2822 (101) PEDLTESEWFYLMCVSFSFPPGVGLPGKAYARRQHVWLTGANEVDSKTFSRAILAKSANIQTVVCIPVLDGVVEIGTTDKVQEDLNFIKHVRSFFIDHHS

*A*  PI 269818(101) PEDLTESEWFYLMCVSFSFPPGVGLPGKAYARRQHVWLTGANEVDSKTFSRAILAKSANIQTVVCIPVLDGVVEIGTTDKIQEDLNFIKHVRSFFIDHHS

*a*  Caméor (101) PEDLTESEWFYLMCVSFSFPPGVGLPGKAYARRQHVWLTGANEVDSKTFSRAILAKSANIQTVVCIPVLDGVVEIGTTDKVQEDLNFIKHVRSFFIDHHS

*a*  JI 1987 (101) PEDLTESEWFYLMCVSFSFPPGVGLPGKAYARRQHVWLTGANEVDSKTFSRAILAKSANIQTVVCIPVLDGVVEIGTTDKVQEDLNFIKHVRSFFIDHHS

201 300

*A*  JI 2822 (201) LPPKPALSEHSTSNPTYSTDHIPAIMYTVADPASTTIPNQDDMDEDEEEDDEDDEVESGSEDETNQGHNQHATSIIEAAEPSELMQIEMPDDIRIGSPND

*A*  PI 269818(201) LPPKPALSEHSTSNPTYSTDHIPAIMYTVADPASTAIPNQDDMDEDEEEDDEDDEVESGSEDETNQGHNQHATSIIEAAEPSELMQIEMPDDIRIGSPND

*a*  Caméor (201) LPPKPALSEHSTSNPTYSTDHIPAIMYTVADPASTTIPNQDDMDEDEEEDDEDDEVESGSEDETNQGHNQHATSIIEAAEPSELMQIEMPDDIRIGSPND

*a*  JI 1987 (201) LPPKPALSEHSTSNPTYSTDHIPAIMYTVADPASTAIPNQDDMDEDEEEDDEDDEVEIWIRR*

301 400

*A*  JI2822 (301) GSNNLDSDFHLLAVSNQGNPSRQIDSYTTERWGPIEEPLDDSLQVQLSSSVLHHPLEDLTQEDTHYSQTVTTILQNQWIDSPSINYINYSTQSSFTTWTN

*A*  PI269818(301) GSNNLDSDFHLLAVSNQGNPSRQIDSYTTERWGPIEEPLDDSLQIQLSSSVLHHPLEDLTQEDTHYSQTVTTILQNQWIDSPSINYINYSTQSSFTTWTN

*a*  Caméor (301) GSNNLDSDFHLLAVSNQGNPSRQIDSYTTERWGPIEEPLDDSLQVQLSSSDKSYFIIH*

401 500

*A*  PI 269818(401) HHFHPPPPPDPATSQWLVKYILFTVPYLHTKNHDETSPQTRDTAGVNSNDPSARLRGKGTPQDELSANHVLAERRRREKLNERFIILRSLVPFVTKMDKA

*A*  JI 2822 (401) HHFHPPPPPDPATSQWLVKYILFTVPYLHTKNHDETSPQTRDTAGVNSNDPSARLRGKGTPQDELSANHVLAERRRREKLNERFIILRSLVPFVTKMDKA

501 600

*A*  PI 269818(501) SILGDTIEYLKQLRRKIQDLETRNRQMESEKSGVTVLVGPTEKKKVRIVEGNGTGGGVRAKAVEVVASVQVSIIESDALLEIECLQREGLLLDVMMMLRE

*A*  JI 2822 (501) SILGDTIEYLKQLRRKIQDLETRNRQMESEKSGVTVLVGPTEKKKVRIVEGNGTGGGVRAKAVEVVASVQVSIIESDALLEIECLQREGLLLDVMMMLRE

601 652

*A*  PI 269818(601) LRIEVIGVQSSLNNGVFVAELRAKVKENGNGKKVSIVEVKRALNQIIPHNNI*

*A*  JI 2822 (601) LRIEVIGVQSSLNNGVFVAELRAKVKENGNGKKVSIVEVKRALNQIIPHNNI*
