# Supplementary material for: Identification of Mendel's White Flower Character
Source: PLoS One. 2010 Oct 11;5(10):e13230. doi: 10.1371/journal.pone.0013230 (PMC2952588; doi:10.1371/journal.pone.0013230)
Supplement: Figure S3 — Sequence divergence of the bHLH gene in germplasm and exotic pea lines. (A) Sequence of exotic pea lines. For JI 4 and 9 exotic white flowered pea lines; JI 232, JI 616, JI 817, JI 1497, JI 1512, JI 1782, JI 1987, JI 2647 and JI 3003 the G to A mutation is highlighted and marked with *. Three independent individuals of the white flowered JI 1987 (n1, n2 and n3) do not have the G to A transition, they do, however, have a number of SNPs that are highlighted in light blue and an additional A residue in exon 6, identified by the # and highlighted in red. (B) Association of pea flower color with the G to A mutation at base 5005 (highlighted) in 148 lines (including 129 single plant representatives from the refined core of PI lines see USDA ARS Germplasm Resources Information Network at: http://www.ars-grin.gov). All colored flower lines (n = 60) carry the G allele that corresponds to correct splicing of intron 6, while most white flowered lines (n = 78) have the A mutation, resulting in mis-splicing. Ten white flowered lines carry the G allele, and seven of these have been found to carry the JI 1987 indel allele. For three white flowered lines neither mutation has been identified. (C) Flower color phenotype and alignment of SNPs at the junction between exon 6 and intron 6 of the bHLH gene of 148 pea lines. Within a window of 145 nt including the splice donor site (at nt 22) there are 21 SNPs and one indel (GTTGTATTTAG, present only in PI 273209) that are shown. The first SNP is in exon 6 and the second is the G/A transition at the intron 6 splice donor site. Polymorphic nucleotides are colored to make the haplotypes easy to see. The column to the left indicates flower color pink = colored flower, white = white. The lines with the splice donor site mutation and the JI 1987 indel are indicated. The three sequences for which a mutation in the A gene has not been identified are arrowed. To the right an unweighted UPGMA tree based on these sequences is shown and the accession [file pone.0013230.s003.doc]

**Figure. S3. Sequence divergence of the *bHLH* gene in germplasm and exotic pea lines.**

(*A*) Sequence of exotic pea lines. For JI 4 and 9 exotic white flowered pea lines; JI 232, JI 616, JI 817, JI 1497, JI 1512, JI 1782, JI 1987, JI 2647 and JI 3003 the G to A mutation is highlighted and marked with *. Three independent individuals of the white flowered JI 1987 (n1, n2 and n3) do not have the G to A transition, they do, however, have a number of SNPs that are highlighted in light blue and an additional A residue in exon 6, identified by the # and highlighted in red. #

**JI 4**  AAGTTGAA-T CTGGATCTGA AGATGAAACC AACCAAGGTC ATAACCAACA CGCAACCTCT

**JI 232**  AAGTTGAA-T CTGGATCTGA AGATGAAACC AACCAAGGTC ATAACCAACA CGCAACCTCT

**JI 616**  AAGTTGAA-T CTGGATCTGA AGATGAAACC AACCAAGGTC ATAACCAACA CGCAACCTCT

**JI 817**  AAGTTGAA-T CTGGATCTGA AGATGAAACC AACCAAGGTC ATAACCAACA CGCAACCTCT

**JI 1497**  AAGTTGAA-T CTGGATCTGA AGATGAAACC AACCAAGGTC ATAACCAACA CGCAACCTCT

**JI 1512**  AAGTTGAA-T CTGGATCTGA AGATGAAACC AACCAAGGTC ATAACCAACA CGCAACCTCT

**JI 1782**  AAGTTGAA-T CTGGATCTGA AGATGAAACC AACCAAGGTC ATAACCAACA CGCAACCTCT

**JI 1987n1** AAGTTGAAAT CTGGATCCGA AGATGAAACC AATCAAGGTC ATAACCAACA CGCAACCTCT

**JI 1987n2** AAGTTGAAAT CTGGATCCGA AGATGAAACC AATCAAGGTC ATAACCAACA CGCAACCTCT

**JI 1987n3** AAGTTGAAAT CTGGATCCGA AGATGAAACC AATCAAGGTC ATAACCAACA CGCAACCTCT

**JI 2647**  AAGTTGAA-T CTGGATCTGA AGATGAAACC AACCAAGGTC ATAACCAACA CGCAACCTCT

**JI 3003**  AAGTTGAA-T CTGGATCTGA AGATGAAACC AACCAAGGTC ATAACCAACA CGCAACCTCT

**JI 4**  ATAATAGAGG CTGCGGAACC GAGTGAACTC ATGCAAATTG AAATGCCCGA TGATATTCGG

**JI 232**  ATAATAGAGG CTGCGGAACC GAGTGAACTC ATGCAAATTG AAATGCCCGA TGATATTCGG

**JI 616**  ATAATAGAGG CTGCGGAACC GAGTGAACTC ATGCAAATTG AAATGCCCGA TGATATTCGG

**JI 817**  ATAATAGAGG CTGCGGAACC GAGTGAACTC ATGCAAATTG AAATGCCCGA TGATATTCGG

**JI 1497**  ATAATAGAGG CTGCGGAACC GAGTGAACTC ATGCAAATTG AAATGCCCGA TGATATTCGG

**JI 1512**  ATAATAGAGG CTGCGGAACC GAGTGAACTC ATGCAAATTG AAATGCCCGA TGATATTCGG

**JI 1782**  ATAATAGAGG CTGCGGAACC GAGTGAACTC ATGCAAATTG AAATGCCCGA TGATATTCGG

**JI 1987n1** ATAATAGAGG CTGCGGAACC GAGTGAACTC ATGCAAATTG AAATGCCCGA TGATATTCGG

**JI 1987n2** ATAATAGAGG CTGCGGAACC GAGTGAACTC ATGCAAATTG AAATGCCCGA TGATATTCGG

**JI 1987n3** ATAATAGAGG CTGCGGAACC GAGTGAACTC ATGCAAATTG AAATGCCCGA TGATATTCGG

**JI 2647**  ATAATAGAGG CTGCGGAACC GAGTGAACTC ATGCAAATTG AAATGCCCGA TGATATTCGG

**JI 3003**  ATAATAGAGG CTGCGGAACC GAGTGAACTC ATGCAAATTG AAATGCCCGA TGATATTCGG

**JI 4**  ATCGGGTCAC CCAACGATGG GTCGAATAAT TTGGACTCGG ATTTTCATTT GTTGGCCGTT

**JI 232**  ATCGGGTCAC CCAACGATGG GTCGAATAAT TTGGACTCGG ATTTTCATTT GTTGGCCGTT

**JI 616**  ATCGGGTCAC CCAACGATGG GTCGAATAAT TTGGACTCGG ATTTTCATTT GTTGGCCGTT

**JI 817**  ATCGGGTCAC CCAACGATGG GTCGAATAAT TTGGACTCGG ATTTTCATTT GTTGGCCGTT

**JI 1497**  ATCGGGTCAC CCAACGATGG GTCGAATAAT TTGGACTCGG ATTTTCATTT GTTGGCCGTT

**JI 1512**  ATCGGGTCAC CCAACGATGG GTCGAATAAT TTGGACTCGG ATTTTCATTT GTTGGCCGTT

**JI 1782**  ATCGGGTCAC CCAACGATGG GTCGAATAAT TTGGACTCGG ATTTTCATTT GTTGGCCGTT

**JI 1987n1** ATCGGGTCAC CCAACGACGG GTCAAATAAT TTAGACTCGG ATTTTCATTT GTTGGCCGTT

**JI 1987n2** ATCGGGTCAC CCAACGACGG GTCAAATAAT TTAGACTCGG ATTTTCATTT GTTGGCCGTT

**JI 1987n3** ATCGGGTCAC CCAACGACGG GTCAAATAAT TTAGACTCGG ATTTTCATTT GTTGGCCGTT

**JI 2647**  ATCGGGTCAC CCAACGATGG GTCGAATAAT TTGGACTCGG ATTTTCATTT GTTGGCCGTT

**JI 3003**  ATCGGGTCAC CCAACGATGG GTCGAATAAT TTGGACTCGG ATTTTCATTT GTTGGCCGTT

**JI 4**  AGTAATCAAG GAAACCCATC AAGACAAATT GACTCATATA CAACCGAGAG ATGGGGTCCA

**JI 232**  AGTAATCAAG GAAACCCATC AAGACAAATT GACTCATATA CAACCGAGAG ATGGGGTCCA

**JI 616**  AGTAATCAAG GAAACCCATC AAGACAAATT GACTCATATA CAACCGAGAG ATGGGGTCCA

**JI 817**  AGTAATCAAG GAAACCCATC AAGACAAATT GACTCATATA CAACCGAGAG ATGGGGTCCA

**JI 1497**  AGTAATCAAG GAAACCCATC AAGACAAATT GACTCATATA CAACCGAGAG ATGGGGTCCA

**JI 1512**  AGTAATCAAG GAAACCCATC AAGACAAATT GACTCATATA CAACCGAGAG ATGGGGTCCA

**JI 1782**  AGTAATCAAG GAAACCCATC AAGACAAATT GACTCATATA CAACCGAGAG ATGGGGTCCA

**JI 1987n1** AGTAATCAAG GAAACCCATC AAGACAAATT GACTCATATA CAACCGAGAG ATGGGGTCCA

**JI 1987n2** AGTAATCAAG GAAACCCATC AAGACAAATT GACTCATATA CAACCGAGAG ATGGGGTCCA

**JI 1987n3** AGTAATCAAG GAAACCCATC AAGACAAATT GACTCATATA CAACCGAGAG ATGGGGTCCA

**JI 2647**  AGTAATCAAG GAAACCCATC AAGACAAATT GACTCATATA CAACCGAGAG ATGGGGTCCA

**JI 3003**  AGTAATCAAG GAAACCCATC AAGACAAATT GACTCATATA CAACCGAGAG ATGGGGTCCA

*

**JI 4**  ATCGAAGAAC CTCTCGATGA TTCACTACAA GTTCAATTAT CATCTTCAGA TAAATCGGTA

**JI 232**  ATCGAAGAAC CTCTCGATGA TTCACTACAA GTTCAATTAT CATCTTCAGA TAAATCGGTA

**JI 616**  ATCGAAGAAC CTCTCGATGA TTCACTACAA GTTCAATTAT CATCTTCAGA TAAATCGGTA

**JI 817**  ATCGAAGAAC CTCTCGATGA TTCACTACAA GTTCAATTAT CATCTTCAGA TAAATCGGTA

**JI 1497**  ATCGAAGAAC CTCTCGATGA TTCACTACAA GTTCAATTAT CATCTTCAGA TAAATCGGTA

**JI 1512**  ATCGAAGAAC CTCTCGATGA TTCACTACAA GTTCAATTAT CATCTTCAGA TAAATCGGTA

**JI 1782**  ATCGAAGAAC CTCTCGATGA TTCACTACAA GTTCAATTAT CATCTTCAGA TAAATCGGTA

**JI 1987n1** ATCGAAGAAC CTCTCGATGA TTCACTACAA GTTCAATTAT CATCTTCAGG TAAATCTTTA

**JI 1987n2** ATCGAAGAAC CTCTCGATGA TTCACTACAA GTTCAATTAT CATCTTCAGG TAAATCTTTA

**JI 1987n3** ATCGAAGAAC CTCTCGATGA TTCACTACAA GTTCAATTAT CATCTTCAGG TAAATCTTTA

**JI 2647**  ATCGAAGAAC CTCTCGATGA TTCACTACAA GTTCAATTAT CATCTTCAGA TAAATCGGTA

**JI 3003**  ATCGAAGAAC CTCTCGATGA TTCACTACAA GTTCAATTAT CATCTTCAGA TAAATCGGTA

(*B*) Association of pea flower color with the G to A mutation at base 5005 (highlighted) in 148 lines (including 129 single plant representatives from the refined core of PI lines http://www.ars-grin.gov). All colored flower lines (n=60) carry the G allele that corresponds to correct splicing of intron 6, while most white flowered lines (n=78) have the A mutation, resulting in mis-splicing. Ten white flowered lines carry the G allele, and seven of these have been found to carry the JI 1987 indel allele. For three white flowered lines neither mutation has been identified.

|  | **TCAGGTAAAT** | **TCAGATAAAT** |
| --- | --- | --- |
| 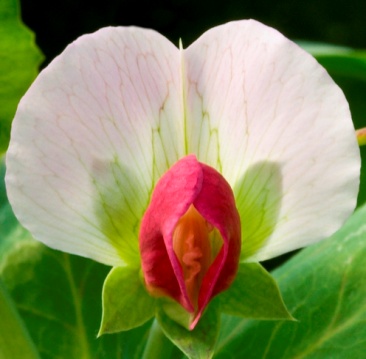 | 60 | 0 |
| 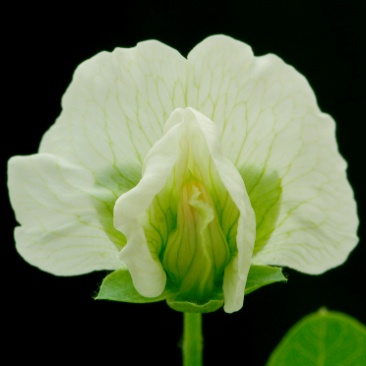 | 10 | 78 |

*C*) Flower color phenotype and alignment of SNPs at the junction between exon 6 and intron 6 of the bHLH gene of 148 pea lines. Within a window of 145 nt including the splice donor site (at nt 22) there are 21 SNPs and one indel (GTTGTATTTAG, present only in PI 273209) that are shown. The first SNP is in exon 6 and the second is the G/A transition at the intron 6 splice donor site. Polymorphic nucleotides are colored to make the haplotypes easy to see. The column to the left indicates flower color pink=colored flower, white=white. The lines with the splice donor site mutation and the JI 1987 indel are indicated. The three sequences for which a mutation in the *A* gene has not been identified are arrowed. To the right an unweighted UPGMA tree based on these sequences is shown and the accessions have been ordered according to a neighbor joining tree constructed on the basis of this tree. The order of accessions (top to bottom) is: PI 102888, PI 116056, PI 125840, PI 143485, PI 116944, PI 125839, PI 198735, PI 207508, PI 220174, PI 220189, PI 222071, PI 222117, PI 340130, PI 210558, PI 103058, PI 116844, PI 117264, PI 121352, PI 124478, PI 134271, PI 156720, PI 163126, PI 163129, PI 164182, PI 164548, PI 164612, PI 169603, PI 171810, PI 172339, PI 173840, PI 179459, PI 179970, PI 180696, PI 181799, PI 181801, PI 181958, PI 184130, PI 193584, PI 195404, PI 197044, PI 201390, PI 203066, PI 203067, PI 203068, PI 203069, PI 206838, PI 209507, PI 210561, PI 210568, PI 210569, PI 210571, PI 210583, PI 212031, PI 212917, PI 221697, PI 242028, PI 244093, PI 248181, PI 257244, PI 261624, PI 263027, PI 269798, PI 269821, PI 271035, PI 271511, PI 279825, PI 280603, PI 280611, PI 280616, PI 285715, PI 286431, PI 286607, PI 343987, PI 411143, JI 232, JI 616, JI 871, JI 1497, JI 1512, JI 1792, JI 2647, JI 3003, JI 4, Cameor, JI 504, JI 1189, JI 1229, JI 2737, JI 2462, JI 2479, Torsdag, PI 169608, PI 180693, PI 184128, PI 193590, PI 195020, PI 195631, PI 257592, PI 269782, PI 272204, PI 272218, PI 285719, PI 331414, PI 340128, PI 343331, PI 270536, PI 280619, PI 193578, PI 179450, PI 142775, PI 184784, PI 206861, PI 269825, PI 331413, PI 343824, JI 1987, PI 109866, PI 137118, PI 137119, PI 140298, PI 155109, PI 156647, PI 164972, PI 174320, PI 180329, PI 180702, PI 197990, PI 198072, PI 204306, PI 343958, PI 198074, PI 266070, PI 117998, PI 179451, PI 203064, PI 164971, PI 188698, PI 242027, PI 269818, PI 162909, PI 179722, PI 165949, PI 166084, PI 166159, PI 174921, PI 164779, PI 271033, PI 273209.

*C*

*
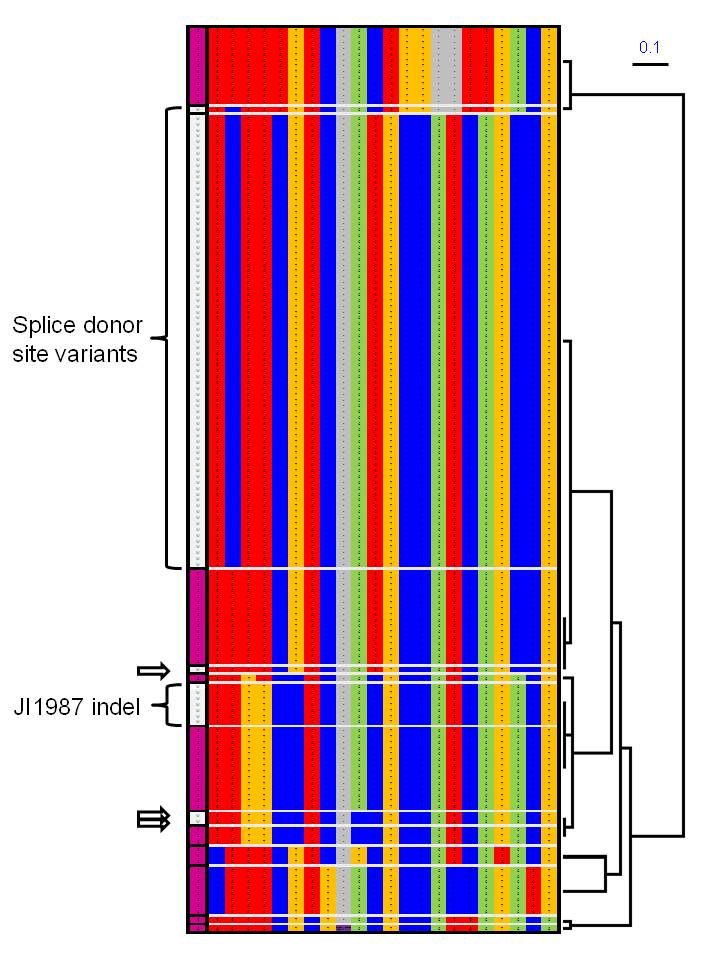
*

(*D*) FASTA sequence of the region between exon 6 and intron 6 that was used for tree construction.

>PI102888

AAGTTCAATTATCATCTTCAGGTAAATCGGTAATTGACAAATTTAAATTTCAAATATGTTTTGAATCCTTGTATTTAG-----------GTTTGTCCTAACTCCTAACCCGTT--AGGTTCCCTAAATTTTATGTGCCAAATAAATTTATAAAA-AAAAAAACATAAAGACATGATGCGGGTGGACTCACAAAGTGAAGCATACAAGTCATACAAGTTATAAAGTTTTTGAAAGCGAAATAACTTTTTAAATTTTTTTAAATATTATAATCGGATATTTCTACGACTAACAACTGAACTGAATTAACGGAATAATTAATCAATCATGTATTGATGTAAGCGAATTGGACCACATCAGGGTACTTAGAGTTTGGGGGGCTAGCTAGTGATTAGATCCAAAGTTTTTTTCACCACATATCAATGTGGTCCATCCTAATTAAGTCCATTGTCTACCCCATTTTTGGTGGGGTCCCCAATCTATGAAAAA-TCGAATTCATTTTACTGCTCACTCACCAAAAATGTTCAACAGCCCCACGGTACATTATACAAGTAATAGTAATACAACAACATACATTAAACTTATATAG

>PI116056

AAGTTCAATTATCATCTTCAGGTAAATCGGTAATTGACAAATTTAAATTTCAAATATGTTTTGAATCCTTGTATTTAG-----------GTTTGTCCTAACTCCTAACCCGTT--AGGTTCCCTAAATTTTATGTGCCAAATAAATTTATAAAA-AAAAAAACATAAAGACATGATGCGGGTGGACTCACAAAGTGAAGCATACAAGTCATACAAGTTATAAAGTTTTTGAAAGCGAAATAACTTTTTAAATTTTTTTAAATATTATAATCGGATATTTCTACGACTAACAACTGAACTGAATTAACGGAATAATTAATCAATCATGTATTGATGTAAGCGAATTGGACCACATCAGGGTACTTAGAGTTTGGGGGGCTAGCTAGTGATTAGATCCAAAGTTTTTTTCACCACATATCAATGTGGTCCATCCTAATTAAGTCCATTGTCTACCCCATTTTTGGTGGGGTCCCCAATCTATGAAAAA-TCGAATTCATTTTACTGCTCACTCACCAAAAATGTTCAACAGCCCCACGGTACATTATACAAGTAATAGTAATACAACAACATACATTAAACTTATATAG

>PI125840

AAGTTCAATTATCATCTTCAGGTAAATCGGTAATTGACAAATTTAAATTTCAAATATGTTTTGAATCCTTGTATTTAG-----------GTTTGTCCTAACTCCTAACCCGTT--AGGTTCCCTAAATTTTATGTGCCAAATAAATTTATAAAA-AAAAAAACATAAAGACATGATGCGGGTGGACTCACAAAGTGAAGCATACAAGTCATACAAGTTATAAAGTTTTTGAAAGCGAAATAACTTTTTAAATTTTTTTAAATATTATAATCGGATATTTCTACGACTAACAACTGAACTGAATTAACGGAATAATTAATCAATCATGTATTGATGTAAGCGAATTGGACCACATCAGGGTACTTAGAGTTTGGGGGGCTAGCTAGTGATTAGATCCAAAGTTTTTTTCACCACATATCAATGTGGTCCATCCTAATTAAGTCCATTGTCTACCCCATTTTTGGTGGGGTCCCCAATCTATGAAAAA-TCGAATTCATTTTACTGCTCACTCACCAAAAATGTTCAACAGCCCCACGGTACATTATACAAGTAATAGTAATACAACAACATACATTAAACTTATATAG

>PI143485

AAGTTCAATTATCATCTTCAGGTAAATCGGTAATTGACAAATTTAAATTTCAAATATGTTTTGAATCCTTGTATTTAG-----------GTTTGTCCTAACTCCTAACCCGTT--AGGTTCCCTAAATTTTATGTGCCAAATAAATTTATAAAA-AAAAAAACATAAAGACATGATGCGGGTGGACTCACAAAGTGAAGCATACAAGTCATACAAGTTATAAAGTTTTTGAAAGCGAAATAACTTTTTAAATTTTTTTAAATATTATAATCGGATATTTCTACGACTAACAACTGAACTGAATTAACGGAATAATTAATCAATCATGTATTGATGTAAGCGAATTGGACCACATCAGGGTACTTAGAGTTTGGGGGGCTAGCTAGTGATTAGATCCAAAGTTTTTTTCACCACATATCAATGTGGTCCATCCTAATTAAGTCCATTGTCTACCCCATTTTTGGTGGGGTCCCCAATCTATGAAAAA-TCGAATTCATTTTACTGCTCACTCACCAAAAATGTTCAACAGCCCCACGGTACATTATACAAGTAATAGTAATACAACAACATACATTAAACTTATATAG

>PI116944

AAGTTCAATTATCATCTTCAGGTAAATCGGTAATTGACAAATTTAAATTTCAAATATGTTTTGAATCCTTGTATTTAG-----------GTTTGTCCTAACTCCTAACCCGTT--AGGTTCCCTAAATTTTATGTGCCAAATAAATTTATAAAA-AAAAAAACATAAAGACATGATGCGGGTGGACTCACAAAGTGAAGCATACAAGTCATACAAGTTATAAAGTTTTTGAAAGCGAAATAACTTTTTAAATTTTTTTAAATATTATAATCGGATATTTCTACGACTAACAACTGAACTGAATTAACGGAATAATTAATCAATCATGTATTGATGTAAGCGAATTGGACCACATCAGGGTACTTAGAGTTTGGGGGGCTAGCTAGTGATTAGATCCAAAGTTTTTTTCACCACATATCAATGTGGTCCATCCTAATTAAGTCCATTGTCTACCCCATTTTTGGTGGGGTCCCCAATCTATGAAAAA-TCGAATTCATTTTACTGCTCACTCACCAAAAATGTTCAACAGCCCCACGGTACATTATACAAGTAATAGTAATACAACAACATACATTAAACTTATATAG

>PI125839

AAGTTCAATTATCATCTTCAGGTAAATCGGTAATTGACAAATTTAAATTTCAAATATGTTTTGAATCCTTGTATTTAG-----------GTTTGTCCTAACTCCTAACCCGTT--AGGTTCCCTAAATTTTATGTGCCAAATAAATTTATAAAA-AAAAAAACATAAAGACATGATGCGGGTGGACTCACAAAGTGAAGCATACAAGTCATACAAGTTATAAAGTTTTTGAAAGCGAAATAACTTTTTAAATTTTTTTAAATATTATAATCGGATATTTCTACGACTAACAACTGAACTGAATTAACGGAATAATTAATCAATCATGTATTGATGTAAGCGAATTGGACCACATCAGGGTACTTAGAGTTTGGGGGGCTAGCTAGTGATTAGATCCAAAGTTTTTTTCACCACATATCAATGTGGTCCATCCTAATTAAGTCCATTGTCTACCCCATTTTTGGTGGGGTCCCCAATCTATGAAAAA-TCGAATTCATTTTACTGCTCACTCACCAAAAATGTTCAACAGCCCCACGGTACATTATACAAGTAATAGTAATACAACAACATACATTAAACTTATATAG

>PI198735

AAGTTCAATTATCATCTTCAGGTAAATCGGTAATTGACAAATTTAAATTTCAAATATGTTTTGAATCCTTGTATTTAG-----------GTTTGTCCTAACTCCTAACCCGTT--AGGTTCCCTAAATTTTATGTGCCAAATAAATTTATAAAA-AAAAAAACATAAAGACATGATGCGGGTGGACTCACAAAGTGAAGCATACAAGTCATACAAGTTATAAAGTTTTTGAAAGCGAAATAACTTTTTAAATTTTTTTAAATATTATAATCGGATATTTCTACGACTAACAACTGAACTGAATTAACGGAATAATTAATCAATCATGTATTGATGTAAGCGAATTGGACCACATCAGGGTACTTAGAGTTTGGGGGGCTAGCTAGTGATTAGATCCAAAGTTTTTTTCACCACATATCAATGTGGTCCATCCTAATTAAGTCCATTGTCTACCCCATTTTTGGTGGGGTCCCCAATCTATGAAAAA-TCGAATTCATTTTACTGCTCACTCACCAAAAATGTTCAACAGCCCCACGGTACATTATACAAGTAATAGTAATACAACAACATACATTAAACTTATATAG

>PI207508

AAGTTCAATTATCATCTTCAGGTAAATCGGTAATTGACAAATTTAAATTTCAAATATGTTTTGAATCCTTGTATTTAG-----------GTTTGTCCTAACTCCTAACCCGTT--AGGTTCCCTAAATTTTATGTGCCAAATAAATTTATAAAA-AAAAAAACATAAAGACATGATGCGGGTGGACTCACAAAGTGAAGCATACAAGTCATACAAGTTATAAAGTTTTTGAAAGCGAAATAACTTTTTAAATTTTTTTAAATATTATAATCGGATATTTCTACGACTAACAACTGAACTGAATTAACGGAATAATTAATCAATCATGTATTGATGTAAGCGAATTGGACCACATCAGGGTACTTAGAGTTTGGGGGGCTAGCTAGTGATTAGATCCAAAGTTTTTTTCACCACATATCAATGTGGTCCATCCTAATTAAGTCCATTGTCTACCCCATTTTTGGTGGGGTCCCCAATCTATGAAAAA-TCGAATTCATTTTACTGCTCACTCACCAAAAATGTTCAACAGCCCCACGGTACATTATACAAGTAATAGTAATACAACAACATACATTAAACTTATATAG

>PI220174

AAGTTCAATTATCATCTTCAGGTAAATCGGTAATTGACAAATTTAAATTTCAAATATGTTTTGAATCCTTGTATTTAG-----------GTTTGTCCTAACTCCTAACCCGTT--AGGTTCCCTAAATTTTATGTGCCAAATAAATTTATAAAA-AAAAAAACATAAAGACATGATGCGGGTGGACTCACAAAGTGAAGCATACAAGTCATACAAGTTATAAAGTTTTTGAAAGCGAAATAACTTTTTAAATTTTTTTAAATATTATAATCGGATATTTCTACGACTAACAACTGAACTGAATTAACGGAATAATTAATCAATCATGTATTGATGTAAGCGAATTGGACCACATCAGGGTACTTAGAGTTTGGGGGGCTAGCTAGTGATTAGATCCAAAGTTTTTTTCACCACATATCAATGTGGTCCATCCTAATTAAGTCCATTGTCTACCCCATTTTTGGTGGGGTCCCCAATCTATGAAAAA-TCGAATTCATTTTACTGCTCACTCACCAAAAATGTTCAACAGCCCCACGGTACATTATACAAGTAATAGTAATACAACAACATACATTAAACTTATATAG

>PI220189

AAGTTCAATTATCATCTTCAGGTAAATCGGTAATTGACAAATTTAAATTTCAAATATGTTTTGAATCCTTGTATTTAG-----------GTTTGTCCTAACTCCTAACCCGTT--AGGTTCCCTAAATTTTATGTGCCAAATAAATTTATAAAA-AAAAAAACATAAAGACATGATGCGGGTGGACTCACAAAGTGAAGCATACAAGTCATACAAGTTATAAAGTTTTTGAAAGCGAAATAACTTTTTAAATTTTTTTAAATATTATAATCGGATATTTCTACGACTAACAACTGAACTGAATTAACGGAATAATTAATCAATCATGTATTGATGTAAGCGAATTGGACCACATCAGGGTACTTAGAGTTTGGGGGGCTAGCTAGTGATTAGATCCAAAGTTTTTTTCACCACATATCAATGTGGTCCATCCTAATTAAGTCCATTGTCTACCCCATTTTTGGTGGGGTCCCCAATCTATGAAAAA-TCGAATTCATTTTACTGCTCACTCACCAAAAATGTTCAACAGCCCCACGGTACATTATACAAGTAATAGTAATACAACAACATACATTAAACTTATATAG

>PI222071

AAGTTCAATTATCATCTTCAGGTAAATCGGTAATTGACAAATTTAAATTTCAAATATGTTTTGAATCCTTGTATTTAG-----------GTTTGTCCTAACTCCTAACCCGTT--AGGTTCCCTAAATTTTATGTGCCAAATAAATTTATAAAA-AAAAAAACATAAAGACATGATGCGGGTGGACTCACAAAGTGAAGCATACAAGTCATACAAGTTATAAAGTTTTTGAAAGCGAAATAACTTTTTAAATTTTTTTAAATATTATAATCGGATATTTCTACGACTAACAACTGAACTGAATTAACGGAATAATTAATCAATCATGTATTGATGTAAGCGAATTGGACCACATCAGGGTACTTAGAGTTTGGGGGGCTAGCTAGTGATTAGATCCAAAGTTTTTTTCACCACATATCAATGTGGTCCATCCTAATTAAGTCCATTGTCTACCCCATTTTTGGTGGGGTCCCCAATCTATGAAAAA-TCGAATTCATTTTACTGCTCACTCACCAAAAATGTTCAACAGCCCCACGGTACATTATACAAGTAATAGTAATACAACAACATACATTAAACTTATATAG

>PI222117

AAGTTCAATTATCATCTTCAGGTAAATCGGTAATTGACAAATTTAAATTTCAAATATGTTTTGAATCCTTGTATTTAG-----------GTTTGTCCTAACTCCTAACCCGTT--AGGTTCCCTAAATTTTATGTGCCAAATAAATTTATAAAA-AAAAAAACATAAAGACATGATGCGGGTGGACTCACAAAGTGAAGCATACAAGTCATACAAGTTATAAAGTTTTTGAAAGCGAAATAACTTTTTAAATTTTTTTAAATATTATAATCGGATATTTCTACGACTAACAACTGAACTGAATTAACGGAATAATTAATCAATCATGTATTGATGTAAGCGAATTGGACCACATCAGGGTACTTAGAGTTTGGGGGGCTAGCTAGTGATTAGATCCAAAGTTTTTTTCACCACATATCAATGTGGTCCATCCTAATTAAGTCCATTGTCTACCCCATTTTTGGTGGGGTCCCCAATCTATGAAAAA-TCGAATTCATTTTACTGCTCACTCACCAAAAATGTTCAACAGCCCCACGGTACATTATACAAGTAATAGTAATACAACAACATACATTAAACTTATATAG

>PI340130

AAGTTCAATTATCATCTTCAGGTAAATCGGTAATTGACAAATTTAAATTTCAAATATGTTTTGAATCCTTGTATTTAG-----------GTTTGTCCTAACTCCTAACCCGTT--AGGTTCCCTAAATTTTATGTGCCAAATAAATTTATAAAA-AAAAAAACATAAAGACATGATGCGGGTGGACTCACAAAGTGAAGCATACAAGTCATACAAGTTATAAAGTTTTTGAAAGCGAAATAACTTTTTAAATTTTTTTAAATATTATAATCGGATATTTCTACGACTAACAACTGAACTGAATTAACGGAATAATTAATCAATCATGTATTGATGTAAGCGAATTGGACCACATCAGGGTACTTAGAGTTTGGGGGGCTAGCTAGTGATTAGATCCAAAGTTTTTTTCACCACATATCAATGTGGTCCATCCTAATTAAGTCCATTGTCTACCCCATTTTTGGTGGGGTCCCCAATCTATGAAAAA-TCGAATTCATTTTACTGCTCACTCACCAAAAATGTTCAACAGCCCCACGGTACATTATACAAGTAATAGTAATACAACAACATACATTAAACTTATATAG

>PI210558

AAGTTCAATTATCATCTTCAGATAAATCGGTAATTGACAAATTTAAATTTCAAATATGTTTTGAATCCTTGTATTTAG-----------GTTTGTCCTAACTCCTAACCCGTT--AGGTTCCCTAAATTTTATGTGCCAAATAAATTTATAAAA-AAAAAAACATAAAGACATGATGCGGGTGGACTCACAAAGTGAAGCATACAAGTCATACAAGTTATAAAGTTTTTGAAAGCGAAATAACTTTTTAAATTTTTTTAAATATTATAATCGGATATTTCTACGACTAACAACTGAACTGAATTAACGGAATAATTAATCAATCATGTATTGATGTAAGCGAATTGGACCACATCAGGGTACTTAGAGTTTGGGGGGCTAGCTAGTGATTAGATCCAAAGTTTTTTTCACCACATATCAATGTGGTCCATCCTAATTAAGTCCATTGTCTACCCCATTTTTGGTGGGGTCCCCAATCTATGAAAAA-TCGAATTCATTTTACTGCTCACTCACCAAAAATGTTCAACAGCCCCACGGTACATTATACAAGTAATAGTAATACAACAACATACATTAAACTTATATAG

>PI103058

AAGTTCAATTATCATCTTCAGATAAATCGGTAATTAACAAATTTAAATTTCAAATATGTTTTGAATCCTTGTATTTAG-----------GTTTGTCCTAACTCCTAGCCCTAACGAACTTCACTAAATTTTATGTGCCAAATAAATTTATAAAA-AAAAA--CATAAAGACATGATGCGG-TGGACTCACAAACTGAAGCATAC---------AAGTTATAAAGTTTTTGAAAGCGAAATAACGTCTTAAATTTTTTTAAATATTATAATCGAATATTTCTACGACTAACAACTGAACTGAATTAACGGAATAATTAATCTATCATATATTGATTTAAGCGAATTGGACCACATCAGGGTACATAGAGTTTGGGGGGCTAGCTAGTGATTAGATCCAAAGTTTTTTTCACCACATATCAATGTGGTCCATCCTAATTAAGTCCATTGTCTACCCCATTTTTGGTGGGGTCCCCAATCTATGAAAAA-TCGAATTCATTTTACTGCTCACTCACCAAAAATGTTCAACAGCCCCACGGTACATTATACAAGTAATAGTAATACAACAACATACATTAAACTTATATAG

>PI116844

AAGTTCAATTATCATCTTCAGATAAATCGGTAATTAACAAATTTAAATTTCAAATATGTTTTGAATCCTTGTATTTAG-----------GTTTGTCCTAACTCCTAGCCCTAACGAACTTCACTAAATTTTATGTGCCAAATAAATTTATAAAA-AAAAA--CATAAAGACATGATGCGG-TGGACTCACAAACTGAAGCATAC---------AAGTTATAAAGTTTTTGAAAGCGAAATAACGTCTTAAATTTTTTTAAATATTATAATCGAATATTTCTACGACTAACAACTGAACTGAATTAACGGAATAATTAATCTATCATATATTGATTTAAGCGAATTGGACCACATCAGGGTACATAGAGTTTGGGGGGCTAGCTAGTGATTAGATCCAAAGTTTTTTTCACCACATATCAATGTGGTCCATCCTAATTAAGTCCATTGTCTACCCCATTTTTGGTGGGGTCCCCAATCTATGAAAAA-TCGAATTCATTTTACTGCTCACTCACCAAAAATGTTCAACAGCCCCACGGTACATTATACAAGTAATAGTAATACAACAACATACATTAAACTTATATAG

>PI117264

AAGTTCAATTATCATCTTCAGATAAATCGGTAATTAACAAATTTAAATTTCAAATATGTTTTGAATCCTTGTATTTAG-----------GTTTGTCCTAACTCCTAGCCCTAACGAACTTCACTAAATTTTATGTGCCAAATAAATTTATAAAA-AAAAA--CATAAAGACATGATGCGG-TGGACTCACAAACTGAAGCATAC---------AAGTTATAAAGTTTTTGAAAGCGAAATAACGTCTTAAATTTTTTTAAATATTATAATCGAATATTTCTACGACTAACAACTGAACTGAATTAACGGAATAATTAATCTATCATATATTGATTTAAGCGAATTGGACCACATCAGGGTACATAGAGTTTGGGGGGCTAGCTAGTGATTAGATCCAAAGTTTTTTTCACCACATATCAATGTGGTCCATCCTAATTAAGTCCATTGTCTACCCCATTTTTGGTGGGGTCCCCAATCTATGAAAAA-TCGAATTCATTTTACTGCTCACTCACCAAAAATGTTCAACAGCCCCACGGTACATTATACAAGTAATAGTAATACAACAACATACATTAAACTTATATAG

>PI121352

AAGTTCAATTATCATCTTCAGATAAATCGGTAATTAACAAATTTAAATTTCAAATATGTTTTGAATCCTTGTATTTAG-----------GTTTGTCCTAACTCCTAGCCCTAACGAACTTCACTAAATTTTATGTGCCAAATAAATTTATAAAA-AAAAA--CATAAAGACATGATGCGG-TGGACTCACAAACTGAAGCATAC---------AAGTTATAAAGTTTTTGAAAGCGAAATAACGTCTTAAATTTTTTTAAATATTATAATCGAATATTTCTACGACTAACAACTGAACTGAATTAACGGAATAATTAATCTATCATATATTGATTTAAGCGAATTGGACCACATCAGGGTACATAGAGTTTGGGGGGCTAGCTAGTGATTAGATCCAAAGTTTTTTTCACCACATATCAATGTGGTCCATCCTAATTAAGTCCATTGTCTACCCCATTTTTGGTGGGGTCCCCAATCTATGAAAAA-TCGAATTCATTTTACTGCTCACTCACCAAAAATGTTCAACAGCCCCACGGTACATTATACAAGTAATAGTAATACAACAACATACATTAAACTTATATAG

>PI124478

AAGTTCAATTATCATCTTCAGATAAATCGGTAATTAACAAATTTAAATTTCAAATATGTTTTGAATCCTTGTATTTAG-----------GTTTGTCCTAACTCCTAGCCCTAACGAACTTCACTAAATTTTATGTGCCAAATAAATTTATAAAA-AAAAA--CATAAAGACATGATGCGG-TGGACTCACAAACTGAAGCATAC---------AAGTTATAAAGTTTTTGAAAGCGAAATAACGTCTTAAATTTTTTTAAATATTATAATCGAATATTTCTACGACTAACAACTGAACTGAATTAACGGAATAATTAATCTATCATATATTGATTTAAGCGAATTGGACCACATCAGGGTACATAGAGTTTGGGGGGCTAGCTAGTGATTAGATCCAAAGTTTTTTTCACCACATATCAATGTGGTCCATCCTAATTAAGTCCATTGTCTACCCCATTTTTGGTGGGGTCCCCAATCTATGAAAAA-TCGAATTCATTTTACTGCTCACTCACCAAAAATGTTCAACAGCCCCACGGTACATTATACAAGTAATAGTAATACAACAACATACATTAAACTTATATAG

>PI134271

AAGTTCAATTATCATCTTCAGATAAATCGGTAATTAACAAATTTAAATTTCAAATATGTTTTGAATCCTTGTATTTAG-----------GTTTGTCCTAACTCCTAGCCCTAACGAACTTCACTAAATTTTATGTGCCAAATAAATTTATAAAA-AAAAA--CATAAAGACATGATGCGG-TGGACTCACAAACTGAAGCATAC---------AAGTTATAAAGTTTTTGAAAGCGAAATAACGTCTTAAATTTTTTTAAATATTATAATCGAATATTTCTACGACTAACAACTGAACTGAATTAACGGAATAATTAATCTATCATATATTGATTTAAGCGAATTGGACCACATCAGGGTACATAGAGTTTGGGGGGCTAGCTAGTGATTAGATCCAAAGTTTTTTTCACCACATATCAATGTGGTCCATCCTAATTAAGTCCATTGTCTACCCCATTTTTGGTGGGGTCCCCAATCTATGAAAAA-TCGAATTCATTTTACTGCTCACTCACCAAAAATGTTCAACAGCCCCACGGTACATTATACAAGTAATAGTAATACAACAACATACATTAAACTTATATAG

>PI156720

AAGTTCAATTATCATCTTCAGATAAATCGGTAATTAACAAATTTAAATTTCAAATATGTTTTGAATCCTTGTATTTAG-----------GTTTGTCCTAACTCCTAGCCCTAACGAACTTCACTAAATTTTATGTGCCAAATAAATTTATAAAA-AAAAA--CATAAAGACATGATGCGG-TGGACTCACAAACTGAAGCATAC---------AAGTTATAAAGTTTTTGAAAGCGAAATAACGTCTTAAATTTTTTTAAATATTATAATCGAATATTTCTACGACTAACAACTGAACTGAATTAACGGAATAATTAATCTATCATATATTGATTTAAGCGAATTGGACCACATCAGGGTACATAGAGTTTGGGGGGCTAGCTAGTGATTAGATCCAAAGTTTTTTTCACCACATATCAATGTGGTCCATCCTAATTAAGTCCATTGTCTACCCCATTTTTGGTGGGGTCCCCAATCTATGAAAAA-TCGAATTCATTTTACTGCTCACTCACCAAAAATGTTCAACAGCCCCACGGTACATTATACAAGTAATAGTAATACAACAACATACATTAAACTTATATAG

>PI163126

AAGTTCAATTATCATCTTCAGATAAATCGGTAATTAACAAATTTAAATTTCAAATATGTTTTGAATCCTTGTATTTAG-----------GTTTGTCCTAACTCCTAGCCCTAACGAACTTCACTAAATTTTATGTGCCAAATAAATTTATAAAA-AAAAA--CATAAAGACATGATGCGG-TGGACTCACAAACTGAAGCATAC---------AAGTTATAAAGTTTTTGAAAGCGAAATAACGTCTTAAATTTTTTTAAATATTATAATCGAATATTTCTACGACTAACAACTGAACTGAATTAACGGAATAATTAATCTATCATATATTGATTTAAGCGAATTGGACCACATCAGGGTACATAGAGTTTGGGGGGCTAGCTAGTGATTAGATCCAAAGTTTTTTTCACCACATATCAATGTGGTCCATCCTAATTAAGTCCATTGTCTACCCCATTTTTGGTGGGGTCCCCAATCTATGAAAAA-TCGAATTCATTTTACTGCTCACTCACCAAAAATGTTCAACAGCCCCACGGTACATTATACAAGTAATAGTAATACAACAACATACATTAAACTTATATAG

>PI163129

AAGTTCAATTATCATCTTCAGATAAATCGGTAATTAACAAATTTAAATTTCAAATATGTTTTGAATCCTTGTATTTAG-----------GTTTGTCCTAACTCCTAGCCCTAACGAACTTCACTAAATTTTATGTGCCAAATAAATTTATAAAA-AAAAA--CATAAAGACATGATGCGG-TGGACTCACAAACTGAAGCATAC---------AAGTTATAAAGTTTTTGAAAGCGAAATAACGTCTTAAATTTTTTTAAATATTATAATCGAATATTTCTACGACTAACAACTGAACTGAATTAACGGAATAATTAATCTATCATATATTGATTTAAGCGAATTGGACCACATCAGGGTACATAGAGTTTGGGGGGCTAGCTAGTGATTAGATCCAAAGTTTTTTTCACCACATATCAATGTGGTCCATCCTAATTAAGTCCATTGTCTACCCCATTTTTGGTGGGGTCCCCAATCTATGAAAAA-TCGAATTCATTTTACTGCTCACTCACCAAAAATGTTCAACAGCCCCACGGTACATTATACAAGTAATAGTAATACAACAACATACATTAAACTTATATAG

>PI164182

AAGTTCAATTATCATCTTCAGATAAATCGGTAATTAACAAATTTAAATTTCAAATATGTTTTGAATCCTTGTATTTAG-----------GTTTGTCCTAACTCCTAGCCCTAACGAACTTCACTAAATTTTATGTGCCAAATAAATTTATAAAA-AAAAA--CATAAAGACATGATGCGG-TGGACTCACAAACTGAAGCATAC---------AAGTTATAAAGTTTTTGAAAGCGAAATAACGTCTTAAATTTTTTTAAATATTATAATCGAATATTTCTACGACTAACAACTGAACTGAATTAACGGAATAATTAATCTATCATATATTGATTTAAGCGAATTGGACCACATCAGGGTACATAGAGTTTGGGGGGCTAGCTAGTGATTAGATCCAAAGTTTTTTTCACCACATATCAATGTGGTCCATCCTAATTAAGTCCATTGTCTACCCCATTTTTGGTGGGGTCCCCAATCTATGAAAAA-TCGAATTCATTTTACTGCTCACTCACCAAAAATGTTCAACAGCCCCACGGTACATTATACAAGTAATAGTAATACAACAACATACATTAAACTTATATAG

>PI164548

AAGTTCAATTATCATCTTCAGATAAATCGGTAATTAACAAATTTAAATTTCAAATATGTTTTGAATCCTTGTATTTAG-----------GTTTGTCCTAACTCCTAGCCCTAACGAACTTCACTAAATTTTATGTGCCAAATAAATTTATAAAA-AAAAA--CATAAAGACATGATGCGG-TGGACTCACAAACTGAAGCATAC---------AAGTTATAAAGTTTTTGAAAGCGAAATAACGTCTTAAATTTTTTTAAATATTATAATCGAATATTTCTACGACTAACAACTGAACTGAATTAACGGAATAATTAATCTATCATATATTGATTTAAGCGAATTGGACCACATCAGGGTACATAGAGTTTGGGGGGCTAGCTAGTGATTAGATCCAAAGTTTTTTTCACCACATATCAATGTGGTCCATCCTAATTAAGTCCATTGTCTACCCCATTTTTGGTGGGGTCCCCAATCTATGAAAAA-TCGAATTCATTTTACTGCTCACTCACCAAAAATGTTCAACAGCCCCACGGTACATTATACAAGTAATAGTAATACAACAACATACATTAAACTTATATAG

>PI164612

AAGTTCAATTATCATCTTCAGATAAATCGGTAATTAACAAATTTAAATTTCAAATATGTTTTGAATCCTTGTATTTAG-----------GTTTGTCCTAACTCCTAGCCCTAACGAACTTCACTAAATTTTATGTGCCAAATAAATTTATAAAA-AAAAA--CATAAAGACATGATGCGG-TGGACTCACAAACTGAAGCATAC---------AAGTTATAAAGTTTTTGAAAGCGAAATAACGTCTTAAATTTTTTTAAATATTATAATCGAATATTTCTACGACTAACAACTGAACTGAATTAACGGAATAATTAATCTATCATATATTGATTTAAGCGAATTGGACCACATCAGGGTACATAGAGTTTGGGGGGCTAGCTAGTGATTAGATCCAAAGTTTTTTTCACCACATATCAATGTGGTCCATCCTAATTAAGTCCATTGTCTACCCCATTTTTGGTGGGGTCCCCAATCTATGAAAAA-TCGAATTCATTTTACTGCTCACTCACCAAAAATGTTCAACAGCCCCACGGTACATTATACAAGTAATAGTAATACAACAACATACATTAAACTTATATAG

>PI169603

AAGTTCAATTATCATCTTCAGATAAATCGGTAATTAACAAATTTAAATTTCAAATATGTTTTGAATCCTTGTATTTAG-----------GTTTGTCCTAACTCCTAGCCCTAACGAACTTCACTAAATTTTATGTGCCAAATAAATTTATAAAA-AAAAA--CATAAAGACATGATGCGG-TGGACTCACAAACTGAAGCATAC---------AAGTTATAAAGTTTTTGAAAGCGAAATAACGTCTTAAATTTTTTTAAATATTATAATCGAATATTTCTACGACTAACAACTGAACTGAATTAACGGAATAATTAATCTATCATATATTGATTTAAGCGAATTGGACCACATCAGGGTACATAGAGTTTGGGGGGCTAGCTAGTGATTAGATCCAAAGTTTTTTTCACCACATATCAATGTGGTCCATCCTAATTAAGTCCATTGTCTACCCCATTTTTGGTGGGGTCCCCAATCTATGAAAAA-TCGAATTCATTTTACTGCTCACTCACCAAAAATGTTCAACAGCCCCACGGTACATTATACAAGTAATAGTAATACAACAACATACATTAAACTTATATAG

>PI171810

AAGTTCAATTATCATCTTCAGATAAATCGGTAATTAACAAATTTAAATTTCAAATATGTTTTGAATCCTTGTATTTAG-----------GTTTGTCCTAACTCCTAGCCCTAACGAACTTCACTAAATTTTATGTGCCAAATAAATTTATAAAA-AAAAA--CATAAAGACATGATGCGG-TGGACTCACAAACTGAAGCATAC---------AAGTTATAAAGTTTTTGAAAGCGAAATAACGTCTTAAATTTTTTTAAATATTATAATCGAATATTTCTACGACTAACAACTGAACTGAATTAACGGAATAATTAATCTATCATATATTGATTTAAGCGAATTGGACCACATCAGGGTACATAGAGTTTGGGGGGCTAGCTAGTGATTAGATCCAAAGTTTTTTTCACCACATATCAATGTGGTCCATCCTAATTAAGTCCATTGTCTACCCCATTTTTGGTGGGGTCCCCAATCTATGAAAAA-TCGAATTCATTTTACTGCTCACTCACCAAAAATGTTCAACAGCCCCACGGTACATTATACAAGTAATAGTAATACAACAACATACATTAAACTTATATAG

>PI172339

AAGTTCAATTATCATCTTCAGATAAATCGGTAATTAACAAATTTAAATTTCAAATATGTTTTGAATCCTTGTATTTAG-----------GTTTGTCCTAACTCCTAGCCCTAACGAACTTCACTAAATTTTATGTGCCAAATAAATTTATAAAA-AAAAA--CATAAAGACATGATGCGG-TGGACTCACAAACTGAAGCATAC---------AAGTTATAAAGTTTTTGAAAGCGAAATAACGTCTTAAATTTTTTTAAATATTATAATCGAATATTTCTACGACTAACAACTGAACTGAATTAACGGAATAATTAATCTATCATATATTGATTTAAGCGAATTGGACCACATCAGGGTACATAGAGTTTGGGGGGCTAGCTAGTGATTAGATCCAAAGTTTTTTTCACCACATATCAATGTGGTCCATCCTAATTAAGTCCATTGTCTACCCCATTTTTGGTGGGGTCCCCAATCTATGAAAAA-TCGAATTCATTTTACTGCTCACTCACCAAAAATGTTCAACAGCCCCACGGTACATTATACAAGTAATAGTAATACAACAACATACATTAAACTTATATAG

>PI173840

AAGTTCAATTATCATCTTCAGATAAATCGGTAATTAACAAATTTAAATTTCAAATATGTTTTGAATCCTTGTATTTAG-----------GTTTGTCCTAACTCCTAGCCCTAACGAACTTCACTAAATTTTATGTGCCAAATAAATTTATAAAA-AAAAA--CATAAAGACATGATGCGG-TGGACTCACAAACTGAAGCATAC---------AAGTTATAAAGTTTTTGAAAGCGAAATAACGTCTTAAATTTTTTTAAATATTATAATCGAATATTTCTACGACTAACAACTGAACTGAATTAACGGAATAATTAATCTATCATATATTGATTTAAGCGAATTGGACCACATCAGGGTACATAGAGTTTGGGGGGCTAGCTAGTGATTAGATCCAAAGTTTTTTTCACCACATATCAATGTGGTCCATCCTAATTAAGTCCATTGTCTACCCCATTTTTGGTGGGGTCCCCAATCTATGAAAAA-TCGAATTCATTTTACTGCTCACTCACCAAAAATGTTCAACAGCCCCACGGTACATTATACAAGTAATAGTAATACAACAACATACATTAAACTTATATAG

>PI179459

AAGTTCAATTATCATCTTCAGATAAATCGGTAATTAACAAATTTAAATTTCAAATATGTTTTGAATCCTTGTATTTAG-----------GTTTGTCCTAACTCCTAGCCCTAACGAACTTCACTAAATTTTATGTGCCAAATAAATTTATAAAA-AAAAA--CATAAAGACATGATGCGG-TGGACTCACAAACTGAAGCATAC---------AAGTTATAAAGTTTTTGAAAGCGAAATAACGTCTTAAATTTTTTTAAATATTATAATCGAATATTTCTACGACTAACAACTGAACTGAATTAACGGAATAATTAATCTATCATATATTGATTTAAGCGAATTGGACCACATCAGGGTACATAGAGTTTGGGGGGCTAGCTAGTGATTAGATCCAAAGTTTTTTTCACCACATATCAATGTGGTCCATCCTAATTAAGTCCATTGTCTACCCCATTTTTGGTGGGGTCCCCAATCTATGAAAAA-TCGAATTCATTTTACTGCTCACTCACCAAAAATGTTCAACAGCCCCACGGTACATTATACAAGTAATAGTAATACAACAACATACATTAAACTTATATAG

>PI179970

AAGTTCAATTATCATCTTCAGATAAATCGGTAATTAACAAATTTAAATTTCAAATATGTTTTGAATCCTTGTATTTAG-----------GTTTGTCCTAACTCCTAGCCCTAACGAACTTCACTAAATTTTATGTGCCAAATAAATTTATAAAA-AAAAA--CATAAAGACATGATGCGG-TGGACTCACAAACTGAAGCATAC---------AAGTTATAAAGTTTTTGAAAGCGAAATAACGTCTTAAATTTTTTTAAATATTATAATCGAATATTTCTACGACTAACAACTGAACTGAATTAACGGAATAATTAATCTATCATATATTGATTTAAGCGAATTGGACCACATCAGGGTACATAGAGTTTGGGGGGCTAGCTAGTGATTAGATCCAAAGTTTTTTTCACCACATATCAATGTGGTCCATCCTAATTAAGTCCATTGTCTACCCCATTTTTGGTGGGGTCCCCAATCTATGAAAAA-TCGAATTCATTTTACTGCTCACTCACCAAAAATGTTCAACAGCCCCACGGTACATTATACAAGTAATAGTAATACAACAACATACATTAAACTTATATAG

>PI180696

AAGTTCAATTATCATCTTCAGATAAATCGGTAATTAACAAATTTAAATTTCAAATATGTTTTGAATCCTTGTATTTAG-----------GTTTGTCCTAACTCCTAGCCCTAACGAACTTCACTAAATTTTATGTGCCAAATAAATTTATAAAA-AAAAA--CATAAAGACATGATGCGG-TGGACTCACAAACTGAAGCATAC---------AAGTTATAAAGTTTTTGAAAGCGAAATAACGTCTTAAATTTTTTTAAATATTATAATCGAATATTTCTACGACTAACAACTGAACTGAATTAACGGAATAATTAATCTATCATATATTGATTTAAGCGAATTGGACCACATCAGGGTACATAGAGTTTGGGGGGCTAGCTAGTGATTAGATCCAAAGTTTTTTTCACCACATATCAATGTGGTCCATCCTAATTAAGTCCATTGTCTACCCCATTTTTGGTGGGGTCCCCAATCTATGAAAAA-TCGAATTCATTTTACTGCTCACTCACCAAAAATGTTCAACAGCCCCACGGTACATTATACAAGTAATAGTAATACAACAACATACATTAAACTTATATAG

>PI181799

AAGTTCAATTATCATCTTCAGATAAATCGGTAATTAACAAATTTAAATTTCAAATATGTTTTGAATCCTTGTATTTAG-----------GTTTGTCCTAACTCCTAGCCCTAACGAACTTCACTAAATTTTATGTGCCAAATAAATTTATAAAA-AAAAA--CATAAAGACATGATGCGG-TGGACTCACAAACTGAAGCATAC---------AAGTTATAAAGTTTTTGAAAGCGAAATAACGTCTTAAATTTTTTTAAATATTATAATCGAATATTTCTACGACTAACAACTGAACTGAATTAACGGAATAATTAATCTATCATATATTGATTTAAGCGAATTGGACCACATCAGGGTACATAGAGTTTGGGGGGCTAGCTAGTGATTAGATCCAAAGTTTTTTTCACCACATATCAATGTGGTCCATCCTAATTAAGTCCATTGTCTACCCCATTTTTGGTGGGGTCCCCAATCTATGAAAAA-TCGAATTCATTTTACTGCTCACTCACCAAAAATGTTCAACAGCCCCACGGTACATTATACAAGTAATAGTAATACAACAACATACATTAAACTTATATAG

>PI181801

AAGTTCAATTATCATCTTCAGATAAATCGGTAATTAACAAATTTAAATTTCAAATATGTTTTGAATCCTTGTATTTAG-----------GTTTGTCCTAACTCCTAGCCCTAACGAACTTCACTAAATTTTATGTGCCAAATAAATTTATAAAA-AAAAA--CATAAAGACATGATGCGG-TGGACTCACAAACTGAAGCATAC---------AAGTTATAAAGTTTTTGAAAGCGAAATAACGTCTTAAATTTTTTTAAATATTATAATCGAATATTTCTACGACTAACAACTGAACTGAATTAACGGAATAATTAATCTATCATATATTGATTTAAGCGAATTGGACCACATCAGGGTACATAGAGTTTGGGGGGCTAGCTAGTGATTAGATCCAAAGTTTTTTTCACCACATATCAATGTGGTCCATCCTAATTAAGTCCATTGTCTACCCCATTTTTGGTGGGGTCCCCAATCTATGAAAAA-TCGAATTCATTTTACTGCTCACTCACCAAAAATGTTCAACAGCCCCACGGTACATTATACAAGTAATAGTAATACAACAACATACATTAAACTTATATAG

>PI181958

AAGTTCAATTATCATCTTCAGATAAATCGGTAATTAACAAATTTAAATTTCAAATATGTTTTGAATCCTTGTATTTAG-----------GTTTGTCCTAACTCCTAGCCCTAACGAACTTCACTAAATTTTATGTGCCAAATAAATTTATAAAA-AAAAA--CATAAAGACATGATGCGG-TGGACTCACAAACTGAAGCATAC---------AAGTTATAAAGTTTTTGAAAGCGAAATAACGTCTTAAATTTTTTTAAATATTATAATCGAATATTTCTACGACTAACAACTGAACTGAATTAACGGAATAATTAATCTATCATATATTGATTTAAGCGAATTGGACCACATCAGGGTACATAGAGTTTGGGGGGCTAGCTAGTGATTAGATCCAAAGTTTTTTTCACCACATATCAATGTGGTCCATCCTAATTAAGTCCATTGTCTACCCCATTTTTGGTGGGGTCCCCAATCTATGAAAAA-TCGAATTCATTTTACTGCTCACTCACCAAAAATGTTCAACAGCCCCACGGTACATTATACAAGTAATAGTAATACAACAACATACATTAAACTTATATAG

>PI184130

AAGTTCAATTATCATCTTCAGATAAATCGGTAATTAACAAATTTAAATTTCAAATATGTTTTGAATCCTTGTATTTAG-----------GTTTGTCCTAACTCCTAGCCCTAACGAACTTCACTAAATTTTATGTGCCAAATAAATTTATAAAA-AAAAA--CATAAAGACATGATGCGG-TGGACTCACAAACTGAAGCATAC---------AAGTTATAAAGTTTTTGAAAGCGAAATAACGTCTTAAATTTTTTTAAATATTATAATCGAATATTTCTACGACTAACAACTGAACTGAATTAACGGAATAATTAATCTATCATATATTGATTTAAGCGAATTGGACCACATCAGGGTACATAGAGTTTGGGGGGCTAGCTAGTGATTAGATCCAAAGTTTTTTTCACCACATATCAATGTGGTCCATCCTAATTAAGTCCATTGTCTACCCCATTTTTGGTGGGGTCCCCAATCTATGAAAAA-TCGAATTCATTTTACTGCTCACTCACCAAAAATGTTCAACAGCCCCACGGTACATTATACAAGTAATAGTAATACAACAACATACATTAAACTTATATAG

>PI193584

AAGTTCAATTATCATCTTCAGATAAATCGGTAATTAACAAATTTAAATTTCAAATATGTTTTGAATCCTTGTATTTAG-----------GTTTGTCCTAACTCCTAGCCCTAACGAACTTCACTAAATTTTATGTGCCAAATAAATTTATAAAA-AAAAA--CATAAAGACATGATGCGG-TGGACTCACAAACTGAAGCATAC---------AAGTTATAAAGTTTTTGAAAGCGAAATAACGTCTTAAATTTTTTTAAATATTATAATCGAATATTTCTACGACTAACAACTGAACTGAATTAACGGAATAATTAATCTATCATATATTGATTTAAGCGAATTGGACCACATCAGGGTACATAGAGTTTGGGGGGCTAGCTAGTGATTAGATCCAAAGTTTTTTTCACCACATATCAATGTGGTCCATCCTAATTAAGTCCATTGTCTACCCCATTTTTGGTGGGGTCCCCAATCTATGAAAAA-TCGAATTCATTTTACTGCTCACTCACCAAAAATGTTCAACAGCCCCACGGTACATTATACAAGTAATAGTAATACAACAACATACATTAAACTTATATAG

>PI195404

AAGTTCAATTATCATCTTCAGATAAATCGGTAATTAACAAATTTAAATTTCAAATATGTTTTGAATCCTTGTATTTAG-----------GTTTGTCCTAACTCCTAGCCCTAACGAACTTCACTAAATTTTATGTGCCAAATAAATTTATAAAA-AAAAA--CATAAAGACATGATGCGG-TGGACTCACAAACTGAAGCATAC---------AAGTTATAAAGTTTTTGAAAGCGAAATAACGTCTTAAATTTTTTTAAATATTATAATCGAATATTTCTACGACTAACAACTGAACTGAATTAACGGAATAATTAATCTATCATATATTGATTTAAGCGAATTGGACCACATCAGGGTACATAGAGTTTGGGGGGCTAGCTAGTGATTAGATCCAAAGTTTTTTTCACCACATATCAATGTGGTCCATCCTAATTAAGTCCATTGTCTACCCCATTTTTGGTGGGGTCCCCAATCTATGAAAAA-TCGAATTCATTTTACTGCTCACTCACCAAAAATGTTCAACAGCCCCACGGTACATTATACAAGTAATAGTAATACAACAACATACATTAAACTTATATAG

>PI197044

AAGTTCAATTATCATCTTCAGATAAATCGGTAATTAACAAATTTAAATTTCAAATATGTTTTGAATCCTTGTATTTAG-----------GTTTGTCCTAACTCCTAGCCCTAACGAACTTCACTAAATTTTATGTGCCAAATAAATTTATAAAA-AAAAA--CATAAAGACATGATGCGG-TGGACTCACAAACTGAAGCATAC---------AAGTTATAAAGTTTTTGAAAGCGAAATAACGTCTTAAATTTTTTTAAATATTATAATCGAATATTTCTACGACTAACAACTGAACTGAATTAACGGAATAATTAATCTATCATATATTGATTTAAGCGAATTGGACCACATCAGGGTACATAGAGTTTGGGGGGCTAGCTAGTGATTAGATCCAAAGTTTTTTTCACCACATATCAATGTGGTCCATCCTAATTAAGTCCATTGTCTACCCCATTTTTGGTGGGGTCCCCAATCTATGAAAAA-TCGAATTCATTTTACTGCTCACTCACCAAAAATGTTCAACAGCCCCACGGTACATTATACAAGTAATAGTAATACAACAACATACATTAAACTTATATAG

>PI201390

AAGTTCAATTATCATCTTCAGATAAATCGGTAATTAACAAATTTAAATTTCAAATATGTTTTGAATCCTTGTATTTAG-----------GTTTGTCCTAACTCCTAGCCCTAACGAACTTCACTAAATTTTATGTGCCAAATAAATTTATAAAA-AAAAA--CATAAAGACATGATGCGG-TGGACTCACAAACTGAAGCATAC---------AAGTTATAAAGTTTTTGAAAGCGAAATAACGTCTTAAATTTTTTTAAATATTATAATCGAATATTTCTACGACTAACAACTGAACTGAATTAACGGAATAATTAATCTATCATATATTGATTTAAGCGAATTGGACCACATCAGGGTACATAGAGTTTGGGGGGCTAGCTAGTGATTAGATCCAAAGTTTTTTTCACCACATATCAATGTGGTCCATCCTAATTAAGTCCATTGTCTACCCCATTTTTGGTGGGGTCCCCAATCTATGAAAAA-TCGAATTCATTTTACTGCTCACTCACCAAAAATGTTCAACAGCCCCACGGTACATTATACAAGTAATAGTAATACAACAACATACATTAAACTTATATAG

>PI203066

AAGTTCAATTATCATCTTCAGATAAATCGGTAATTAACAAATTTAAATTTCAAATATGTTTTGAATCCTTGTATTTAG-----------GTTTGTCCTAACTCCTAGCCCTAACGAACTTCACTAAATTTTATGTGCCAAATAAATTTATAAAA-AAAAA--CATAAAGACATGATGCGG-TGGACTCACAAACTGAAGCATAC---------AAGTTATAAAGTTTTTGAAAGCGAAATAACGTCTTAAATTTTTTTAAATATTATAATCGAATATTTCTACGACTAACAACTGAACTGAATTAACGGAATAATTAATCTATCATATATTGATTTAAGCGAATTGGACCACATCAGGGTACATAGAGTTTGGGGGGCTAGCTAGTGATTAGATCCAAAGTTTTTTTCACCACATATCAATGTGGTCCATCCTAATTAAGTCCATTGTCTACCCCATTTTTGGTGGGGTCCCCAATCTATGAAAAA-TCGAATTCATTTTACTGCTCACTCACCAAAAATGTTCAACAGCCCCACGGTACATTATACAAGTAATAGTAATACAACAACATACATTAAACTTATATAG

>PI203067

AAGTTCAATTATCATCTTCAGATAAATCGGTAATTAACAAATTTAAATTTCAAATATGTTTTGAATCCTTGTATTTAG-----------GTTTGTCCTAACTCCTAGCCCTAACGAACTTCACTAAATTTTATGTGCCAAATAAATTTATAAAA-AAAAA--CATAAAGACATGATGCGG-TGGACTCACAAACTGAAGCATAC---------AAGTTATAAAGTTTTTGAAAGCGAAATAACGTCTTAAATTTTTTTAAATATTATAATCGAATATTTCTACGACTAACAACTGAACTGAATTAACGGAATAATTAATCTATCATATATTGATTTAAGCGAATTGGACCACATCAGGGTACATAGAGTTTGGGGGGCTAGCTAGTGATTAGATCCAAAGTTTTTTTCACCACATATCAATGTGGTCCATCCTAATTAAGTCCATTGTCTACCCCATTTTTGGTGGGGTCCCCAATCTATGAAAAA-TCGAATTCATTTTACTGCTCACTCACCAAAAATGTTCAACAGCCCCACGGTACATTATACAAGTAATAGTAATACAACAACATACATTAAACTTATATAG

>PI203068

AAGTTCAATTATCATCTTCAGATAAATCGGTAATTAACAAATTTAAATTTCAAATATGTTTTGAATCCTTGTATTTAG-----------GTTTGTCCTAACTCCTAGCCCTAACGAACTTCACTAAATTTTATGTGCCAAATAAATTTATAAAA-AAAAA--CATAAAGACATGATGCGG-TGGACTCACAAACTGAAGCATAC---------AAGTTATAAAGTTTTTGAAAGCGAAATAACGTCTTAAATTTTTTTAAATATTATAATCGAATATTTCTACGACTAACAACTGAACTGAATTAACGGAATAATTAATCTATCATATATTGATTTAAGCGAATTGGACCACATCAGGGTACATAGAGTTTGGGGGGCTAGCTAGTGATTAGATCCAAAGTTTTTTTCACCACATATCAATGTGGTCCATCCTAATTAAGTCCATTGTCTACCCCATTTTTGGTGGGGTCCCCAATCTATGAAAAA-TCGAATTCATTTTACTGCTCACTCACCAAAAATGTTCAACAGCCCCACGGTACATTATACAAGTAATAGTAATACAACAACATACATTAAACTTATATAG

>PI203069

AAGTTCAATTATCATCTTCAGATAAATCGGTAATTAACAAATTTAAATTTCAAATATGTTTTGAATCCTTGTATTTAG-----------GTTTGTCCTAACTCCTAGCCCTAACGAACTTCACTAAATTTTATGTGCCAAATAAATTTATAAAA-AAAAA--CATAAAGACATGATGCGG-TGGACTCACAAACTGAAGCATAC---------AAGTTATAAAGTTTTTGAAAGCGAAATAACGTCTTAAATTTTTTTAAATATTATAATCGAATATTTCTACGACTAACAACTGAACTGAATTAACGGAATAATTAATCTATCATATATTGATTTAAGCGAATTGGACCACATCAGGGTACATAGAGTTTGGGGGGCTAGCTAGTGATTAGATCCAAAGTTTTTTTCACCACATATCAATGTGGTCCATCCTAATTAAGTCCATTGTCTACCCCATTTTTGGTGGGGTCCCCAATCTATGAAAAA-TCGAATTCATTTTACTGCTCACTCACCAAAAATGTTCAACAGCCCCACGGTACATTATACAAGTAATAGTAATACAACAACATACATTAAACTTATATAG

>PI206838

AAGTTCAATTATCATCTTCAGATAAATCGGTAATTAACAAATTTAAATTTCAAATATGTTTTGAATCCTTGTATTTAG-----------GTTTGTCCTAACTCCTAGCCCTAACGAACTTCACTAAATTTTATGTGCCAAATAAATTTATAAAA-AAAAA--CATAAAGACATGATGCGG-TGGACTCACAAACTGAAGCATAC---------AAGTTATAAAGTTTTTGAAAGCGAAATAACGTCTTAAATTTTTTTAAATATTATAATCGAATATTTCTACGACTAACAACTGAACTGAATTAACGGAATAATTAATCTATCATATATTGATTTAAGCGAATTGGACCACATCAGGGTACATAGAGTTTGGGGGGCTAGCTAGTGATTAGATCCAAAGTTTTTTTCACCACATATCAATGTGGTCCATCCTAATTAAGTCCATTGTCTACCCCATTTTTGGTGGGGTCCCCAATCTATGAAAAA-TCGAATTCATTTTACTGCTCACTCACCAAAAATGTTCAACAGCCCCACGGTACATTATACAAGTAATAGTAATACAACAACATACATTAAACTTATATAG

>PI209507

AAGTTCAATTATCATCTTCAGATAAATCGGTAATTAACAAATTTAAATTTCAAATATGTTTTGAATCCTTGTATTTAG-----------GTTTGTCCTAACTCCTAGCCCTAACGAACTTCACTAAATTTTATGTGCCAAATAAATTTATAAAA-AAAAA--CATAAAGACATGATGCGG-TGGACTCACAAACTGAAGCATAC---------AAGTTATAAAGTTTTTGAAAGCGAAATAACGTCTTAAATTTTTTTAAATATTATAATCGAATATTTCTACGACTAACAACTGAACTGAATTAACGGAATAATTAATCTATCATATATTGATTTAAGCGAATTGGACCACATCAGGGTACATAGAGTTTGGGGGGCTAGCTAGTGATTAGATCCAAAGTTTTTTTCACCACATATCAATGTGGTCCATCCTAATTAAGTCCATTGTCTACCCCATTTTTGGTGGGGTCCCCAATCTATGAAAAA-TCGAATTCATTTTACTGCTCACTCACCAAAAATGTTCAACAGCCCCACGGTACATTATACAAGTAATAGTAATACAACAACATACATTAAACTTATATAG

>PI210561

AAGTTCAATTATCATCTTCAGATAAATCGGTAATTAACAAATTTAAATTTCAAATATGTTTTGAATCCTTGTATTTAG-----------GTTTGTCCTAACTCCTAGCCCTAACGAACTTCACTAAATTTTATGTGCCAAATAAATTTATAAAA-AAAAA--CATAAAGACATGATGCGG-TGGACTCACAAACTGAAGCATAC---------AAGTTATAAAGTTTTTGAAAGCGAAATAACGTCTTAAATTTTTTTAAATATTATAATCGAATATTTCTACGACTAACAACTGAACTGAATTAACGGAATAATTAATCTATCATATATTGATTTAAGCGAATTGGACCACATCAGGGTACATAGAGTTTGGGGGGCTAGCTAGTGATTAGATCCAAAGTTTTTTTCACCACATATCAATGTGGTCCATCCTAATTAAGTCCATTGTCTACCCCATTTTTGGTGGGGTCCCCAATCTATGAAAAA-TCGAATTCATTTTACTGCTCACTCACCAAAAATGTTCAACAGCCCCACGGTACATTATACAAGTAATAGTAATACAACAACATACATTAAACTTATATAG

>PI210568

AAGTTCAATTATCATCTTCAGATAAATCGGTAATTAACAAATTTAAATTTCAAATATGTTTTGAATCCTTGTATTTAG-----------GTTTGTCCTAACTCCTAGCCCTAACGAACTTCACTAAATTTTATGTGCCAAATAAATTTATAAAA-AAAAA--CATAAAGACATGATGCGG-TGGACTCACAAACTGAAGCATAC---------AAGTTATAAAGTTTTTGAAAGCGAAATAACGTCTTAAATTTTTTTAAATATTATAATCGAATATTTCTACGACTAACAACTGAACTGAATTAACGGAATAATTAATCTATCATATATTGATTTAAGCGAATTGGACCACATCAGGGTACATAGAGTTTGGGGGGCTAGCTAGTGATTAGATCCAAAGTTTTTTTCACCACATATCAATGTGGTCCATCCTAATTAAGTCCATTGTCTACCCCATTTTTGGTGGGGTCCCCAATCTATGAAAAA-TCGAATTCATTTTACTGCTCACTCACCAAAAATGTTCAACAGCCCCACGGTACATTATACAAGTAATAGTAATACAACAACATACATTAAACTTATATAG

>PI210569

AAGTTCAATTATCATCTTCAGATAAATCGGTAATTAACAAATTTAAATTTCAAATATGTTTTGAATCCTTGTATTTAG-----------GTTTGTCCTAACTCCTAGCCCTAACGAACTTCACTAAATTTTATGTGCCAAATAAATTTATAAAA-AAAAA--CATAAAGACATGATGCGG-TGGACTCACAAACTGAAGCATAC---------AAGTTATAAAGTTTTTGAAAGCGAAATAACGTCTTAAATTTTTTTAAATATTATAATCGAATATTTCTACGACTAACAACTGAACTGAATTAACGGAATAATTAATCTATCATATATTGATTTAAGCGAATTGGACCACATCAGGGTACATAGAGTTTGGGGGGCTAGCTAGTGATTAGATCCAAAGTTTTTTTCACCACATATCAATGTGGTCCATCCTAATTAAGTCCATTGTCTACCCCATTTTTGGTGGGGTCCCCAATCTATGAAAAA-TCGAATTCATTTTACTGCTCACTCACCAAAAATGTTCAACAGCCCCACGGTACATTATACAAGTAATAGTAATACAACAACATACATTAAACTTATATAG

>PI210571

AAGTTCAATTATCATCTTCAGATAAATCGGTAATTAACAAATTTAAATTTCAAATATGTTTTGAATCCTTGTATTTAG-----------GTTTGTCCTAACTCCTAGCCCTAACGAACTTCACTAAATTTTATGTGCCAAATAAATTTATAAAA-AAAAA--CATAAAGACATGATGCGG-TGGACTCACAAACTGAAGCATAC---------AAGTTATAAAGTTTTTGAAAGCGAAATAACGTCTTAAATTTTTTTAAATATTATAATCGAATATTTCTACGACTAACAACTGAACTGAATTAACGGAATAATTAATCTATCATATATTGATTTAAGCGAATTGGACCACATCAGGGTACATAGAGTTTGGGGGGCTAGCTAGTGATTAGATCCAAAGTTTTTTTCACCACATATCAATGTGGTCCATCCTAATTAAGTCCATTGTCTACCCCATTTTTGGTGGGGTCCCCAATCTATGAAAAA-TCGAATTCATTTTACTGCTCACTCACCAAAAATGTTCAACAGCCCCACGGTACATTATACAAGTAATAGTAATACAACAACATACATTAAACTTATATAG

>PI210583

AAGTTCAATTATCATCTTCAGATAAATCGGTAATTAACAAATTTAAATTTCAAATATGTTTTGAATCCTTGTATTTAG-----------GTTTGTCCTAACTCCTAGCCCTAACGAACTTCACTAAATTTTATGTGCCAAATAAATTTATAAAA-AAAAA--CATAAAGACATGATGCGG-TGGACTCACAAACTGAAGCATAC---------AAGTTATAAAGTTTTTGAAAGCGAAATAACGTCTTAAATTTTTTTAAATATTATAATCGAATATTTCTACGACTAACAACTGAACTGAATTAACGGAATAATTAATCTATCATATATTGATTTAAGCGAATTGGACCACATCAGGGTACATAGAGTTTGGGGGGCTAGCTAGTGATTAGATCCAAAGTTTTTTTCACCACATATCAATGTGGTCCATCCTAATTAAGTCCATTGTCTACCCCATTTTTGGTGGGGTCCCCAATCTATGAAAAA-TCGAATTCATTTTACTGCTCACTCACCAAAAATGTTCAACAGCCCCACGGTACATTATACAAGTAATAGTAATACAACAACATACATTAAACTTATATAG

>PI212031

AAGTTCAATTATCATCTTCAGATAAATCGGTAATTAACAAATTTAAATTTCAAATATGTTTTGAATCCTTGTATTTAG-----------GTTTGTCCTAACTCCTAGCCCTAACGAACTTCACTAAATTTTATGTGCCAAATAAATTTATAAAA-AAAAA--CATAAAGACATGATGCGG-TGGACTCACAAACTGAAGCATAC---------AAGTTATAAAGTTTTTGAAAGCGAAATAACGTCTTAAATTTTTTTAAATATTATAATCGAATATTTCTACGACTAACAACTGAACTGAATTAACGGAATAATTAATCTATCATATATTGATTTAAGCGAATTGGACCACATCAGGGTACATAGAGTTTGGGGGGCTAGCTAGTGATTAGATCCAAAGTTTTTTTCACCACATATCAATGTGGTCCATCCTAATTAAGTCCATTGTCTACCCCATTTTTGGTGGGGTCCCCAATCTATGAAAAA-TCGAATTCATTTTACTGCTCACTCACCAAAAATGTTCAACAGCCCCACGGTACATTATACAAGTAATAGTAATACAACAACATACATTAAACTTATATAG

>PI212917

AAGTTCAATTATCATCTTCAGATAAATCGGTAATTAACAAATTTAAATTTCAAATATGTTTTGAATCCTTGTATTTAG-----------GTTTGTCCTAACTCCTAGCCCTAACGAACTTCACTAAATTTTATGTGCCAAATAAATTTATAAAA-AAAAA--CATAAAGACATGATGCGG-TGGACTCACAAACTGAAGCATAC---------AAGTTATAAAGTTTTTGAAAGCGAAATAACGTCTTAAATTTTTTTAAATATTATAATCGAATATTTCTACGACTAACAACTGAACTGAATTAACGGAATAATTAATCTATCATATATTGATTTAAGCGAATTGGACCACATCAGGGTACATAGAGTTTGGGGGGCTAGCTAGTGATTAGATCCAAAGTTTTTTTCACCACATATCAATGTGGTCCATCCTAATTAAGTCCATTGTCTACCCCATTTTTGGTGGGGTCCCCAATCTATGAAAAA-TCGAATTCATTTTACTGCTCACTCACCAAAAATGTTCAACAGCCCCACGGTACATTATACAAGTAATAGTAATACAACAACATACATTAAACTTATATAG

>PI221697

AAGTTCAATTATCATCTTCAGATAAATCGGTAATTAACAAATTTAAATTTCAAATATGTTTTGAATCCTTGTATTTAG-----------GTTTGTCCTAACTCCTAGCCCTAACGAACTTCACTAAATTTTATGTGCCAAATAAATTTATAAAA-AAAAA--CATAAAGACATGATGCGG-TGGACTCACAAACTGAAGCATAC---------AAGTTATAAAGTTTTTGAAAGCGAAATAACGTCTTAAATTTTTTTAAATATTATAATCGAATATTTCTACGACTAACAACTGAACTGAATTAACGGAATAATTAATCTATCATATATTGATTTAAGCGAATTGGACCACATCAGGGTACATAGAGTTTGGGGGGCTAGCTAGTGATTAGATCCAAAGTTTTTTTCACCACATATCAATGTGGTCCATCCTAATTAAGTCCATTGTCTACCCCATTTTTGGTGGGGTCCCCAATCTATGAAAAA-TCGAATTCATTTTACTGCTCACTCACCAAAAATGTTCAACAGCCCCACGGTACATTATACAAGTAATAGTAATACAACAACATACATTAAACTTATATAG

>PI242028

AAGTTCAATTATCATCTTCAGATAAATCGGTAATTAACAAATTTAAATTTCAAATATGTTTTGAATCCTTGTATTTAG-----------GTTTGTCCTAACTCCTAGCCCTAACGAACTTCACTAAATTTTATGTGCCAAATAAATTTATAAAA-AAAAA--CATAAAGACATGATGCGG-TGGACTCACAAACTGAAGCATAC---------AAGTTATAAAGTTTTTGAAAGCGAAATAACGTCTTAAATTTTTTTAAATATTATAATCGAATATTTCTACGACTAACAACTGAACTGAATTAACGGAATAATTAATCTATCATATATTGATTTAAGCGAATTGGACCACATCAGGGTACATAGAGTTTGGGGGGCTAGCTAGTGATTAGATCCAAAGTTTTTTTCACCACATATCAATGTGGTCCATCCTAATTAAGTCCATTGTCTACCCCATTTTTGGTGGGGTCCCCAATCTATGAAAAA-TCGAATTCATTTTACTGCTCACTCACCAAAAATGTTCAACAGCCCCACGGTACATTATACAAGTAATAGTAATACAACAACATACATTAAACTTATATAG

>PI244093

AAGTTCAATTATCATCTTCAGATAAATCGGTAATTAACAAATTTAAATTTCAAATATGTTTTGAATCCTTGTATTTAG-----------GTTTGTCCTAACTCCTAGCCCTAACGAACTTCACTAAATTTTATGTGCCAAATAAATTTATAAAA-AAAAA--CATAAAGACATGATGCGG-TGGACTCACAAACTGAAGCATAC---------AAGTTATAAAGTTTTTGAAAGCGAAATAACGTCTTAAATTTTTTTAAATATTATAATCGAATATTTCTACGACTAACAACTGAACTGAATTAACGGAATAATTAATCTATCATATATTGATTTAAGCGAATTGGACCACATCAGGGTACATAGAGTTTGGGGGGCTAGCTAGTGATTAGATCCAAAGTTTTTTTCACCACATATCAATGTGGTCCATCCTAATTAAGTCCATTGTCTACCCCATTTTTGGTGGGGTCCCCAATCTATGAAAAA-TCGAATTCATTTTACTGCTCACTCACCAAAAATGTTCAACAGCCCCACGGTACATTATACAAGTAATAGTAATACAACAACATACATTAAACTTATATAG

>PI248181

AAGTTCAATTATCATCTTCAGATAAATCGGTAATTAACAAATTTAAATTTCAAATATGTTTTGAATCCTTGTATTTAG-----------GTTTGTCCTAACTCCTAGCCCTAACGAACTTCACTAAATTTTATGTGCCAAATAAATTTATAAAA-AAAAA--CATAAAGACATGATGCGG-TGGACTCACAAACTGAAGCATAC---------AAGTTATAAAGTTTTTGAAAGCGAAATAACGTCTTAAATTTTTTTAAATATTATAATCGAATATTTCTACGACTAACAACTGAACTGAATTAACGGAATAATTAATCTATCATATATTGATTTAAGCGAATTGGACCACATCAGGGTACATAGAGTTTGGGGGGCTAGCTAGTGATTAGATCCAAAGTTTTTTTCACCACATATCAATGTGGTCCATCCTAATTAAGTCCATTGTCTACCCCATTTTTGGTGGGGTCCCCAATCTATGAAAAA-TCGAATTCATTTTACTGCTCACTCACCAAAAATGTTCAACAGCCCCACGGTACATTATACAAGTAATAGTAATACAACAACATACATTAAACTTATATAG

>PI257244

AAGTTCAATTATCATCTTCAGATAAATCGGTAATTAACAAATTTAAATTTCAAATATGTTTTGAATCCTTGTATTTAG-----------GTTTGTCCTAACTCCTAGCCCTAACGAACTTCACTAAATTTTATGTGCCAAATAAATTTATAAAA-AAAAA--CATAAAGACATGATGCGG-TGGACTCACAAACTGAAGCATAC---------AAGTTATAAAGTTTTTGAAAGCGAAATAACGTCTTAAATTTTTTTAAATATTATAATCGAATATTTCTACGACTAACAACTGAACTGAATTAACGGAATAATTAATCTATCATATATTGATTTAAGCGAATTGGACCACATCAGGGTACATAGAGTTTGGGGGGCTAGCTAGTGATTAGATCCAAAGTTTTTTTCACCACATATCAATGTGGTCCATCCTAATTAAGTCCATTGTCTACCCCATTTTTGGTGGGGTCCCCAATCTATGAAAAA-TCGAATTCATTTTACTGCTCACTCACCAAAAATGTTCAACAGCCCCACGGTACATTATACAAGTAATAGTAATACAACAACATACATTAAACTTATATAG

>PI261624

AAGTTCAATTATCATCTTCAGATAAATCGGTAATTAACAAATTTAAATTTCAAATATGTTTTGAATCCTTGTATTTAG-----------GTTTGTCCTAACTCCTAGCCCTAACGAACTTCACTAAATTTTATGTGCCAAATAAATTTATAAAA-AAAAA--CATAAAGACATGATGCGG-TGGACTCACAAACTGAAGCATAC---------AAGTTATAAAGTTTTTGAAAGCGAAATAACGTCTTAAATTTTTTTAAATATTATAATCGAATATTTCTACGACTAACAACTGAACTGAATTAACGGAATAATTAATCTATCATATATTGATTTAAGCGAATTGGACCACATCAGGGTACATAGAGTTTGGGGGGCTAGCTAGTGATTAGATCCAAAGTTTTTTTCACCACATATCAATGTGGTCCATCCTAATTAAGTCCATTGTCTACCCCATTTTTGGTGGGGTCCCCAATCTATGAAAAA-TCGAATTCATTTTACTGCTCACTCACCAAAAATGTTCAACAGCCCCACGGTACATTATACAAGTAATAGTAATACAACAACATACATTAAACTTATATAG

>PI263027

AAGTTCAATTATCATCTTCAGATAAATCGGTAATTAACAAATTTAAATTTCAAATATGTTTTGAATCCTTGTATTTAG-----------GTTTGTCCTAACTCCTAGCCCTAACGAACTTCACTAAATTTTATGTGCCAAATAAATTTATAAAA-AAAAA--CATAAAGACATGATGCGG-TGGACTCACAAACTGAAGCATAC---------AAGTTATAAAGTTTTTGAAAGCGAAATAACGTCTTAAATTTTTTTAAATATTATAATCGAATATTTCTACGACTAACAACTGAACTGAATTAACGGAATAATTAATCTATCATATATTGATTTAAGCGAATTGGACCACATCAGGGTACATAGAGTTTGGGGGGCTAGCTAGTGATTAGATCCAAAGTTTTTTTCACCACATATCAATGTGGTCCATCCTAATTAAGTCCATTGTCTACCCCATTTTTGGTGGGGTCCCCAATCTATGAAAAA-TCGAATTCATTTTACTGCTCACTCACCAAAAATGTTCAACAGCCCCACGGTACATTATACAAGTAATAGTAATACAACAACATACATTAAACTTATATAG

>PI269798

AAGTTCAATTATCATCTTCAGATAAATCGGTAATTAACAAATTTAAATTTCAAATATGTTTTGAATCCTTGTATTTAG-----------GTTTGTCCTAACTCCTAGCCCTAACGAACTTCACTAAATTTTATGTGCCAAATAAATTTATAAAA-AAAAA--CATAAAGACATGATGCGG-TGGACTCACAAACTGAAGCATAC---------AAGTTATAAAGTTTTTGAAAGCGAAATAACGTCTTAAATTTTTTTAAATATTATAATCGAATATTTCTACGACTAACAACTGAACTGAATTAACGGAATAATTAATCTATCATATATTGATTTAAGCGAATTGGACCACATCAGGGTACATAGAGTTTGGGGGGCTAGCTAGTGATTAGATCCAAAGTTTTTTTCACCACATATCAATGTGGTCCATCCTAATTAAGTCCATTGTCTACCCCATTTTTGGTGGGGTCCCCAATCTATGAAAAA-TCGAATTCATTTTACTGCTCACTCACCAAAAATGTTCAACAGCCCCACGGTACATTATACAAGTAATAGTAATACAACAACATACATTAAACTTATATAG

>PI269821

AAGTTCAATTATCATCTTCAGATAAATCGGTAATTAACAAATTTAAATTTCAAATATGTTTTGAATCCTTGTATTTAG-----------GTTTGTCCTAACTCCTAGCCCTAACGAACTTCACTAAATTTTATGTGCCAAATAAATTTATAAAA-AAAAA--CATAAAGACATGATGCGG-TGGACTCACAAACTGAAGCATAC---------AAGTTATAAAGTTTTTGAAAGCGAAATAACGTCTTAAATTTTTTTAAATATTATAATCGAATATTTCTACGACTAACAACTGAACTGAATTAACGGAATAATTAATCTATCATATATTGATTTAAGCGAATTGGACCACATCAGGGTACATAGAGTTTGGGGGGCTAGCTAGTGATTAGATCCAAAGTTTTTTTCACCACATATCAATGTGGTCCATCCTAATTAAGTCCATTGTCTACCCCATTTTTGGTGGGGTCCCCAATCTATGAAAAA-TCGAATTCATTTTACTGCTCACTCACCAAAAATGTTCAACAGCCCCACGGTACATTATACAAGTAATAGTAATACAACAACATACATTAAACTTATATAG

>PI271035

AAGTTCAATTATCATCTTCAGATAAATCGGTAATTAACAAATTTAAATTTCAAATATGTTTTGAATCCTTGTATTTAG-----------GTTTGTCCTAACTCCTAGCCCTAACGAACTTCACTAAATTTTATGTGCCAAATAAATTTATAAAA-AAAAA--CATAAAGACATGATGCGG-TGGACTCACAAACTGAAGCATAC---------AAGTTATAAAGTTTTTGAAAGCGAAATAACGTCTTAAATTTTTTTAAATATTATAATCGAATATTTCTACGACTAACAACTGAACTGAATTAACGGAATAATTAATCTATCATATATTGATTTAAGCGAATTGGACCACATCAGGGTACATAGAGTTTGGGGGGCTAGCTAGTGATTAGATCCAAAGTTTTTTTCACCACATATCAATGTGGTCCATCCTAATTAAGTCCATTGTCTACCCCATTTTTGGTGGGGTCCCCAATCTATGAAAAA-TCGAATTCATTTTACTGCTCACTCACCAAAAATGTTCAACAGCCCCACGGTACATTATACAAGTAATAGTAATACAACAACATACATTAAACTTATATAG

>PI271511

AAGTTCAATTATCATCTTCAGATAAATCGGTAATTAACAAATTTAAATTTCAAATATGTTTTGAATCCTTGTATTTAG-----------GTTTGTCCTAACTCCTAGCCCTAACGAACTTCACTAAATTTTATGTGCCAAATAAATTTATAAAA-AAAAA--CATAAAGACATGATGCGG-TGGACTCACAAACTGAAGCATAC---------AAGTTATAAAGTTTTTGAAAGCGAAATAACGTCTTAAATTTTTTTAAATATTATAATCGAATATTTCTACGACTAACAACTGAACTGAATTAACGGAATAATTAATCTATCATATATTGATTTAAGCGAATTGGACCACATCAGGGTACATAGAGTTTGGGGGGCTAGCTAGTGATTAGATCCAAAGTTTTTTTCACCACATATCAATGTGGTCCATCCTAATTAAGTCCATTGTCTACCCCATTTTTGGTGGGGTCCCCAATCTATGAAAAA-TCGAATTCATTTTACTGCTCACTCACCAAAAATGTTCAACAGCCCCACGGTACATTATACAAGTAATAGTAATACAACAACATACATTAAACTTATATAG

>PI279825

AAGTTCAATTATCATCTTCAGATAAATCGGTAATTAACAAATTTAAATTTCAAATATGTTTTGAATCCTTGTATTTAG-----------GTTTGTCCTAACTCCTAGCCCTAACGAACTTCACTAAATTTTATGTGCCAAATAAATTTATAAAA-AAAAA--CATAAAGACATGATGCGG-TGGACTCACAAACTGAAGCATAC---------AAGTTATAAAGTTTTTGAAAGCGAAATAACGTCTTAAATTTTTTTAAATATTATAATCGAATATTTCTACGACTAACAACTGAACTGAATTAACGGAATAATTAATCTATCATATATTGATTTAAGCGAATTGGACCACATCAGGGTACATAGAGTTTGGGGGGCTAGCTAGTGATTAGATCCAAAGTTTTTTTCACCACATATCAATGTGGTCCATCCTAATTAAGTCCATTGTCTACCCCATTTTTGGTGGGGTCCCCAATCTATGAAAAA-TCGAATTCATTTTACTGCTCACTCACCAAAAATGTTCAACAGCCCCACGGTACATTATACAAGTAATAGTAATACAACAACATACATTAAACTTATATAG

>PI280603

AAGTTCAATTATCATCTTCAGATAAATCGGTAATTAACAAATTTAAATTTCAAATATGTTTTGAATCCTTGTATTTAG-----------GTTTGTCCTAACTCCTAGCCCTAACGAACTTCACTAAATTTTATGTGCCAAATAAATTTATAAAA-AAAAA--CATAAAGACATGATGCGG-TGGACTCACAAACTGAAGCATAC---------AAGTTATAAAGTTTTTGAAAGCGAAATAACGTCTTAAATTTTTTTAAATATTATAATCGAATATTTCTACGACTAACAACTGAACTGAATTAACGGAATAATTAATCTATCATATATTGATTTAAGCGAATTGGACCACATCAGGGTACATAGAGTTTGGGGGGCTAGCTAGTGATTAGATCCAAAGTTTTTTTCACCACATATCAATGTGGTCCATCCTAATTAAGTCCATTGTCTACCCCATTTTTGGTGGGGTCCCCAATCTATGAAAAA-TCGAATTCATTTTACTGCTCACTCACCAAAAATGTTCAACAGCCCCACGGTACATTATACAAGTAATAGTAATACAACAACATACATTAAACTTATATAG

>PI280611

AAGTTCAATTATCATCTTCAGATAAATCGGTAATTAACAAATTTAAATTTCAAATATGTTTTGAATCCTTGTATTTAG-----------GTTTGTCCTAACTCCTAGCCCTAACGAACTTCACTAAATTTTATGTGCCAAATAAATTTATAAAA-AAAAA--CATAAAGACATGATGCGG-TGGACTCACAAACTGAAGCATAC---------AAGTTATAAAGTTTTTGAAAGCGAAATAACGTCTTAAATTTTTTTAAATATTATAATCGAATATTTCTACGACTAACAACTGAACTGAATTAACGGAATAATTAATCTATCATATATTGATTTAAGCGAATTGGACCACATCAGGGTACATAGAGTTTGGGGGGCTAGCTAGTGATTAGATCCAAAGTTTTTTTCACCACATATCAATGTGGTCCATCCTAATTAAGTCCATTGTCTACCCCATTTTTGGTGGGGTCCCCAATCTATGAAAAA-TCGAATTCATTTTACTGCTCACTCACCAAAAATGTTCAACAGCCCCACGGTACATTATACAAGTAATAGTAATACAACAACATACATTAAACTTATATAG

>PI280616

AAGTTCAATTATCATCTTCAGATAAATCGGTAATTAACAAATTTAAATTTCAAATATGTTTTGAATCCTTGTATTTAG-----------GTTTGTCCTAACTCCTAGCCCTAACGAACTTCACTAAATTTTATGTGCCAAATAAATTTATAAAA-AAAAA--CATAAAGACATGATGCGG-TGGACTCACAAACTGAAGCATAC---------AAGTTATAAAGTTTTTGAAAGCGAAATAACGTCTTAAATTTTTTTAAATATTATAATCGAATATTTCTACGACTAACAACTGAACTGAATTAACGGAATAATTAATCTATCATATATTGATTTAAGCGAATTGGACCACATCAGGGTACATAGAGTTTGGGGGGCTAGCTAGTGATTAGATCCAAAGTTTTTTTCACCACATATCAATGTGGTCCATCCTAATTAAGTCCATTGTCTACCCCATTTTTGGTGGGGTCCCCAATCTATGAAAAA-TCGAATTCATTTTACTGCTCACTCACCAAAAATGTTCAACAGCCCCACGGTACATTATACAAGTAATAGTAATACAACAACATACATTAAACTTATATAG

>PI285715

AAGTTCAATTATCATCTTCAGATAAATCGGTAATTAACAAATTTAAATTTCAAATATGTTTTGAATCCTTGTATTTAG-----------GTTTGTCCTAACTCCTAGCCCTAACGAACTTCACTAAATTTTATGTGCCAAATAAATTTATAAAA-AAAAA--CATAAAGACATGATGCGG-TGGACTCACAAACTGAAGCATAC---------AAGTTATAAAGTTTTTGAAAGCGAAATAACGTCTTAAATTTTTTTAAATATTATAATCGAATATTTCTACGACTAACAACTGAACTGAATTAACGGAATAATTAATCTATCATATATTGATTTAAGCGAATTGGACCACATCAGGGTACATAGAGTTTGGGGGGCTAGCTAGTGATTAGATCCAAAGTTTTTTTCACCACATATCAATGTGGTCCATCCTAATTAAGTCCATTGTCTACCCCATTTTTGGTGGGGTCCCCAATCTATGAAAAA-TCGAATTCATTTTACTGCTCACTCACCAAAAATGTTCAACAGCCCCACGGTACATTATACAAGTAATAGTAATACAACAACATACATTAAACTTATATAG

>PI286431

AAGTTCAATTATCATCTTCAGATAAATCGGTAATTAACAAATTTAAATTTCAAATATGTTTTGAATCCTTGTATTTAG-----------GTTTGTCCTAACTCCTAGCCCTAACGAACTTCACTAAATTTTATGTGCCAAATAAATTTATAAAA-AAAAA--CATAAAGACATGATGCGG-TGGACTCACAAACTGAAGCATAC---------AAGTTATAAAGTTTTTGAAAGCGAAATAACGTCTTAAATTTTTTTAAATATTATAATCGAATATTTCTACGACTAACAACTGAACTGAATTAACGGAATAATTAATCTATCATATATTGATTTAAGCGAATTGGACCACATCAGGGTACATAGAGTTTGGGGGGCTAGCTAGTGATTAGATCCAAAGTTTTTTTCACCACATATCAATGTGGTCCATCCTAATTAAGTCCATTGTCTACCCCATTTTTGGTGGGGTCCCCAATCTATGAAAAA-TCGAATTCATTTTACTGCTCACTCACCAAAAATGTTCAACAGCCCCACGGTACATTATACAAGTAATAGTAATACAACAACATACATTAAACTTATATAG

>PI286607

AAGTTCAATTATCATCTTCAGATAAATCGGTAATTAACAAATTTAAATTTCAAATATGTTTTGAATCCTTGTATTTAG-----------GTTTGTCCTAACTCCTAGCCCTAACGAACTTCACTAAATTTTATGTGCCAAATAAATTTATAAAA-AAAAA--CATAAAGACATGATGCGG-TGGACTCACAAACTGAAGCATAC---------AAGTTATAAAGTTTTTGAAAGCGAAATAACGTCTTAAATTTTTTTAAATATTATAATCGAATATTTCTACGACTAACAACTGAACTGAATTAACGGAATAATTAATCTATCATATATTGATTTAAGCGAATTGGACCACATCAGGGTACATAGAGTTTGGGGGGCTAGCTAGTGATTAGATCCAAAGTTTTTTTCACCACATATCAATGTGGTCCATCCTAATTAAGTCCATTGTCTACCCCATTTTTGGTGGGGTCCCCAATCTATGAAAAA-TCGAATTCATTTTACTGCTCACTCACCAAAAATGTTCAACAGCCCCACGGTACATTATACAAGTAATAGTAATACAACAACATACATTAAACTTATATAG

>PI343987

AAGTTCAATTATCATCTTCAGATAAATCGGTAATTAACAAATTTAAATTTCAAATATGTTTTGAATCCTTGTATTTAG-----------GTTTGTCCTAACTCCTAGCCCTAACGAACTTCACTAAATTTTATGTGCCAAATAAATTTATAAAA-AAAAA--CATAAAGACATGATGCGG-TGGACTCACAAACTGAAGCATAC---------AAGTTATAAAGTTTTTGAAAGCGAAATAACGTCTTAAATTTTTTTAAATATTATAATCGAATATTTCTACGACTAACAACTGAACTGAATTAACGGAATAATTAATCTATCATATATTGATTTAAGCGAATTGGACCACATCAGGGTACATAGAGTTTGGGGGGCTAGCTAGTGATTAGATCCAAAGTTTTTTTCACCACATATCAATGTGGTCCATCCTAATTAAGTCCATTGTCTACCCCATTTTTGGTGGGGTCCCCAATCTATGAAAAA-TCGAATTCATTTTACTGCTCACTCACCAAAAATGTTCAACAGCCCCACGGTACATTATACAAGTAATAGTAATACAACAACATACATTAAACTTATATAG

>PI411143

AAGTTCAATTATCATCTTCAGATAAATCGGTAATTAACAAATTTAAATTTCAAATATGTTTTGAATCCTTGTATTTAG-----------GTTTGTCCTAACTCCTAGCCCTAACGAACTTCACTAAATTTTATGTGCCAAATAAATTTATAAAA-AAAAA--CATAAAGACATGATGCGG-TGGACTCACAAACTGAAGCATAC---------AAGTTATAAAGTTTTTGAAAGCGAAATAACGTCTTAAATTTTTTTAAATATTATAATCGAATATTTCTACGACTAACAACTGAACTGAATTAACGGAATAATTAATCTATCATATATTGATTTAAGCGAATTGGACCACATCAGGGTACATAGAGTTTGGGGGGCTAGCTAGTGATTAGATCCAAAGTTTTTTTCACCACATATCAATGTGGTCCATCCTAATTAAGTCCATTGTCTACCCCATTTTTGGTGGGGTCCCCAATCTATGAAAAA-TCGAATTCATTTTACTGCTCACTCACCAAAAATGTTCAACAGCCCCACGGTACATTATACAAGTAATAGTAATACAACAACATACATTAAACTTATATAG

>JI232psexo4F-TT8R8-F

ACCCATCAAGACAAATTGACTCATATACAACCGAGAGATGGGGTCCAATCGAAGAACCTCTCGATGATTCACTACAAGTTCAATTATCATCTTCAGATAAATCGGTAATTAACAAATTTAAATTTCAAATATGTTTTGAATCCTTGTATTTAGGTTTGTCCTAACTCCTAGCCCTAACGAACTTCACTAAATTTTATGTGCCAAATAAATTTATAAAAAAAAACATAAAGACATGATGCGGTGGACTCACAAACTGAAGCATACAAGTTATAAAGTTTTTGAAAGCGAAATAACGTCTTAAATTTTTTTAAATATTATAATCGAATATTTCTACGACTAACAACTGAACTGAATTAACGGAATAATTAATCTATCATATATTGATTTAAGCGAATTGGACCACATCAGGGTACATAGAGTTTGGGGGGCTAGCTAGTGATTAGATCCAAAGTTTTTTTCACCACATATCAATGTGGTCCATCCTAATTAAGTCCATTGTCTACCCCATTTTTGGTGGGGTCCCCAATCTATGAAAAATCGAATTCATTTTACTGCTCACTCACCAAAAATGTTCAACAGCCCCACGGTACATTATACAAGTAATAGTAATACAACAACATACATTAAACTTATATAGTAGTAATAAAACTTCTACTTTACTTCAATAATTATAAAAGTGTCGAGTGTAAATTTGGATTGTCTTCAACTAGGTACTATTTTGTGGG

>JI616-psexo4F-TT8R8-F

ACCCATCAAGACAAATTGACTCATATACAACCGAGAGATGGGGTCCAATCGAAGAACCTCTCGATGATTCACTACAAGTTCAATTATCATCTTCAGATAAATCGGTAATTAACAAATTTAAATTTCAAATATGTTTTGAATCCTTGTATTTAGGTTTGTCCTAACTCCTAGCCCTAACGAACTTCACTAAATTTTATGTGCCAAATAAATTTATAAAAAAAAACATAAAGACATGATGCGGTGGACTCACAAACTGAAGCATACAAGTTATAAAGTTTTTGAAAGCGAAATAACGTCTTAAATTTTTTTAAATATTATAATCGAATATTTCTACGACTAACAACTGAACTGAATTAACGGAATAATTAATCTATCATATATTGATTTAAGCGAATTGGACCACATCAGGGTACATAGAGTTTGGGGGGCTAGCTAGTGATTAGATCCAAAGTTTTTTTCACCACATATCAATGTGGTCCATCCTAATTAAGTCCATTGTCTACCCCATTTTTGGTGGGGTCCCCAATCTATGAAAAATCGAATTCATTTTACTGCTCACTCACCAAAAATGTTCAACAGCCCCACGGTACATTATACAAGTAATAGTAATACAACAACATACATTAAACTTATATAGTAGTAATAAAACTTCTACTTTACTTCAATAATTATAAAAGTGTCGAGTGTAAATTTGGATTGTCTTCAACTAGGTACTATTTTGTGGG

>JI871-psexo4F-TT8R8-F

ACCCATCAAGACAAATTGACTCATATACAACCGAGAGATGGGGTCCAATCGAAGAACCTCTCGATGATTCACTACAAGTTCAATTATCATCTTCAGATAAATCGGTAATTAACAAATTTAAATTTCAAATATGTTTTGAATCCTTGTATTTAGGTTTGTCCTAACTCCTAGCCCTAACGAACTTCACTAAATTTTATGTGCCAAATAAATTTATAAAAAAAAACATAAAGACATGATGCGGTGGACTCACAAACTGAAGCATACAAGTTATAAAGTTTTTGAAAGCGAAATAACGTCTTAAATTTTTTTAAATATTATAATCGAATATTTCTACGACTAACAACTGAACTGAATTAACGGAATAATTAATCTATCATATATTGATTTAAGCGAATTGGACCACATCAGGGTACATAGAGTTTGGGGGGCTAGCTAGTGATTAGATCCAAAGTTTTTTTCACCACATATCAATGTGGTCCATCCTAATTAAGTCCATTGTCTACCCCATTTTTGGTGGGGTCCCCAATCTATGAAAAATCGAATTCATTTTACTGCTCACTCACCAAAAATGTTCAACAGCCCCACGGTACATTATACAAGTAATAGTAATACAACAACATACATTAAACTTATATAGTAGTAATAAAA

>JI1497-psexo4F-TT8R8-F

ACCCATCAAGACAAATTGACTCATATACAACCGAGAGATGGGGTCCAATCGAAGAACCTCTCGATGATTCACTACAAGTTCAATTATCATCTTCAGATAAATCGGTAATTAACAAATTTAAATTTCAAATATGTTTTGAATCCTTGTATTTAGGTTTGTCCTAACTCCTAGCCCTAACGAACTTCACTAAATTTTATGTGCCAAATAAATTTATAAAAAAAAACATAAAGACATGATGCGGTGGACTCACAAACTGAAGCATACAAGTTATAAAGTTTTTGAAAGCGAAATAACGTCTTAAATTTTTTTAAATATTATAATCGAATATTTCTACGACTAACAACTGAACTGAATTAACGGAATAATTAATCTATCATATATTGATTTAAGCGAATTGGACCACATCAGGGTACATAGAGTTTGGGGGGCTAGCTAGTGATTAGATCCAAAGTTTTTTTCACCACATATCAATGTGGTCCATCCTAATTAAGTCCATTGTCTACCCCATTTTTGGTGGGGTCCCCAATCTATGAAAAATCGAATTCATTTTACTGCTCACTCACCAAAAATGTTCAACAGCCCCACGGTACATTATACAAGTAATAGTAATACAACAACATACATTAAACTTATATAGTAGTAATAAAACTTCTACTTTACTTCAATAATTATAAAAGTGTCGAGTGTAAATTTGGATTGTCTTCAACTAGGTA

>JI1512-psexo4F-TT8R8-F

ACCCATCAAGACAAATTGACTCATATACAACCGAGAGATGGGGTCCAATCGAAGAACCTCTCGATGATTCACTACAAGTTCAATTATCATCTTCAGATAAATCGGTAATTAACAAATTTAAATTTCAAATATGTTTTGAATCCTTGTATTTAGGTTTGTCCTAACTCCTAGCCCTAACGAACTTCACTAAATTTTATGTGCCAAATAAATTTATAAAAAAAAACATAAAGACATGATGCGGTGGACTCACAAACTGAAGCATACAAGTTATAAAGTTTTTGAAAGCGAAATAACGTCTTAAATTTTTTTAAATATTATAATCGAATATTTCTACGACTAACAACTGAACTGAATTAACGGAATAATTAATCTATCATATATTGATTTAAGCGAATTGGACCACATCAGGGTACATAGAGTTTGGGGGGCTAGCTAGTGATTAGATCCAAAGTTTTTTTCACCACATATCAATGTGGTCCATCCTAATTAAGTCCATTGTCTACCCCATTTTTGGTGGGGTCCCCAATCTATGAAAAATCGAATTCATTTTACTGCTCACTCACCAAAAATGTTCAACAGCCCCACGGTACATTATACAAGTAATAGTAATACAACAACATACATTAAACTTATATAGTAGTAATAAAACTTCTACTTTACTTCAATAATTATAAAAGTGTCGAGTGTAAATTTGGATTGTCTTCAACTAGGTACTATTTTGTGG

>JI1792-psexo4F-TT8R8-F

ACCCATCAAGACAAATTGACTCATATACAACCGAGAGATGGGGTCCAATCGAAGAACCTCTCGATGATTCACTACAAGTTCAATTATCATCTTCAGATAAATCGGTAATTAACAAATTTAAATTTCAAATATGTTTTGAATCCTTGTATTTAGGTTTGTCCTAACTCCTAGCCCTAACGAACTTCACTAAATTTTATGTGCCAAATAAATTTATAAAAAAAAACATAAAGACATGATGCGGTGGACTCACAAACTGAAGCATACAAGTTATAAAGTTTTTGAAAGCGAAATAACGTCTTAAATTTTTTTAAATATTATAATCGAATATTTCTACGACTAACAACTGAACTGAATTAACGGAATAATTAATCTATCATATATTGATTTAAGCGAATTGGACCACATCAGGGTACATAGAGTTTGGGGGGCTAGCTAGTGATTAGATCCAAAGTTTTTTTCACCACATATCAATGTGGTCCATCCTAATTAAGTCCATTGTCTACCCCATTTTTGGTGGGGTCCCCAATCTATGAAAAATCGAATTCATTTTACTGCTCACTCACCAAAAATGTTCAACAGCCCCACGGTACATTATACAAGTAATAGTAATACAACAACATACATTAAACTTATATAGTAGTAATAAAACTTCTACTTTACTTCAATAATTATAAAAGT

>JI2647-psexo4F-TT8R8-F

ACCCATCAAGACAAATTGACTCATATACAACCGAGAGATGGGGTCCAATCGAAGAACCTCTCGATGATTCACTACAAGTTCAATTATCATCTTCAGATAAATCGGTAATTAACAAATTTAAATTTCAAATATGTTTTGAATCCTTGTATTTAGGTTTGTCCTAACTCCTAGCCCTAACGAACTTCACTAAATTTTATGTGCCAAATAAATTTATAAAAAAAAACATAAAGACATGATGCGGTGGACTCNCAAACTGAAGCATACAAGTTATAAAGTTTTTGAAAGCGAAATAACGTCTTAAATTTTTTTAAATATTATAATCGAATATTTCTACGACTAACAACTGAACTGAATTAACGGAATAATTAATCTATCATATATTGATTTAAGCGAATTGGACCACATCAGGGTACATAGAGTTTGGGGGGCTAGCTAGTGATTAGATCCAAAGTTTTTTTCACCACATATCAATGTGGTCCATCCTAATTAAGTCCATTGTCTACCCCATTTTTGGTGGGGTCCCCAATCTATGAAAAATCGAATTCATTTTACTGCTCACTCACCAAAAATGTTCAACAGCCCCACGGnACATTATACAAGTAATAGTAATACAACAACATACATTAAACTTATATAGTAGTAATAAAACTTCTACTTTACTTCAATAATTATAAAAGTGTCGAGTGTAAATTTGGATTGTCTTCAACTAGGTACTA

>JI3003-psexo4F-TT8R8-F

ACCCATCAAGACAAATTGACTCATATACAACCGAGAGATGGGGTCCAATCGAAGAACCTCTCGATGATTCACTACAAGTTCAATTATCATCTTCAGATAAATCGGTAATTAACAAATTTAAATTTCAAATATGTTTTGAATCCTTGTATTTAGGTTTGTCCTAACTCCTAGCCCTAACGAACTTCACTAAATTTTATGTGCCAAATAAATTTATAAAAAAAAACATAAAGACATGATGCGGTGGACTCACAAACTGAAGCATACAAGTTATAAAGTTTTTGAAAGCGAAATAACGTCTTAAATTTTTTTAAATATTATAATCGAATATTTCTACGACTAACAACTGAACTGAATTAACGGAATAATTAATCTATCATATATTGATTTAAGCGAATTGGACCACATCAGGGTACATAGAGTTTGGGGGGCTAGCTAGTGATTAGATCCAAAGTTTTTTTCACCACATATCAATGTGGTCCATCCTAATTAAGTCCATTGTCTACCCCATTTTTGGTGGGGTCCCCAATCTATGAAAAATCGAATTCATTTTACTGCTCACTCACCAAAAATGTTCAACAGCCCCACGGTACATTATACAAGTAATAGTAATACAACAACATACATTAAACTTATATAGTAGTAATAAAACTTCTACTTTACTTCAATAATTATAAAAGTGTCGAGTGTAAATTTGGATTGTCTTCAACTAGGTACTATTTTGTGGG

>JI4-exo4F-TT8R8-F

GTTAGTAATCAAGGAAACCCATCAAGACAAATTGACTCATATACAACCGAGAGATGGGGTCCAATCGAAGAACCTCTCGATGATTCACTACAAGTTCAATTATCATCTTCAGATAAATCGGTAATTAACAAATTTAAATTTCAAATATGTTTTGAATCCTTGTATTTAGGTTTGTCCTAACTCCTAGCCCTAACGAACTTCACTAAATTTTATGTGCCAAATAAATTTATAAAAAAAAACATAAAGACATGATGCGGTGGACTCACAAACTGAAGCATACAAGTTATAAAGTTTTTGAAAGCGAAATAACGTCTTAAATTTTTTTAAATATTATAATCGAATATTTCTACGACTAACAACTGAACTGAATTAACGGAATAATTAATCTATCATATATTGATTTAAGCGAATTGGACCACATCAGGGTACATAGAGTTTGGGGGGCTAGCTAGTGATTAGATCCAAAGTTTTTTTCACCACATATC

>Caméor-Ps-A-Exo4F-inT5R-F

CGGGTCACCCAACGATGGGTCGAATAATTTGGACTCGGATTTTCATTTGTTGGCCGTTAGTAATCAAGGAAACCCATCAAGACAAATTGACTCATATACAACCGAGAGATGGGGTCCAATCGAAGAACCTCTCGATGATTCACTACAAGTTCAATTATCATCTTCAGATAAATCGGTAATTAACAAATTTAAATTTCAAATATGTTTTGAATCCTTGTATTTAGGTTTGTCCTAACTCCTAGCCCTAACGAACTTCACTAAATTTTATGTGCCAAATAAATTTATAAAAAAAAACATAAAGACATGATGCGGTGGACTCAC

>JI504A-PsTT8F2R7-F

CCAACCAAGGTCATAACCAACACGCAACCTCTATAATAGAGGCTGCGGAACCGAGTGAACTCATGCAAATTGAAATGCCCGATGATATTCGGATCGGGTCACCCAACGATGGGTCGAATAATTTGGACTCGGATTTTCATTTGTTGGCCGTTAGTAATCAAGGAAACCCATCAAGACAAATTGACTCATATACAACCGAGAGATGGGGTCCAATCGAAGAACCTCTCGATGATTCACTACAAGTTCAATTATCATCTTCAGATAAATCGGTAATTAACAAATTTAAATTTCAAATATGTTTTGAATCCTTGTATTTAGGTTTGTCCTAACTCCTAGCCCTAACGAACTTCACTAAATTTTATGTGCCAAATAAATTTATAAAAAAAAACATAAAGACATGATGCGGTGGACTCACAAACTGAAGCATACAAGTTATAAAGTTTTTGAAAGCGAAATAACGTCTTAAATTTTTTTAAATATTATAATCGAATATTTCTACGACTAACAACTGAACTGAATTAACGGAATAATTAATCTATCATATATTGATTTAAGCGAATTGGACCACATCAGGGTACATAGAGTTTGGGGGGCTAGCTAGTGATTAGATCCAAAGTTTTTTTCACCACATATC

>JI1189A-PsTT8F2R7-F

CCAACCAAGGTCATAACCAACACGCAACCTCTATAATAGAGGCTGCGGAACCGAGTGAACTCATGCAAATTGAAATGCCCGATGATATTCGGATCGGGTCACCCAACGATGGGTCGAATAATTTGGACTCGGATTTTCATTTGTTGGCCGTTAGTAATCAAGGAAACCCATCAAGACAAATTGACTCATATACAACCGAGAGATGGGGTCCAATCGAAGAACCTCTCGATGATTCACTACAAGTTCAATTATCATCTTCAGATAAATCGGTAATTAACAAATTTAAATTTCAAATATGTTTTGAATCCTTGTATTTAGGTTTGTCCTAACTCCTAGCCCTAACGAACTTCACTAAATTTTATGTGCCAAATAAATTTATAAAAAAAAACATAAAGACATGATGCGGTGGACTCACAAACTGAAGCATACAAGTTATAAAGTTTTTGAAAGCGAAATAACGTCTTAAATTTTTTTAAATATTATAATCGAATATTTCTACGACTAACAACTGAACTGAATTAACGGAATAATTAATCTATCATATATTGATTTAAGCGAATTGGACCACATCAGGGTACATAGAGTTTGGGGGGCTAGCTAGTGATTAGATCCAAAGTTTTTTTCACCACATATC

>JI1229A-PsTT8F2R7-F

CCAACCAAGGTCATAACCAACACGCAACCTCTATAATAGAGGCTGCGGAACCGAGTGAACTCATGCAAATTGAAATGCCCGATGATATTCGGATCGGGTCACCCAACGATGGGTCGAATAATTTGGACTCGGATTTTCATTTGTTGGCCGTTAGTAATCAAGGAAACCCATCAAGACAAATTGACTCATATACAACCGAGAGATGGGGTCCAATCGAAGAACCTCTCGATGATTCACTACAAGTTCAATTATCATCTTCAGATAAATCGGTAATTAACAAATTTAAATTTCAAATATGTTTTGAATCCTTGTATTTAGGTTTGTCCTAACTCCTAGCCCTAACGAACTTCACTAAATTTTATGTGCCAAATAAATTTATAAAAAAAAACATAAAGACATGATGCGGTGGACTCACAAACTGAAGCATACAAGTTATAAAGTTTTTGAAAGCGAAATAACGTCTTAAATTTTTTTAAATATTATAATCGAATATTTCTACGACTAACAACTGAACTGAATTAACGGAATAATTAATCTATCATATATTGATTTAAGCGAATTGGACCACATCAGGGTACATAGAGTTTGGGGGGCTAGCTAGTGATTAGATCCAAAGTTTTTTTCACCACATATC

>JI2737A-PsTT8F2R7-F

CCAACCAAGGTCATAACCAACACGCAACCTCTATAATAGAGGCTGCGGAACCGAGTGAACTCATGCAAATTGAAATGCCCGATGATATTCGGATCGGGTCACCCAACGATGGGTCGAATAATTTGGACTCGGATTTTCATTTGTTGGCCGTTAGTAATCAAGGAAACCCATCAAGACAAATTGACTCATATACAACCGAGAGATGGGGTCCAATCGAAGAACCTCTCGATGATTCACTACAAGTTCAATTATCATCTTCAGATAAATCGGTAATTAACAAATTTAAATTTCAAATATGTTTTGAATCCTTGTATTTAGGTTTGTCCTAACTCCTAGCCCTAACGAACTTCACTAAATTTTATGTGCCAAATAAATTTATAAAAAAAAACATAAAGACATGATGCGGTGGACTCACAAACTGAAGCATACAAGTTATAAAGTTTTTGAAAGCGAAATAACGTCTTAAATTTTTTTAAATATTATAATCGAATATTTCTACGACTAACAACTGAACTGAATTAACGGAATAATTAATCTATCATATATTGATTTAAGCGAATTGGACCACATCAGGGTACATAGAGTTTGGGGGGCTAGCTAGTGATTAGATCCAAAGTTTTTTTCACCACATATC

>JI2462-PsTT8F2R7-inT5R

AGGATGACGAAGATGACGAAGTTGAA-TCTGGATCTGAAGATGAAACCAACCAAGGTCATAACCAACACGCAACCTCTATAATAGAGGCTGCGGAACCGAGTGAACTCATGCAAATTGAAATGCCCGATGATATTCGGATCGGGTCACCCAACGATGGGTCGAATAATTTGGACTCGGATTTTCATTTGTTGGCCGTTAGTAATCAAGGAAACCCATCAAGACAAATTGACTCATATACAACCGAGAGATGGGGTCCAATCGAAGAACCTCTCGATGATTCACTACAAGTTCAATTATCATCTTCAGATAAATCGGTAATTAACAAATTTAAATTTCAAATATGTTTTGAATCCTTGTATTTAGGTTTGTCCTAACTCCTAGCCCTAACGAACTTCACTAAATTTTATGTGCCAAATAAA

>JI2479-PsTT8F2R7-inT5R

AAGAGGATGACGAAGATGACGAAGTTGAA-TCTGGATCTGAAGATGAAACCAACCAAGGTCATAACCAACACGCAACCTCTATAATAGAGGCTGCGGAACCGAGTGAACTCATGCAAATTGAAATGCCCGATGATATTCGGATCGGGTCACCCAACGATGGGTCGAATAATTTGGACTCGGATTTTCATTTGTTGGCCGTTAGTAATCAAGGAAACCCATCAAGACAAATTGACTCATATACAACCGAGAGATGGGGTCCAATCGAAGAACCTCTCGATGATTCACTACAAGTTCAATTATCATCTTCAGATAAATCGGTAATTAACAAATTTAAATTTCAAATATGTTTTGAATCCTTGTATTTAGGTTTGTCCTAACTCCTAGCCCTAACGAACTTCACTAAATTTTATGTGCCAAATAAA

>Torsdag-PsTT8F2-R7

CCAACCAAGGTCATAACCAACACGCAACCTCTATAATAGAGGCTGCGGAACCGAGTGAACTCATGCAAATTGAAATGCCCGATGATATTCGGATCGGGTCACCCAACGATGGGTCGAATAATTTGGACTCGGATTTTCATTTGTTGGCCGTTAGTAATCAAGGAAACCCATCAAGACAAATTGACTCATATACAACCGAGAGATGGGGTCCAATCGAAGAACCTCTCGATGATTCACTACAAGTTCAATTATCATCTTCAGATAAATCGGTAATTAACAAATTTAAATTTCAAATATGTTTTGAATCCTTGTATTTAGGTTTGTCCTAACTCCTAGCCCTAACGAACTTCACTAAATTTTATGTGCCAAATAAATTTATAAAAAAAAACATAAAGACATGATGCGGTGGACTCACAAACTGAAGCATACAAGTTATAAAGTTTTTGAAAGCGAAATAACGTCTTAAATTTTTTTAAATATTATAATCGAATATTTCTACGACTAACAACTGAACTGAATTAWCGGAATAATTAATCTATCATATATTGATTTAAGCGAATTGGACCACATCAGGGTACATAGAGTTTGGGGGGCTAGCTAGTGATTAGATCCAAAGTTT

>PI169608

AAGTTCAATTATCATCTTCAGGTAAATCGGTAATTAACAAATTTAAATTTCAAATATGTTTTGAATCCTTGTATTTAG-----------GTTTGTCCTAACTCCTAGCCCTAACGAACTTCACTAAATTTTATGTGCCAAATAAATTTATAAAA-AAAAA--CATAAAGACATGATGCGG-TGGACTCACAAACTGAAGCATAC---------AAGTTATAAAGTTTTTGAAAGCGAAATAACGTCTTAAATTTTTTTAAATATTATAATCGAATATTTCTACGACTAACAACTGAACTGAATTAACGGAATAATTAATCTATCATATATTGATTTAAGCGAATTGGACCACATCAGGGTACATAGAGTTTGGGGGGCTAGCTAGTGATTAGATCCAAAGTTTTTCTCACCACATATCAATGTGGTCCATCCTAATTAAGTCCATTGTCTACCCCATTTTTGGTGGGGTCCCCAATCTATGAAAAA-TCGAATTCATTTTACTGCTCACTCACCAAAAATGTTCAACAGCCCCACGGTACATTATACAAGTAATAGTAATACAACAACATACATTAAACTTATATAG

>PI180693

AAGTTCAATTATCATCTTCAGGTAAATCGGTAATTAACAAATTTAAATTTCAAATATGTTTTGAATCCTTGTATTTAG-----------GTTTGTCCTAACTCCTAGCCCTAACGAACTTCACTAAATTTTATGTGCCAAATAAATTTATAAAA-AAAAA--CATAAAGACATGATGCGG-TGGACTCACAAACTGAAGCATAC---------AAGTTATAAAGTTTTTGAAAGCGAAATAACGTCTTAAATTTTTTTAAATATTATAATCGAATATTTCTACGACTAACAACTGAACTGAATTAACGGAATAATTAATCTATCATATATTGATTTAAGCGAATTGGACCACATCAGGGTACATAGAGTTTGGGGGGCTAGCTAGTGATTAGATCCAAAGTTTTTCTCACCACATATCAATGTGGTCCATCCTAATTAAGTCCATTGTCTACCCCATTTTTGGTGGGGTCCCCAATCTATGAAAAA-TCGAATTCATTTTACTGCTCACTCACCAAAAATGTTCAACAGCCCCACGGTACATTATACAAGTAATAGTAATACAACAACATACATTAAACTTATATAG

>PI184128

AAGTTCAATTATCATCTTCAGGTAAATCGGTAATTAACAAATTTAAATTTCAAATATGTTTTGAATCCTTGTATTTAG-----------GTTTGTCCTAACTCCTAGCCCTAACGAACTTCACTAAATTTTATGTGCCAAATAAATTTATAAAA-AAAAA--CATAAAGACATGATGCGG-TGGACTCACAAACTGAAGCATAC---------AAGTTATAAAGTTTTTGAAAGCGAAATAACGTCTTAAATTTTTTTAAATATTATAATCGAATATTTCTACGACTAACAACTGAACTGAATTAACGGAATAATTAATCTATCATATATTGATTTAAGCGAATTGGACCACATCAGGGTACATAGAGTTTGGGGGGCTAGCTAGTGATTAGATCCAAAGTTTTTCTCACCACATATCAATGTGGTCCATCCTAATTAAGTCCATTGTCTACCCCATTTTTGGTGGGGTCCCCAATCTATGAAAAA-TCGAATTCATTTTACTGCTCACTCACCAAAAATGTTCAACAGCCCCACGGTACATTATACAAGTAATAGTAATACAACAACATACATTAAACTTATATAG

>PI193590

AAGTTCAATTATCATCTTCAGGTAAATCGGTAATTAACAAATTTAAATTTCAAATATGTTTTGAATCCTTGTATTTAG-----------GTTTGTCCTAACTCCTAGCCCTAACGAACTTCACTAAATTTTATGTGCCAAATAAATTTATAAAA-AAAAA--CATAAAGACATGATGCGG-TGGACTCACAAACTGAAGCATAC---------AAGTTATAAAGTTTTTGAAAGCGAAATAACGTCTTAAATTTTTTTAAATATTATAATCGAATATTTCTACGACTAACAACTGAACTGAATTAACGGAATAATTAATCTATCATATATTGATTTAAGCGAATTGGACCACATCAGGGTACATAGAGTTTGGGGGGCTAGCTAGTGATTAGATCCAAAGTTTTTCTCACCACATATCAATGTGGTCCATCCTAATTAAGTCCATTGTCTACCCCATTTTTGGTGGGGTCCCCAATCTATGAAAAA-TCGAATTCATTTTACTGCTCACTCACCAAAAATGTTCAACAGCCCCACGGTACATTATACAAGTAATAGTAATACAACAACATACATTAAACTTATATAG

>PI195020

AAGTTCAATTATCATCTTCAGGTAAATCGGTAATTAACAAATTTAAATTTCAAATATGTTTTGAATCCTTGTATTTAG-----------GTTTGTCCTAACTCCTAGCCCTAACGAACTTCACTAAATTTTATGTGCCAAATAAATTTATAAAA-AAAAA--CATAAAGACATGATGCGG-TGGACTCACAAACTGAAGCATAC---------AAGTTATAAAGTTTTTGAAAGCGAAATAACGTCTTAAATTTTTTTAAATATTATAATCGAATATTTCTACGACTAACAACTGAACTGAATTAACGGAATAATTAATCTATCATATATTGATTTAAGCGAATTGGACCACATCAGGGTACATAGAGTTTGGGGGGCTAGCTAGTGATTAGATCCAAAGTTTTTCTCACCACATATCAATGTGGTCCATCCTAATTAAGTCCATTGTCTACCCCATTTTTGGTGGGGTCCCCAATCTATGAAAAA-TCGAATTCATTTTACTGCTCACTCACCAAAAATGTTCAACAGCCCCACGGTACATTATACAAGTAATAGTAATACAACAACATACATTAAACTTATATAG

>PI195631

AAGTTCAATTATCATCTTCAGGTAAATCGGTAATTAACAAATTTAAATTTCAAATATGTTTTGAATCCTTGTATTTAG-----------GTTTGTCCTAACTCCTAGCCCTAACGAACTTCACTAAATTTTATGTGCCAAATAAATTTATAAAA-AAAAA--CATAAAGACATGATGCGG-TGGACTCACAAACTGAAGCATAC---------AAGTTATAAAGTTTTTGAAAGCGAAATAACGTCTTAAATTTTTTTAAATATTATAATCGAATATTTCTACGACTAACAACTGAACTGAATTAACGGAATAATTAATCTATCATATATTGATTTAAGCGAATTGGACCACATCAGGGTACATAGAGTTTGGGGGGCTAGCTAGTGATTAGATCCAAAGTTTTTCTCACCACATATCAATGTGGTCCATCCTAATTAAGTCCATTGTCTACCCCATTTTTGGTGGGGTCCCCAATCTATGAAAAA-TCGAATTCATTTTACTGCTCACTCACCAAAAATGTTCAACAGCCCCACGGTACATTATACAAGTAATAGTAATACAACAACATACATTAAACTTATATAG

>PI257592

AAGTTCAATTATCATCTTCAGGTAAATCGGTAATTAACAAATTTAAATTTCAAATATGTTTTGAATCCTTGTATTTAG-----------GTTTGTCCTAACTCCTAGCCCTAACGAACTTCACTAAATTTTATGTGCCAAATAAATTTATAAAA-AAAAA--CATAAAGACATGATGCGG-TGGACTCACAAACTGAAGCATAC---------AAGTTATAAAGTTTTTGAAAGCGAAATAACGTCTTAAATTTTTTTAAATATTATAATCGAATATTTCTACGACTAACAACTGAACTGAATTAACGGAATAATTAATCTATCATATATTGATTTAAGCGAATTGGACCACATCAGGGTACATAGAGTTTGGGGGGCTAGCTAGTGATTAGATCCAAAGTTTTTCTCACCACATATCAATGTGGTCCATCCTAATTAAGTCCATTGTCTACCCCATTTTTGGTGGGGTCCCCAATCTATGAAAAA-TCGAATTCATTTTACTGCTCACTCACCAAAAATGTTCAACAGCCCCACGGTACATTATACAAGTAATAGTAATACAACAACATACATTAAACTTATATAG

>PI269782

AAGTTCAATTATCATCTTCAGGTAAATCGGTAATTAACAAATTTAAATTTCAAATATGTTTTGAATCCTTGTATTTAG-----------GTTTGTCCTAACTCCTAGCCCTAACGAACTTCACTAAATTTTATGTGCCAAATAAATTTATAAAA-AAAAA--CATAAAGACATGATGCGG-TGGACTCACAAACTGAAGCATAC---------AAGTTATAAAGTTTTTGAAAGCGAAATAACGTCTTAAATTTTTTTAAATATTATAATCGAATATTTCTACGACTAACAACTGAACTGAATTAACGGAATAATTAATCTATCATATATTGATTTAAGCGAATTGGACCACATCAGGGTACATAGAGTTTGGGGGGCTAGCTAGTGATTAGATCCAAAGTTTTTCTCACCACATATCAATGTGGTCCATCCTAATTAAGTCCATTGTCTACCCCATTTTTGGTGGGGTCCCCAATCTATGAAAAA-TCGAATTCATTTTACTGCTCACTCACCAAAAATGTTCAACAGCCCCACGGTACATTATACAAGTAATAGTAATACAACAACATACATTAAACTTATATAG

>PI272204

AAGTTCAATTATCATCTTCAGGTAAATCGGTAATTAACAAATTTAAATTTCAAATATGTTTTGAATCCTTGTATTTAG-----------GTTTGTCCTAACTCCTAGCCCTAACGAACTTCACTAAATTTTATGTGCCAAATAAATTTATAAAA-AAAAA--CATAAAGACATGATGCGG-TGGACTCACAAACTGAAGCATAC---------AAGTTATAAAGTTTTTGAAAGCGAAATAACGTCTTAAATTTTTTTAAATATTATAATCGAATATTTCTACGACTAACAACTGAACTGAATTAACGGAATAATTAATCTATCATATATTGATTTAAGCGAATTGGACCACATCAGGGTACATAGAGTTTGGGGGGCTAGCTAGTGATTAGATCCAAAGTTTTTCTCACCACATATCAATGTGGTCCATCCTAATTAAGTCCATTGTCTACCCCATTTTTGGTGGGGTCCCCAATCTATGAAAAA-TCGAATTCATTTTACTGCTCACTCACCAAAAATGTTCAACAGCCCCACGGTACATTATACAAGTAATAGTAATACAACAACATACATTAAACTTATATAG

>PI272218

AAGTTCAATTATCATCTTCAGGTAAATCGGTAATTAACAAATTTAAATTTCAAATATGTTTTGAATCCTTGTATTTAG-----------GTTTGTCCTAACTCCTAGCCCTAACGAACTTCACTAAATTTTATGTGCCAAATAAATTTATAAAA-AAAAA--CATAAAGACATGATGCGG-TGGACTCACAAACTGAAGCATAC---------AAGTTATAAAGTTTTTGAAAGCGAAATAACGTCTTAAATTTTTTTAAATATTATAATCGAATATTTCTACGACTAACAACTGAACTGAATTAACGGAATAATTAATCTATCATATATTGATTTAAGCGAATTGGACCACATCAGGGTACATAGAGTTTGGGGGGCTAGCTAGTGATTAGATCCAAAGTTTTTCTCACCACATATCAATGTGGTCCATCCTAATTAAGTCCATTGTCTACCCCATTTTTGGTGGGGTCCCCAATCTATGAAAAA-TCGAATTCATTTTACTGCTCACTCACCAAAAATGTTCAACAGCCCCACGGTACATTATACAAGTAATAGTAATACAACAACATACATTAAACTTATATAG

>PI285719

AAGTTCAATTATCATCTTCAGGTAAATCGGTAATTAACAAATTTAAATTTCAAATATGTTTTGAATCCTTGTATTTAG-----------GTTTGTCCTAACTCCTAGCCCTAACGAACTTCACTAAATTTTATGTGCCAAATAAATTTATAAAA-AAAAA--CATAAAGACATGATGCGG-TGGACTCACAAACTGAAGCATAC---------AAGTTATAAAGTTTTTGAAAGCGAAATAACGTCTTAAATTTTTTTAAATATTATAATCGAATATTTCTACGACTAACAACTGAACTGAATTAACGGAATAATTAATCTATCATATATTGATTTAAGCGAATTGGACCACATCAGGGTACATAGAGTTTGGGGGGCTAGCTAGTGATTAGATCCAAAGTTTTTCTCACCACATATCAATGTGGTCCATCCTAATTAAGTCCATTGTCTACCCCATTTTTGGTGGGGTCCCCAATCTATGAAAAA-TCGAATTCATTTTACTGCTCACTCACCAAAAATGTTCAACAGCCCCACGGTACATTATACAAGTAATAGTAATACAACAACATACATTAAACTTATATAG

>PI331414

AAGTTCAATTATCATCTTCAGGTAAATCGGTAATTAACAAATTTAAATTTCAAATATGTTTTGAATCCTTGTATTTAG-----------GTTTGTCCTAACTCCTAGCCCTAACGAACTTCACTAAATTTTATGTGCCAAATAAATTTATAAAA-AAAAA--CATAAAGACATGATGCGG-TGGACTCACAAACTGAAGCATAC---------AAGTTATAAAGTTTTTGAAAGCGAAATAACGTCTTAAATTTTTTTAAATATTATAATCGAATATTTCTACGACTAACAACTGAACTGAATTAACGGAATAATTAATCTATCATATATTGATTTAAGCGAATTGGACCACATCAGGGTACATAGAGTTTGGGGGGCTAGCTAGTGATTAGATCCAAAGTTTTTCTCACCACATATCAATGTGGTCCATCCTAATTAAGTCCATTGTCTACCCCATTTTTGGTGGGGTCCCCAATCTATGAAAAA-TCGAATTCATTTTACTGCTCACTCACCAAAAATGTTCAACAGCCCCACGGTACATTATACAAGTAATAGTAATACAACAACATACATTAAACTTATATAG

>PI340128

AAGTTCAATTATCATCTTCAGGTAAATCGGTAATTAACAAATTTAAATTTCAAATATGTTTTGAATCCTTGTATTTAG-----------GTTTGTCCTAACTCCTAGCCCTAACGAACTTCACTAAATTTTATGTGCCAAATAAATTTATAAAA-AAAAA--CATAAAGACATGATGCGG-TGGACTCACAAACTGAAGCATAC---------AAGTTATAAAGTTTTTGAAAGCGAAATAACGTCTTAAATTTTTTTAAATATTATAATCGAATATTTCTACGACTAACAACTGAACTGAATTAACGGAATAATTAATCTATCATATATTGATTTAAGCGAATTGGACCACATCAGGGTACATAGAGTTTGGGGGGCTAGCTAGTGATTAGATCCAAAGTTTTTCTCACCACATATCAATGTGGTCCATCCTAATTAAGTCCATTGTCTACCCCATTTTTGGTGGGGTCCCCAATCTATGAAAAA-TCGAATTCATTTTACTGCTCACTCACCAAAAATGTTCAACAGCCCCACGGTACATTATACAAGTAATAGTAATACAACAACATACATTAAACTTATATAG

>PI343331

AAGTTCAATTATCATCTTCAGGTAAATCGGTAATTAACAAATTTAAATTTCAAATATGTTTTGAATCCTTGTATTTAG-----------GTTTGTCCTAACTCCTAGCCCTAACGAACTTCACTAAATTTTATGTGCCAAATAAATTTATAAAA-AAAAA--CATAAAGACATGATGCGG-TGGACTCACAAACTGAAGCATAC---------AAGTTATAAAGTTTTTGAAAGCGAAATAACGTCTTAAATTTTTTTAAATATTATAATCGAATATTTCTACGACTAACAACTGAACTGAATTAACGGAATAATTAATCTATCATATATTGATTTAAGCGAATTGGACCACATCAGGGTACATAGAGTTTGGGGGGCTAGCTAGTGATTAGATCCAAAGTTTTTCTCACCACATATCAATGTGGTCCATCCTAATTAAGTCCATTGTCTACCCCATTTTTGGTGGGGTCCCCAATCTATGAAAAA-TCGAATTCATTTTACTGCTCACTCACCAAAAATGTTCAACAGCCCCACGGTACATTATACAAGTAATAGTAATACAACAACATACATTAAACTTATATAG

>PI270536

AAGTTCAATTATCATCTTCAGGTAAATCGGTAATTAACAAATTTAAATTTCAAATATGTTTTGAATCCTTGTATTTAG-----------GTTTGTCCTAACTCCTAGCCCTAACGAACTTCACTAAATTTTATGTGCCAAATAAATTTATAAAA-AAAAA--CATAAAGACATGATGCGG-TGGACTCACAAACTGAAGCATAC---------AAGTTATAAAGTTTTTGAAAGCGAAATAACGTCTTAAATTTTTTTAAATATTATAATCGAATATTTCTACGACTAACAACTGAACTGAATTAACGGAATAATTAATCTATCATATATTGATTTAAGCGAATTGGACCACATCAGGGTACATAGAGTTTGGGGGGCTAGCTAGTGATTAGATCCAAAGTTTTTCTCACCACATATCAATGTGGTCCATCCTAATTAAGTCCATTGTCTACCCCATTTTTGGTGGGGTCCCCAATCTATGAAAAA-TCGAATTCATTTTACTGCTCACTCACCAAAAATGTTCAACAGCCCCACGGTACATTATACAAGTAATAGTAATACAACAACATACATTAAACTTATATAG

>PI280619

AAGTTCAATTATCATCTTCAGGTAAATCGGTAATTAACAAATTTAAATTTCAAATATGTTTTGAATCCTTGTATTTAG-----------GTTTGTCCTAACTCCTAGCCCTAACGAACTTCACTAAATTTTATGTGCCAAATAAATTTATAAAA-AAAAA--CATAAAGACATGATGCGG-TGGACTCACAAACTGAAGCATAC---------AAGTTATAAAGTTTTTGAAAGCGAAATAACGTCTTAAATTTTTTTAAATATTATAATCGAATATTTCTACGACTAACAACTGAACTGAATTAACGGAATAATTAATCTATCATATATTGATTTAAGCGAATTGGACCACATCAGGGTACATAGAGTTTGGGGGGCTAGCTAGTGATTAGATCCAAAGTTTTTCTCACCACATATCAATGTGGTCCATCCTAATTAAGTCCATTGTCTACCCCATTTTTGGTGGGGTCCCCAATCTATGAAAAA-TCGAATTCATTTTACTGCTCACTCACCAAAAATGTTCAACAGCCCCACGGTACATTATACAAGTAATAGTAATACAACAACATACATTAAACTTATATAG

>PI193578

AAGTTCAATTATCATCTTCAGGTAAATCGGTAATTAACAAATTTAAATTTCAAATATGTTTTGAATCCTTGTATTTAG-----------GTTTGTCCTAACTCCTAGCCCTAACGAACTTCACTAAATTTTATGTGCCAAATAAATTTATAAAA-AAAAA--CATAAAGACATGATGCGG-TGGACTCACAAACTGAAGCATAC---------AAGTTATAAAGTTTTTGAAAGCGAAATAACGTCTTAAATTTTTTTAAATATTATAATCGAATATTTCTACGACTAACAACTGAACTGAATTAACGGAATAATTAATCTATCATATATTGATTTAAGCGAATTGGACCACATCAGGGTACATAGAGTTTGGGGGGCTAGCTAGTGATTAGATCCAAAGTTTTTCTCACCACATATCAATGTGGTCCATCCTAATTAAGTCCATTGTCTACCCCATTTTTGGTGGGGTCCCCAATCTATGAAAAA-TCGAATTCATTTTACTGCTCACTCACCAAAAATGTTCAACAGCCCCACGGTACATTATACAAGTAATAGTAATACAACAACATACATTAAACTTATATAG

>PI179450

AAGTTCAATTATCATCTTCAGGTAAATCTGTAATTAACAAATTTAAATATCAAATATGTTTTGAATCCTTGTATTTAG-----------GTTTGTCCTAACTCCTAACCCTAACGAACTTCCCTAAATTTTATGTGCCAAATAAATTTATAAAA-AAAAA--CATAAAGACATGATGCGG-TGGACTCACAAACTGAAGCATAC---------AAGTTATAAAGTTTTTGAAAGCGAAATAACTTCTTAAATTTTTTTAAATATTATAATCGGATATTTCTACGACTAACAACTGAACTGAATTAACGGAATAATTAATCAATCATATATTGATTTAAGCGAATTGGACCACATCAGGGTACATAGAGTTTGGGGGGCTAGCTAGTGATTAGATCCAAAGTTTTTTTCACCACATATCAATGTGGTCCATCCTAATTAAGTCCATTGTCTACCCCATTTTTGGTGGGGTCCCCAATCTATGAAAAA-TCGAATTCATTTTACTGCTCACTCACCAAAAATGTTCAACAGCCCCACGGTACATTATACAAGTAATAGTAATACAACAACATACATTAAACTTATATAG

>PI142775

AAGTTCAATTATCATCTTCAGGTAAATCTTTAATTAACAAATTTAAATATCAAATATGTTTTGAATCCTTGTATTTAG-----------GTTTGTCCTAACTCCTAACCCTAACGAACTTCCCTAAATTTTATGTGCCAAATAAATTTATAAAA-AAAAA--CATAAAGACATGATACGG-TAGACTCACAAACTGAAGCATAC---------AAATTATAAAGTTTTTGAAAGCGAAATAACTTCTTAAATTTTTTTAAATATTATAATCGGATATTTCTACTACTAACAACTGAACTGAATTAACGGAATAATTAATCAATGATATATTGATTTAAGCGAATTGGACCACATCAGGGTACATAGAGTTTGGGGGGCTAGCTAGTGATTAGATCCAAAGTTTTTTTCACCACATATCAATGTGGTCCATCCTAATTAAGTCCATTGTCTACCCCATTTTTGGTGGGGTCCCCAATCTATGAAAAA-TCGAATTCATTTTACTGCTCACTCACCAAAAATGTTCAACAGCCCCACGGTACATTATACAAGTAATAGTAATACAACAACATACATTAAACTTATATAG

>PI184784

AAGTTCAATTATCATCTTCAGGTAAATCTTTAATTAACAAATTTAAATATCAAATATGTTTTGAATCCTTGTATTTAG-----------GTTTGTCCTAACTCCTAACCCTAACGAACTTCCCTAAATTTTATGTGCCAAATAAATTTATAAAA-AAAAA--CATAAAGACATGATACGG-TAGACTCACAAACTGAAGCATAC---------AAATTATAAAGTTTTTGAAAGCGAAATAACTTCTTAAATTTTTTTAAATATTATAATCGGATATTTCTACTACTAACAACTGAACTGAATTAACGGAATAATTAATCAATGATATATTGATTTAAGCGAATTGGACCACATCAGGGTACATAGAGTTTGGGGGGCTAGCTAGTGATTAGATCCAAAGTTTTTTTCACCACATATCAATGTGGTCCATCCTAATTAAGTCCATTGTCTACCCCATTTTTGGTGGGGTCCCCAATCTATGAAAAA-TCGAATTCATTTTACTGCTCACTCACCAAAAATGTTCAACAGCCCCACGGTACATTATACAAGTAATAGTAATACAACAACATACATTAAACTTATATAG

>PI206861

AAGTTCAATTATCATCTTCAGGTAAATCTTTAATTAACAAATTTAAATATCAAATATGTTTTGAATCCTTGTATTTAG-----------GTTTGTCCTAACTCCTAACCCTAACGAACTTCCCTAAATTTTATGTGCCAAATAAATTTATAAAA-AAAAA--CATAAAGACATGATACGG-TAGACTCACAAACTGAAGCATAC---------AAATTATAAAGTTTTTGAAAGCGAAATAACTTCTTAAATTTTTTTAAATATTATAATCGGATATTTCTACTACTAACAACTGAACTGAATTAACGGAATAATTAATCAATGATATATTGATTTAAGCGAATTGGACCACATCAGGGTACATAGAGTTTGGGGGGCTAGCTAGTGATTAGATCCAAAGTTTTTTTCACCACATATCAATGTGGTCCATCCTAATTAAGTCCATTGTCTACCCCATTTTTGGTGGGGTCCCCAATCTATGAAAAA-TCGAATTCATTTTACTGCTCACTCACCAAAAATGTTCAACAGCCCCACGGTACATTATACAAGTAATAGTAATACAACAACATACATTAAACTTATATAG

>PI269825

AAGTTCAATTATCATCTTCAGGTAAATCTTTAATTAACAAATTTAAATATCAAATATGTTTTGAATCCTTGTATTTAG-----------GTTTGTCCTAACTCCTAACCCTAACGAACTTCCCTAAATTTTATGTGCCAAATAAATTTATAAAA-AAAAA--CATAAAGACATGATACGG-TAGACTCACAAACTGAAGCATAC---------AAATTATAAAGTTTTTGAAAGCGAAATAACTTCTTAAATTTTTTTAAATATTATAATCGGATATTTCTACTACTAACAACTGAACTGAATTAACGGAATAATTAATCAATGATATATTGATTTAAGCGAATTGGACCACATCAGGGTACATAGAGTTTGGGGGGCTAGCTAGTGATTAGATCCAAAGTTTTTTTCACCACATATCAATGTGGTCCATCCTAATTAAGTCCATTGTCTACCCCATTTTTGGTGGGGTCCCCAATCTATGAAAAA-TCGAATTCATTTTACTGCTCACTCACCAAAAATGTTCAACAGCCCCACGGTACATTATACAAGTAATAGTAATACAACAACATACATTAAACTTATATAG

>PI331413

AAGTTCAATTATCATCTTCAGGTAAATCTTTAATTAACAAATTTAAATATCAAATATGTTTTGAATCCTTGTATTTAG-----------GTTTGTCCTAACTCCTAACCCTAACGAACTTCCCTAAATTTTATGTGCCAAATAAATTTATAAAA-AAAAA--CATAAAGACATGATACGG-TAGACTCACAAACTGAAGCATAC---------AAATTATAAAGTTTTTGAAAGCGAAATAACTTCTTAAATTTTTTTAAATATTATAATCGGATATTTCTACTACTAACAACTGAACTGAATTAACGGAATAATTAATCAATGATATATTGATTTAAGCGAATTGGACCACATCAGGGTACATAGAGTTTGGGGGGCTAGCTAGTGATTAGATCCAAAGTTTTTTTCACCACATATCAATGTGGTCCATCCTAATTAAGTCCATTGTCTACCCCATTTTTGGTGGGGTCCCCAATCTATGAAAAA-TCGAATTCATTTTACTGCTCACTCACCAAAAATGTTCAACAGCCCCACGGTACATTATACAAGTAATAGTAATACAACAACATACATTAAACTTATATAG

>PI343824

AAGTTCAATTATCATCTTCAGGTAAATCTTTAATTAACAAATTTAAATATCAAATATGTTTTGAATCCTTGTATTTAG-----------GTTTGTCCTAACTCCTAACCCTAACGAACTTCCCTAAATTTTATGTGCCAAATAAATTTATAAAA-AAAAA--CATAAAGACATGATACGG-TAGACTCACAAACTGAAGCATAC---------AAATTATAAAGTTTTTGAAAGCGAAATAACTTCTTAAATTTTTTTAAATATTATAATCGGATATTTCTACTACTAACAACTGAACTGAATTAACGGAATAATTAATCAATGATATATTGATTTAAGCGAATTGGACCACATCAGGGTACATAGAGTTTGGGGGGCTAGCTAGTGATTAGATCCAAAGTTTTTTTCACCACATATCAATGTGGTCCATCCTAATTAAGTCCATTGTCTACCCCATTTTTGGTGGGGTCCCCAATCTATGAAAAA-TCGAATTCATTTTACTGCTCACTCACCAAAAATGTTCAACAGCCCCACGGTACATTATACAAGTAATAGTAATACAACAACATACATTAAACTTATATAG

>JI1987-TT8F2-R7-inT5R

AAGAGGATGACGAAGATGACGAAGTTGAAATCTGGATCCGAAGATGAAACCAATCAAGGTCATAACCAACACGCAACCTCTATAATAGAGGCTGCGGAACCGAGTGAACTCATGCAAATTGAAATGCCCGATGATATTCGGATCGGGTCACCCAACGACGGGTCAAATAATTTAGACTCGGATTTTCATTTGTTGGCCGTTAGTAATCAAGGAAACCCATCAAGACAAATTGACTCATATACAACCGAGAGATGGGGTCCAATCGAAGAACCTCTCGATGATTCACTACAAGTTCAATTATCATCTTCAGGTAAATCTTTAATTAACAAATTTAAATATCAAATATGTTTTGAATCCTTGTATTTAGGTTTGTCCTAACTCCTAACCCTAACGAACTTCCCTAAATTTTATGTGCCAAATAAAT

>PI109866

AAGTTCAATTATCATCTTCAGGTAAATCTTTAATTAACAAATTTAAATATCAAATATGTTTTGAATCCTTGTATTTAG-----------GTTTGTCCTAACTCCTAACCCTAACGAACTTCCCTAAATTTTATGTGCCAAATAAATTTATAAAA-AAAAAA-CATAAAGACATGATACGG-TAGACTCACAAACTGAAGCATAC---------AAATTATAAAGTTTTTGAAAGCGAAATAACTTCTTAAATTTTTTTAAATATTATAATCGGATATTTCTACGACTAACAACTGAACTGAATTAACGGAATAATTAATCAATCATATATTGATTTAAGCGAATTGGACCACATCAGGGTACATAGAGTTTGGGGGGCTAGCTAGTGATTAGATCCAAAGTTTTTTTCACCACATATCAATGTGGTCCATCCTAATTAAGTCCATTGTCTACCCCATTTTTGGTGGGGTCCCCAATCTATGAAAAAATCGAATTCATTTTACTGCTCACTCACCAAAAATGTTCAACAGCCCCACGGTACATTATACAAGTAATAGTAATACAACAACATACATTAAACTTATATAG

>PI137118

AAGTTCAATTATCATCTTCAGGTAAATCTTTAATTAACAAATTTAAATATCAAATATGTTTTGAATCCTTGTATTTAG-----------GTTTGTCCTAACTCCTAACCCTAACGAACTTCCCTAAATTTTATGTGCCAAATAAATTTATAAAA-AAAAA--CATAAAGACATGATACGG-TAGACTCACAAACTGAAGCATAC---------AAATTATAAAGTTTTTGAAAGCGAAATAACTTCTTAAATTTTTTTAAATATTATAATCGGATATTTCTACGACTAACAACTGAACTGAATTAACGGAATAATTAATCAATGATATATTGATTTAAGCGAATTGGACCACATCAGGGTACATAGAGTTTGGGGGGCTAGCTAGTGATTAGATCCAAAGTTTTTTTCACCACATATCAATGTGGTCCATCCTAATTAAGTCCATTGTCTACCCCATTTTTGGTGGGGTCCCCAATCTATGAAAAA-TCGAATTCATTTTACTGCTCACTCACCAAAAATGTTCAACAGCCCCACGGTACATTATACAAGTAATAGTAATACAACAACATACATTAAACTTATATAG

>PI137119

AAGTTCAATTATCATCTTCAGGTAAATCTTTAATTAACAAATTTAAATATCAAATATGTTTTGAATCCTTGTATTTAG-----------GTTTGTCCTAACTCCTAACCCTAACGAACTTCCCTAAATTTTATGTGCCAAATAAATTTATAAAA-AAAAA--CATAAAGACATGATACGG-TAGACTCACAAACTGAAGCATAC---------AAATTATAAAGTTTTTGAAAGCGAAATAACTTCTTAAATTTTTTTAAATATTATAATCGGATATTTCTACTACTAACAACTGAACTGAATTAACGGAATAATTAATCAATGATATATTGATTTAAGCGAATTGGACCACATCAGGGTACATAGAGTTTGGGGGGCTAGCTAGTGATTAGATCCAAAGTTTTTTTCACCACATATCAATGTGGTCCATCCTAATTAAGTCCATTGTCTACCCCATTTTTGGTGGGGTCCCCAATCTATGAAAAA-TCGAATTCATTTTACTGCTCACTCACCAAAAATGTTCAACAGCCCCACGGTACATTATACAAGTAATAGTAATACAACAACATACATTAAACTTATATAG

>PI140298

AAGTTCAATTATCATCTTCAGGTAAATCTTTAATTAACAAATTTAAATATCAAATATGTTTTGAATCCTTGTATTTAG-----------GTTTGTCCTAACTCCTAACCCTAACGAACTTCCCTAAATTTTATGTGCCAAATAAATTTATAAAA-AAAAA--CATAAAGACATGATACGG-TAGACTCACAAACTGAAGCATAC---------AAATTATAAAGTTTTTGAAAGCGAAATAACCTCTTAAATTTTTTTAAATATTATAATCGGATATTTCTACGACTAACAACTGAACTGAATTAACGGAATAATTAATCAATGATATATTGATTTAAGCGAATTGGACCACATCAGGGTACATAGAGTTTGGGGGGCTAGCTAGTGATTAGATCCAAAGTTTTTTTCACCACATATCAATGTGGTCCATCCTAATTAAGTCCATTGTCTACCCCATTTTTGGTGGGGTCCCCAATCTATGAAAAA-TCGAATTCATTTTACTGCTCACTCACCAAAAATGTTCAACAGCCCCACGGTACATTATACAAGTAATAGTAATACAACAACATACATTAAACTTATATAG

>PI155109

AAGTTCAATTATCATCTTCAGGTAAATCTTTAATTAACAAATTTAAATATCAAATATGTTTTGAATCCTTGTATTTAG-----------GTTTGTCCTAACTCCTAACCCTAACGAACTTCCCTAAATTTTATGTGCCAAATAAATTTATAAAA-AAAAA--CATAAAGACATGATACGG-TAGACTCACAAACTGAAGCATAC---------AAATTATAAAGTTTTTGAAAGCGAAATAACTTCTTAAATTTTTTTAAATATTATAATCGGATATTTCTACGACTAACAACTGAACTGAATTAACGGAATAATTAATCAATGATATATTGATTTAAGCGAATTGGACCACATCAGGGTACATAGAGTTTGGGGGGCTAGCTAGTGATTAGATCCAAAGTTTTTTTCACCACATATCAATGTGGTCCATCCTAATTAAGTCCATTGTCTACCCCATTTTTGGTGGGGTCCCCAATCTATGAAAAA-TCGAATTCATTTTACTGCTCACTCACCAAAAATGTTCAACAGCCCCACGGTACATTATACAAGTAATAGTAATACAACAACATACATTAAACTTATATAG

>PI156647

AAGTTCAATTATCATCTTCAGGTAAATCTTTAATTAACAAATTTAAATATCAAATATGTTTTGAATCCTTGTATTTAG-----------GTTTGTCCTAACTCCTAACCCTAACGAACTTCCCTAAATTTTATGTGCCAAATAAATTTATAAAA-AAAAA--CATAAAGACATGATACGG-TAGACTCACAAACTGAAGCATAC---------AAATTATAAAGTTTTTGAAAGCGAAATAACCTCTTAAATTTTTTTAAATATTATAATCGGATATTTCTACGACTAACAACTGAACTGAATTAACGGAATAATTAATCAATGATATATTGATTTAAGCGAATTGGACCACATCAGGGTACATAGAGTTTGGGGGGCTAGCTAGTGATTAGATCCAAAGTTTTTTTCACCACATATCAATGTGGTCCATCCTAATTAAGTCCATTGTCTACCCCATTTTTGGTGGGGTCCCCAATCTATGAAAAA-TCGAATTCATTTTACTGCTCACTCACCAAAAATGTTCAACAGCCCCACGGTACATTATACAAGTAATAGTAATACAACAACATACATTAAACTTATATAG

>PI164972

AAGTTCAATTATCATCTTCAGGTAAATCTTTAATTAACAAATTTAAATATCAAATATGTTTTGAATCCTTGTATTTAG-----------GTTTGTCCTAACTCCTAACCCTAACGAACTTCCCTAAATTTTATGTGCCAAATAAATTTATAAAA-AAAAA--CATAAAGACATGATACGG-TAGACTCACAAACTGAAGCATAC---------AAATTATAAAGTTTTTGAAAGCGAAATAACTTCTTAAATTTTTTTAAATATTATAATCGGATATTTCTACGACTAACAACTGAACTGAATTAACGGAATAATTAATCTATCATATATTGATTTAAGCGAATTGGACCACATCAGGGTACATAGAGTTTGGGGGGCTAGCTAGTGATTAGATCCAAAGTTTTTTTCACCACATATCAATGTGGTCCATCCTAATTAAGTCCATTGTCTACCCCATTTTTGGTGGGGTCCCCAATCTATGAAAAA-TCGAATTCATTTTACTGCTCACTCACCAAAAATGTTCAACAGCCCCACGGTACATTATACAAGTAATAGTAATACAACAACATACATTAAACTTATATAG

>PI174320

AAGTTCAATTATCATCTTCAGGTAAATCTTTAATTAACAAATTTAAATATCAAATATGTTTTGAATCCTTGTATTTAG-----------GTTTGTCCTAACTCCTAACCCTAACGAACTTCCCTAAATTTTATGTGCCAAATAAATTTATAAAA-AAAAA--CATAAAGACATGATACGG-TAGACTCACAAACTGAAGCATAC---------AAATTATAAAGTTTTTGAAAGCGAAATAACCTCTTAAATTTTTTTAAATATTATAATCGGATATTTCTACGACTAACAACTGAACTGAATTAACGGAATAATTAATCAATGATATATTGATTTAAGCGAATTGGACCACATCAGGGTACATAGAGTTTGGGGGGCTAGCTAGTGATTAGATCCAAAGTTTTTTTCACCACATATCAATGTGGTCCATCCTAATTAAGTCCATTGTCTACCCCATTTTTGGTGGGGTCCCCAATCTATGAAAAA-TCGAATTCATTTTACTGCTCACTCACCAAAAATGTTCAACAGCCCCACGGTACATTATACAAGTAATAGTAATACAACAACATACATTAAACTTATATAG

>PI180329

AAGTTCAATTATCATCTTCAGGTAAATCTTTAATTAACAAATTTAAATATCAAATATGTTTTGAATCCTTGTATTTAG-----------GTTTGTCCTAACTCCTAACCCTAACGAACTTCCCTAAATTTTATGTGCCAAATAAATTTATAAAA-AAAAA--CATAAAGACATGATACGG-TAGACTCACAAACTGAAGCATAC---------AAATTATAAAGTTTTTGAAAGCGAAATAACTTCTTAAATTTTTTTAAATATTATAATCGGATATTTCTACTACTAACAACTGAACTGAATTAACGGAATAATTAATCAATGATATATTGATTTAAGCGAATTGGACCACATCAGGGTACATAGAGTTTGGGGGGCTAGCTAGTGATTAGATCCAAAGTTTTTTTCACCACATATCAATGTGGTCCATCCTAATTAAGTCCATTGTCTACCCCATTTTTGGTGGGGTCCCCAATCTATGAAAAA-TCGAATTCATTTTACTGCTCACTCACCAAAAATGTTCAACAGCCCCACGGTACATTATACAAGTAATAGTAATACAACAACATACATTAAACTTATATAG

>PI180702

AAGTTCAATTATCATCTTCAGGTAAATCTTTAATTAACAAATTTAAATATCAAATATGTTTTGAATCCTTGTATTTAG-----------GTTTGTCCTAACTCCTAACCCTAACGAACTTCCCTAAATTTTATGTGCCAAATAAATTTATAAAA-AAAAAA-CATAAAGACATGATACGG-TAGACTCACAAACTGAAGCATAC---------AAATTATAAAGTTTTTGAAAGCGAAATAACTTCTTAAATTTTTTTAAATATTATAATCGGATATTTCTACGACTAACAACTGAACTGAATTAACGGAATAATTAATCAATCATATATTGATTTAAGCGAATTGGACCACATCAGGGTACATAGAGTTTGGGGGGCTAGCTAGTGATTAGATCCAAAGTTTTTTTCACCACATATCAATGTGGTCCATCCTAATTAAGTCCATTGTCTACCCCATTTTTGGTGGGGTCCCCAATCTATGAAAAA-TCGAATTCATTTTACTGCTCACTCACCAAAAATGTTCAACAGCCCCACGGTACATTATACAAGTAATAGTAATACAACAACATACATTAAACTTATATAG

>PI197990

AAGTTCAATTATCATCTTCAGGTAAATCTTTAATTAACAAATTTAAATATCAAATATGTTTTGAATCCTTGTATTTAG-----------GTTTGTCCTAACTCCTAACCCTAACGAACTTCCCTAAATTTTATGTGCCAAATAAATTTATAAAA-AAAAA--CATAAAGACATGATACGG-TAGACTCACAAACTGAAGCATAC---------AAATTATAAAGTTTTTGAAAGCGAAATAACCTCTTAAATTTTTTTAAATATTATAATCGGATATTTCTACGACTAACAACTGAACTGAATTAACGGAATAATTAATCAATGATATATTGATTTAAGCGAATTGGACCACATCAGGGTACATAGAGTTTGGGGGGCTAGCTAGTGATTAGATCCAAAGTTTTTTTCACCACATATCAATGTGGTCCATCCTAATTAAGTCCATTGTCTACCCCATTTTTGGTGGGGTCCCCAATCTATGAAAAA-TCGAATTCATTTTACTGCTCACTCACCAAAAATGTTCAACAGCCCCACGGTACATTATACAAGTAATAGTAATACAACAACATACATTAAACTTATATAG

>PI198072

AAGTTCAATTATCATCTTCAGGTAAATCTTTAATTAACAAATTTAAATATCAAATATGTTTTGAATCCTTGTATTTAG-----------GTTTGTCCTAACTCCTAACCCTAACGAACTTCCCTAAATTTTATGTGCCAAATAAATTTATAAAA-AAAAA--CATAAAGACATGATACGG-TAGACTCACAAACTGAAGCATAC---------AAATTATAAAGTTTTTGAAAGCGAAATAACTTCTTAAATTTTTTTAAATATTATAATCGGATATTTCTACTACTAACAACTGAACTGAATTAACGGAATAATTAATCAATGATATATTGATTTAAGCGAATTGGACCACATCAGGGTACATAGAGTTTGGGGGGCTAGCTAGTGATTAGATCCAAAGTTTTTTTCACCACATATCAATGTGGTCCATCCTAATTAAGTCCATTGTCTACCCCATTTTTGGTGGGGTCCCCAATCTATGAAAAA-TCGAATTCATTTTACTGCTCACTCACCAAAAATGTTCAACAGCCCCACGGTACATTATACAAGTAATAGTAATACAACAACATACATTAAACTTATATAG

>PI204306

AAGTTCAATTATCATCTTCAGGTAAATCTTTAATTAACAAATTTAAATATCAAATATGTTTTGAATCCTTGTATTTAG-----------GTTTGTCCTAACTCCTAACCCTAACGAACTTCCCTAAATTTTATGTGCCAAATAAATTTATAAAAGAAAAA--CATAAAGACATGACGCGG-TGGACTCACAAACTGAAGCATAC---------AAGTTATAAAGTTTTTGAAAGCGAAATAACTTCTTAAATTTGTTTAAATATTATAATCGGATATTTCTACGACTAACAACTGAACTGAATTAACGGAATAATTAATCAATCATATATTGATTTAAGCGAATTGGACCACATCAGGGTACATAGAGTTTGGGGGGCTAGCTAGTGATTAGATCCAAAGTTTTTTTCACCACATATCAATGTGGTCCATCCTAATTAAGTCCATTGTCTACCCCATTTTTGGTGGGGTCCCCAATCTATGAAAAA-TCGAATTCATTTTACTGCTCACTCACCAAAAATGTTCAACAGCCCCACGGTACATTATACAAGTAATAGTAATACAACAACATACATTAAACTTATATAG

>PI343958

AAGTTCAATTATCATCTTCAGGTAAATCTTTAATTAACAAATTTAAATATCAAATATGTTTTGAATCCTTGTATTTAG-----------GTTTGTCCTAACTCCTAACCCTAACGAACTTCCCTAAATTTTATGTGCCAAATAAATTTATAAAA-AAAAA--CATAAAGACATGATACGG-TAGACTCACAAACTGAAGCATAC---------AAATTATAAAGTTTTTGAAAGCGAAATAACCTCTTAAATTTTTTTAAATATTATAATCGGATATTTCTACGACTAACAACTGAACTGAATTAACGGAATAATTAATCAATGATATATTGATTTAAGCGAATTGGACCACATCAGGGTACATAGAGTTTGGGGGGCTAGCTAGTGATTAGATCCAAAGTTTTTTTCACCACATATCAATGTGGTCCATCCTAATTAAGTCCATTGTCTACCCCATTTTTGGTGGGGTCCCCAATCTATGAAAAA-TCGAATTCATTTTACTGCTCACTCACCAAAAATGTTCAACAGCCCCACGGTACATTATACAAGTAATAGTAATACAACAACATACATTAAACTTATATAG

>PI198074

AAGTTCAATTATCATCTTCAGGTAAATCTTTAATTAACAAATTTAAATATCAAATATGTTTTGAATCCTTGTATTTAG-----------GTTTGTCCTAACTCATAACCCTAACGAACTTCCCTAAATTTTATGTGCCAAATAAATTTATAAAA-AAAAA--CATAAAGACATGATACGG-TAGACTCACAAACTAAAGCATAC---------AAATTATAAAGTTTTTGAAAGCGAAATAACTTCTTAAATTTTTTTAAATATTATAATCGGATATTTCTGCGACTAACAACTGAACTGAATTAACGGAATAATTAATCAATCATATATTGATTTAAGCGAATTGGACCACATCAGGGTACATAGAGTTTGGGGGGCTAGCTAGTGATTAGATCCAAAGTTTTTTTCACCACATATCAATGTGGTCCATCCTAATTAAGTCCATTGTCTACCCCATTTTTGGTGGGGTCCCCAATCTATGAAAAA-TCGAATTCATTTTACTGCTCACTCACCAAAAATGTTCAACAGCCCCACGGTACATTATACAAGTAATAGTAATACAACAACATACATTAAACTTATATAG

>PI266070

AAGTTCAATTATCATCTTCAGGTAAATCTTTAATTAACAAATTTAAATATCAAATATGTTTTGAATCCTTGTATTTAG-----------GTTTGTCCTAACTCATAACCCTAACGAACTTCCCTAAATTTTATGTGCCAAATAAATTTATAAAA-AAAAA--CATAAAGACATGATACGG-TAGACTCACAAACTAAAGCATAC---------AAATTATAAAGTTTTTGAAAGCGAAATAACTTCTTAAATTTTTTTAAATATTATAATCGGATATTTCTGCGACTAACAACTGAACTGAATTAACGGAATAATTAATCAATCATATATTGATTTAAGCGAATTGGACCACATCAGGGTACATAGAGTTTGGGGGGCTAGCTAGTGATTAGATCCAAAGTTTTTTTCACCACATATCAATGTGGTCCATCCTAATTAAGTCCATTGTCTACCCCATTTTTGGTGGGGTCCCCAATCTATGAAAAA-TCGAATTCATTTTACTGCTCACTCACCAAAAATGTTCAACAGCCCCACGGTACATTATACAAGTAATAGTAATACAACAACATACATTAAACTTATATAG

>PI117998

AAGTTCAATTATCATCTTCAGGTAAATCTTTAATTAACAAATTTAAATATCAAATATGTTTTGAATCCTTGTATTTAG-----------GTTTGTCCTAACTCATAACCCTAACGAACTTCCCTAAATTTTATGTGCCAAATAAATTTATAAAA-AAAAA--CATAAAGACATGATACGG-TAGACTCACAAACTAAAGCATAC---------AAATTATAAAGTTTTTGAAAGCGAAATAACTTCTTAAATTTTTTTAAATATTATAATCGGATATTTCTGCGACTAACAACTGAACTGAATTAACGGAATAATTAATCAATCATATATTGATTTAAGCGAATTGGACCACATCAGGGTACATAGAGTTTGGGGGGCTAGCTAGTGATTAGATCCAAAGTTTTTTTCACCACATATCAATGTGGTCCATCCTAATTAAGTCCATTGTCTACCCCATTTTTGGTGGGGTCCCCAATCTATGAAAAA-TCGAATTCATTTTACTGCTCACTCACCAAAAATGTTCAACAGCCCCACGGTACATTATACAAGTAATAGTAATACAACAACATACATTAAACTTATATAG

>PI179451

AAGTTCAATTATCATCTTCAGGTAAATCTTTAATTAACAAATTTAAATATCAAATATGTTTTGAATCCTTGTATTTAG-----------GTTTGTCCTAACTCATAACCCTAACGAACTTCCCTAAATTTTATGTGCCAAATAAATTTATAAAA-AAAAA--CATAAAGACATGATACGG-TAGACTCACAAACTAAAGCATAC---------AAATTATAAAGTTTTTGAAAGCGAAATAACTTCTTAAATTTTTTTAAATATTATAATCGGATATTTCTGCGACTAACAACTGAACTGAATTAACGGAATAATTAATCAATCATATATTGATTTAAGCGAATTGGACCACATCAGGGTACATAGAGTTTGGGGGGCTAGCTAGTGATTAGATCCAAAGTTTTTTTCACCACATATCAATGTGGTCCATCCTAATTAAGTCCATTGTCTACCCCATTTTTGGTGGGGTCCCCAATCTATGAAAAA-TCGAATTCATTTTACTGCTCACTCACCAAAAATGTTCAACAGCCCCACGGTACATTATACAAGTAATAGTAATACAACAACATACATTAAACTTATATAG

>PI203064

AAGTTCAATTATCATCTTCAGGTAAATCTTTAATTAACAAATTTAAATATCAAATATGTTTTGAATCCTTGTATTTAG-----------GTTTGTCCTAACTCATAACCCTAACGAACTTCCCTAAATTTTATGTGCCAAATAAATTTATAAAA-AAAAA--CATAAAGACATGATACGG-TAGACTCACAAACTAAAGCATAC---------AAATTATAAAGTTTTTGAAAGCGAAATAACTTCTTAAATTTTTTTAAATATTATAATCGGATATTTCTGCGACTAACAACTGAACTGAATTAACGGAATAATTAATCAATCATATATTGATTTAAGCGAATTGGACCACATCAGGGTACATAGAGTTTGGGGGGCTAGCTAGTGATTAGATCCAAAGTTTTTTTCACCACATATCAATGTGGTCCATCCTAATTAAGTCCATTGTCTACCCCATTTTTGGTGGGGTCCCCAATCTATGAAAAA-TCGAATTCATTTTACTGCTCACTCACCAAAAATGTTCAACAGCCCCACGGTACATTATACAAGTAATAGTAATACAACAACATACATTAAACTTATATAG

>PI164971

AAATTCAATTATCATCTTCAGGTAAATCGGTAATTAACAAATTTAAATTTCAAATATGTTTTGAATCCTTGTATTTAG-----------GTTTGTCCTAACTCTTAACCCTAACGAACGTCCCTAAATTTTATGTGCCAAATAAATTTATAAAAGAAAAA--CATAAAGACATGACGCGG-TGGACTCACAAACTGAAGCATAC---------AAGTTATAAAGTTTTTGAAAGCGAAATAACTTCTTAAATTTGTTTAAATATTATAATCGGATATTTCTACGACTAACAACTGAACTGAATTAACGGAATAATTAATCAATCATATATTGATTTAAGCGAATTGGACCACATCAGGGTACATAGAGTTTGGGGGGCTAGCTAGTGATTAGATCCAAAGTTTTTTTCACCACATATCAATGTGGTCCATCCTAATTAAGTCCATTGTCTACCCCATTTTTGGTGGGGTCCCCAATCTATGAAAAA-TCGAATTCATTTTACTGCTCACTCACCAAAAATGTTCAACAGCCCCACGGTACATTATACAAGTAATAGTAATACAACAACATACATTAAACTTATATAG

>PI188698

AAATTCAATTATCATCTTCAGGTAAATCGGTAATTAACAAATTTAAATTTCAAATATGTTTTGAATCCTTGTATTTAG-----------GTTTGTCCTAACTCTTAACCCTAACGAACGTCCCTAAATTTTATGTGCCAAATAAATTTATAAAAGAAAAA--CATAAAGACATGACGCGG-TGGACTCACAAACTGAAGCATAC---------AAGTTATAAAGTTTTTGAAAGCGAAATAACTTCTTAAATTTGTTTAAATATTATAATCGGATATTTCTACGACTAACAACTGAACTGAATTAACGGAATAATTAATCAATCATATATTGATTTAAGCGAATTGGACCACATCAGGGTACATAGAGTTTGGGGGGCTAGCTAGTGATTAGATCCAAAGTTTTTTTCACCACATATCAATGTGGTCCATCCTAATTAAGTCCATTGTCTACCCCATTTTTGGTGGGGTCCCCAATCTATGAAAAA-TCGAATTCATTTTACTGCTCACTCACCAAAAATGTTCAACAGCCCCACGGTACATTATACAAGTAATAGTAATACAACAACATACATTAAACTTATATAG

>PI242027

AAATTCAATTATCATCTTCAGGTAAATCGGTAATTAACAAATTTAAATTTCAAATATGTTTTGAATCCTTGTATTTAG-----------GTTTGTCCTAACTCTTAACCCTAACGAACGTCCCTAAATTTTATGTGCCAAATAAATTTATAAAAGAAAAA--CATAAAGACATGACGCGG-TGGACTCACAAACTGAAGCATAC---------AAGTTATAAAGTTTTTGAAAGCGAAATAACTTCTTAAATTTGTTTAAATATTATAATCGGATATTTCTACGACTAACAACTGAACTGAATTAACGGAATAATTAATCAATCATATATTGATTTAAGCGAATTGGACCACATCAGGGTACATAGAGTTTGGGGGGCTAGCTAGTGATTAGATCCAAAGTTTTTTTCACCACATATCAATGTGGTCCATCCTAATTAAGTCCATTGTCTACCCCATTTTTGGTGGGGTCCCCAATCTATGAAAAA-TCGAATTCATTTTACTGCTCACTCACCAAAAATGTTCAACAGCCCCACGGTACATTATACAAGTAATAGTAATACAACAACATACATTAAACTTATATAG

>PI269818

AAATTCAATTATCATCTTCAGGTAAATCGGTAATTAACAAATTTAAATTTCAAATATGTTTTGATTCCTTGTATTTAG-----------GTTTGTCCTAACTCCTAACCCTAACAAACTTCCCTAAATTTTATGTGCCAAGTAAATTTATAAAA-AAAAA--CATAAAGACATGATGCGG-TGGACTCACAAACTGAAGCATAC---------AAGTTATAAAGTTTTTGAAAGCGAAATAACTTCTTTAATTTTTTTAAATATTATAATCGAATATTTCTACGACTAACAACTGAACTGAATTAACGGAATAATTAATCTATCATATATTGATTTAAGTGAATTGGACCACATCAGGGTACATAGAGTTTGGGGGGCTAGCTAGTGATTAGATCCAAAGTTTTTCTCACCACATATCAATGTGGTCCATCCTAATTAAGT

>PI162909

AAATTCAATTATCATCTTCAGGTAAATCGGTAATTAACAAATTTAAATTTCAAATATGTTTTGATTCCTTGTATTTAG-----------GTTTGTCCTAACTCCTAACCCTAACAAACTTCCCTAAATTTTATGTGCCAAGTAAATTTATAAAA-AAAAA--CATAAAGACATGATGCGG-TGGACTCACAAACTGAAGCATAC---------AAGTTATAAAGTTTTTGAAAGCGAAATAACTTCTTAAATTTTTTTAAATATTATAATCGAATATTTCTACGACTAACAACTGAACTGAATTAACGGAATAATTAATCTATCATATATTGATTTAAGTGAATTGGACCACATCAGGGTACATAGAGTTTGGGGGGCTAGCTAGTGATTAGATCCAAAGTTTTTCTCACCACATATCAATGTGGTCCATCCTAATTAAGTCCATTGTCTACCCCATTTTTGGTGGGGTCCCCAATCTATGAAAAA-TCGAATTCATTTTACTGCTCACTCACCAAAAATGTTCAACAGCCCCACGGTACATTATACAAGTAATAGTAATACAACAACATACATTAAACTTATATAG

>PI179722

AAATTCAATTATCATCTTCAGGTAAATCGGTAATTAACAAATTTAAATTTCAAATATGTTTTGATTCCTTGTATTTAG-----------GTTTGTCCTAACTCCTAACCCTAACAAACTTCCCTAAATTTTATGTGCCAAGTAAATTTATAAAA-AAAAA--CATAAAGACATGATGCGG-TGGACTCACAAACTGAAGCATAC---------AAGTTATAAAGTTTTTGAAAGCGAAATAACTTCTTAAATTTTTTTAAATATTATAATCGAATATTTCTACGACTAACAACTGAACTGAATTAACGGAATAATTAATCTATCATATATTGATTTAAGTGAATTGGACCACATCAGGGTACATAGAGTTTGGGGGGCTAGCTAGTGATTAGATCCAAAGTTTTTCTCACCACATATCAATGTGGTCCATCCTAATTAAGTCCATTGTCTACCCCATTTTTGGTGGGGTCCCCAATCTATGAAAAA-TCGAATTCATTTTACTGCTCACTCACCAAAAATGTTCAACAGCCCCACGGTACATTATACAAGTAATAGTAATACAACAACATACATTAAACTTATATAG

>PI165949

AAATTCAATTATCATCTTCAGGTAAATCGGTAATTAACAAATTTAAATTTCAAATATGTTTTGATTCCTTGTATTTAG-----------GTTTGTCCTAACTCCTAACCCTAACAAACTTCCCTAAATTTTATGTGCCAAGTAAATTTATAAAA-AAAAA--CATAAAGACATGATGCGG-TGGACTCACAAACTGAAGCATAC---------AAGTTATAAAGTTTTTGAAAGCGAAATAACTTCTTAAATTTTTTTAAATATTATAATCGAATATTTCTACGACTAACAACTGAACTGAATTAACGGAATAATTAATCTATCATATATTGATTTAAGTGAATTGGACCACATCAGGGTACATAGAGTTTGGGGGGCTAGCTAGTGATTAGATCCAAAGTTTTTCTCACCACATATCAATGTGGTCCATCCTAATTAAGTCCATTGTCTACCCCATTTTTGGTGGGGTCCCCAATCTATGAAAAA-TCGAATTCATTTTACTGCTCACTCACCAAAAATGTTCAACAGCCCCACGGTACATTATACAAGTAATAGTAATACAACAACATACATTAAACTTATATAG

>PI166084

AAATTCAATTATCATCTTCAGGTAAATCGGTAATTAACAAATTTAAATTTCAAATATGTTTTGATTCCTTGTATTTAG-----------GTTTGTCCTAACTCCTAACCCTAACAAACTTCCCTAAATTTTATGTGCCAAGTAAATTTATAAAA-AAAAA--CATAAAGACATGATGCGG-TGGACTCACAAACTGAAGCATAC---------AAGTTATAAAGTTTTTGAAAGCGAAATAACTTCTTAAATTTTTTTAAATATTATAATCGAATATTTCTACGACTAACAACTGAACTGAATTAACGGAATAATTAATCTATCATATATTGATTTAAGTGAATTGGACCACATCAGGGTACATAGAGTTTGGGGGGCTAGCTAGTGATTAGATCCAAAGTTTTTCTCACCACATATCAATGTGGTCCATCCTAATTAAGTCCATTGTCTACCCCATTTTTGGTGGGGTCCCCAATCTATGAAAAA-TCGAATTCATTTTACTGCTCACTCACCAAAAATGTTCAACAGCCCCACGGTACATTATACAAGTAATAGTAATACAACAACATACATTAAACTTATATAG

>PI166159

AAATTCAATTATCATCTTCAGGTAAATCGGTAATTAACAAATTTAAATTTCAAATATGTTTTGATTCCTTGTATTTAG-----------GTTTGTCCTAACTCCTAACCCTAACAAACTTCCCTAAATTTTATGTGCCAAGTAAATTTATAAAA-AAAAA--CATAAAGACATGATGCGG-TGGACTCACAAACTGAAGCATAC---------AAGTTATAAAGTTTTTGAAAGCGAAATAACTTCTTAAATTTTTTTAAATATTATAATCGAATATTTCTACGACTAACAACTGAACTGAATTAACGGAATAATTAATCTATCATATATTGATTTAAGTGAATTGGACCACATCAGGGTACATAGAGTTTGGGGGGCTAGCTAGTGATTAGATCCAAAGTTTTTCTCACCACATATCAATGTGGTCCATCCTAATTAAGTCCATTGTCTACCCCATTTTTGGTGGGGTCCCCAATCTATGAAAAA-TCGAATTCATTTTACTGCTCACTCACCAAAAATGTTCAACAGCCCCACGGTACATTATACAAGTAATAGTAATACAACAACATACATTAAACTTATATAG

>PI174921

AAATTCAATTATCATCTTCAGGTAAATCGGTAATTAACAAATTTAAATTTCAAATATGTTTTGATTCCTTGTATTTAG-----------GTTTGTCCTAACTCCTAACCCTAACAAACTTCCCTAAATTTTATGTGCCAAGTAAATTTATAAAA-AAAAA--CATAAAGACATGATGCGG-TGGACTCACAAACTGAAGCATAC---------AAGTTATAAAGTTTTTGAAAGCGAAATAACTTCTTAAATTTTTTTAAATATTATAATCGAATATTTCTACGACTAACAACTGAACTGAATTAACGGAATAATTAATCTATCATATATTGATTTAAGTGAATTGGACCACATCAGGGTACATAGAGTTTGGGGGGCTAGCTAGTGATTAGATCCAAAGTTTTTCTCACCACATATCAATGTGGTCCATCCTAATTAAGTCCATTGTCTACCCCATTTTTGGTGGGGTCCCCAATCTATGAAAAA-TCGAATTCATTTTACTGCTCACTCACCAAAAATGTTCAACAGCCCCACGGTACATTATACAAGTAATAGTAATACAACAACATACATTAAACTTATATAG

>PI164779

AAATTCAATTATCATCTTCAGGTAAATCGGTAATTAACAAATTTAAATTTCAAATATGTTTTGATTCCTTGTATTTAG-----------GTTTGTCCTAACTCCTAACCCTAACAAACTTCCCTAAATTTTATGTGCCAAGTAAATTTATAAAA-AAAAA--CATAAAGACATGATGCGG-TGGACTCACAAACTGAAGCATAC---------AAGTTATAAAGTTTTTGAAAGCGAAATAACTTCTTAAATTTTTTTAAATATTATAATCGAATATTTCTACGACTAACAACTGAACTGAATTAACGGAATAATTAATCTATCATATATTGATTTAAGTGAATTGGACCACATCAGGGTACATAGAGTTTGGGGGGCTAGCTAGTGATTAGATCCAAAGTTTTTCTCACCACATATCAATGTGGTCCATCCCAATTAAGTCCATTGTCTACCCCATTTTTGGTGGGGTCCCCAATCTATGAAAAA-TCGAATTCATTTTACTGCTCACTCACCAAAAATGTTCAACAGCCCCACGGTACATTATACAAGTAATAGTAATACAACAACATACATTAAACTTATATAG

>PI271033

AAGTTCAATTATCATCTTCAGGTAAATCGGTAATTAACAAATTTAAATTTCAAATATATTTTGATTCCTTGTATTTAG-----------GTTTGTCCTAACTCCTAACCCTAACGAGCTTCCCTAAATTTTATGTGCCAAACAAATTTATAAAA-AAAAAA-CATAAACACATGATACAG-TGGACTTACAAACTAAAGCATAA---------AAGTTATAAAGTTTTTGAAAGCGAAATAACTTCTTAAAATTTTTTAAATATTATAATCGGATATTTCTACGGCTAACAACTGAACTGAATTAACGGAACAATTAATCAATCATATATTGATTTAAGCGAATTGGACCACATCAGG-TACATAGAGTTTGGGGGGCTAGCTAGTGATTAGATCCAAACTCTTTTTCACCACATATCAATGTGGTCCATCCTAATTAAGTCCATTGTCTACCCCATTTTTGGTGGGGTCCCCAATCTATGAAAAA-TCGAATTCTTTTTACTGCTCAGTCACCAAAAATGTTCAACAGCCCCACGGTACATTATACAAGTAATAGTAATACAACAACATACATTAAACTTATATAG

>PI273209

AAGTTCAATTATCATCTTCAGGTAAATCGGTAATTAACAAATTTAAATTTCAAATATATTTTGATTCCTTGTATTTAGGTTGTATTTAGGTTTGTCCTAACTCCTAACCCTAACGAGCTTCCCTAAATTTTATGTGCCAAACAAATTTATAAAA-AAAAAAACATAAACA------ACAG-TGGACTCACAAACTAAAGCATAC---------AAGTTATAAAGTTTTTGAAAGCGAAATAACTTCTTAAAAATTTTTAAATATTATAATCGGATATTTCTACGGCTAACAACTGAACTGAATTAACGGAACAATTAATCAATGATATATTGATTTAAGCGAATTGGACCACATCAGG-TACATAGAGTTTGGGGGGCTAGCTAGTGATTAGATCCAAACTCTTTTTCACCACATATCAATGTGGTCCATCCTAATTAAGTCCATTGTCTACCCCATTTTTGGTGGGGTCCCCAATCTATGAAAAA-TCGAATTCATTTTACTGCTCAGTCACCAAAAATGTTCAACAGCCCCACGGTACATTATACAAGTAATAGTAATACAACAACATACATTAAACTTATATAG

>PI118501

AAGTTCAATTATCATCTTCAGATAAATCGGTAATTAACAAATTTAAATTTCAAATATGTTTTGAATCCTTGTATTTAG-----------GTTTGTCCTAACTCCTAGCCCTAACGAACTTCACTAAATTTTATGTGCCAAATAAATTTATAAAA-AAAAA--CATAAAGACATGATGCGG-TGGACTCACAAACTGAAGCATAC---------AAGTTATAAAGTTTTTGAAAGCGAAATAACGTCTTAAATTTTTTTAAATATTATAATCGAATATTTCTACGACTAACAACTGAACTGAATTAACGGAATAATTAATCTATCATATATTGATTTAAGCGAATTGGACCACATCAGGGTACATAGAGTTTGGGGGGCTAGCTAGTGATTAGATCCAAAGTTTTTTTCACCACATATCAATGTGGTCCATCCTAATTAAGTCCATTGTCTACCCCATTTTTGGTGGGGTCCCCAATCTATGAAAAA-TCGAATTCATTTTACTGCTCACTCACCAAAAATGTTCAACAGCCCCACGGTACATTATACAAGTAATAGTAATACAACAACATACATTAAACTTATATAG

>PI175231

AAATTCAATTATCATCTTCAGGTAAATCGGTAATTAACAAATTTAAATTTCAAATATGTTTTGATTCCTTGTATTTAG-----------GTTTGTCCTAACTCCTAACCCTAACAAACTTCCCTAAATTTTATGTGCCAAGTAAATTTATAAAA-AAAAA--CATAAAGACATGATGCGG-TGGACTCACAAACTGAAGCATAC---------AAGTTATAAAGTTTTTGAAAGCGAAATAACTTCTTAAATTTTTTTAAATATTATAATCGAATATTTCTACGACTAACAACTGAACTGAATTAACGGAATAATTAATCTATCATATATTGATTTAAGTGAATTGGACCACATCAGGGTACATAGAGTTTGGGGGGCTAGCTAGTGATTAGATCCAAAGTTTTTCTCACCACATATCAATGTGGTCCATCCTAATTAAGTCCATTGTCTACCCCATTTTTGGTGGGGTCCCCAATCTATGAAAAA-TCGAATTCATTTTACTGCTCACTCACCAAAAATGTTCAACAGCCCCACGGTACATTATACAAGTAATAGTAATACAACAACATACATTAAACTTATATAG

>PI200755

AAGTTCAATTATCATCTTCAGATAAATCGGTAATTAACAAATTTAAATTTCAAATATGTTTTGAATCCTTGTATTTAG-----------GTTTGTCCTAACTCCTAGCCCTAACGAACTTCACTAAATTTTATGTGCCAAATAAATTTATAAAA-AAAAA--CATAAAGACATGATGCGG-TGGACTCACAAACTGAAGCATAC---------AAGTTATAAAGTTTTTGAAAGCGAAATAACGTCTTAAATTTTTTTAAATATTATAATCGAATATTTCTACGACTAACAACTGAACTGAATTAACGGAATAATTAATCTATCATATATTGATTTAAGCGAATTGGACCACATCAGGGTACATAGAGTTTGGGGGGCTAGCTAGTGATTAGATCCAAAGTTTTTTTCACCACATATCAATGTGGTCCATCCTAATTAAGTCCATTGTCTACCCCATTTTTGGTGGGGTCCCCAATCTATGAAAAA-TCGAATTCATTTTACTGCTCACTCACCAAAAATGTTCAACAGCCCCACGGTACATTATACAAGTAATAGTAATACAACAACATACATTAAACTTATATAG

>PI244175

AAGTTCAATTATCATCTTCAGGTAAATCTTTAATTAACAAATTTAAATATCAAATATGTTTTGAATCCTTGTATTTAG-----------GTTTGTCCTAACTCCTAACCCTAACGAACTTCCCTAAATTTTATGTGCCAAATAAATTTATAAAA-AAAAA--CATAAAGACATGATACGG-TAGACTCACAAACTGAAGCATAC---------AAATTATAAAGTTTTTGAAAGCGAAATAACCTCTTAAATTTTTTTAAATATTATAATCGGATATTTCTACGACTAACAACTGAACTGAATTAACGGAATAATTAATCAATGATATATTGATTTAAGCGAATTGGACCACATCAGGGTACATAGAGTTTGGGGGGCTAGCTAGTGATTAGATCCAAAGTTTTTTTCACCACATATCAATGTGGTCCATCCTAATTAAGTCCATTGTCTACCCCATTTTTGGTGGGGTCCCCAATCTATGAAAAA-TCGAATTCATTTTACTGCTCACTCACCAAAAATGTTCAACAGCCCCACGGTACATTATACAAGTAATAGTAATACAACAACATACATTAAACTTATATAG

>PI180699

AAGTTCAATTATCATCTTCAGATAAATCGGTAATTAACAAATTTAAATTTCAAATATGTTTTGAATCCTTGTATTTAG-----------GTTTGTCCTAACTCCTAGCCCTAACGAACTTCACTAAATTTTATGTGCCAAATAAATTTATAAAA-AAAAA--CATAAAGACATGATGCGG-TGGACTCACAAACTGAAGCATAC---------AAGTTATAAAGTTTTTGAAAGCGAAATAACGTCTTAAATTTTTTTAAATATTATAATCGAATATTTCTACGACTAACAACTGAACTGAATTAACGGAATAATTAATCTATCATATATTGATTTAAGCGAATTGGACCACATCAGGGTACATAGAGTTTGGGGGGCTAGCTAGTGATTAGATCCAAAGTTTTTTTCACCACATATCAATGTGGTCCATCCTAATTAAGTCCATTGTCTACCCCATTTTTGGTGGGGTCCCCAATCTATGAAAAA-TCGAATTCATTTTACTGCTCACTCACCAAAAATGTTCAACAGCCCCACGGTACATTATACAAGTAATAGTAATACAACAACATACATTAAACTTATATAG

>PI206006

AAGTTCAATTATCATCTTCAGATAAATCGGTAATTAACAAATTTAAATTTCAAATATGTTTTGAATCCTTGTATTTAG-----------GTTTGTCCTAACTCCTAGCCCTAACGAACTTCACTAAATTTTATGTGCCAAATAAATTTATAAAA-AAAAA--CATAAAGACATGATGCGG-TGGACTCACAAACTGAAGCATAC---------AAGTTATAAAGTTTTTGAAAGCGAAATAACGTCTTAAATTTTTTTAAATATTATAATCGAATATTTCTACGACTAACAACTGAACTGAATTAACGGAATAATTAATCTATCATATATTGATTTAAGCGAATTGGACCACATCAGGGTACATAGAGTTTGGGGGGCTAGCTAGTGATTAGATCCAAAGTTTTTTTCACCACATATCAATGTGGTCCATCCTAATTAAGTCCATTGTCTACCCCATTTTTGGTGGGGTCCCCAATCTATGAAAAA-TCGAATTCATTTTACTGCTCACTCACCAAAAATGTTCAACAGCCCCACGGTACATTATACAAGTAATAGTAATACAACAACATACATTAAACTTATATAG

>PI250447

AAGTTCAATTATCATCTTCAGATAAATCGGTAATTAACAAATTTAAATTTCAAATATGTTTTGAATCCTTGTATTTAG-----------GTTTGTCCTAACTCCTAGCCCTAACGAACTTCACTAAATTTTATGTGCCAAATAAATTTATAAAA-AAAAA--CATAAAGACATGATGCGG-TGGACTCACAAACTGAAGCATAC---------AAGTTATAAAGTTTTTGAAAGCGAAATAACGTCTTAAATTTTTTTAAATATTATAATCGAATATTTCTACGACTAACAACTGAACTGAATTAACGGAATAATTAATCTATCATATATTGATTTAAGCGAATTGGACCACATCAGGGTACATAGAGTTTGGGGGGCTAGCTAGTGATTAGATCCAAAGTTTTTTTCACCACATATCAATGTGGTCCATCCTAATTAAGTCCATTGTCTACCCCATTTTTGGTGGGGTCCCCAATCTATGAAAAA-TCGAATTCATTTTACTGCTCACTCACCAAAAATGTTCAACAGCCCCACGGTACATTATACAAGTAATAGTAATACAACAACATACATTAAACTTATATAG

>PI261623

AAGTTCAATTATCATCTTCAGGTAAATCTTTAATTAACAAATTTAAATATCAAATATGTTTTGAATCCTTGTATTTAG-----------GTTTGTCCTAACTCCTAACCCTAACGAACTTCCCTAAATTTTATGTGCCAAATAAATTTATAAAA-AAAAA--CATAAAGACATGATACGG-TAGACTCACAAACTGAAGCATAC---------AAATTATAAAGTTTTTGAAAGCGAAATAACCTCTTAAATTTTTTTAAATATTATAATCGGATATTTCTACGACTAACAACTGAACTGAATTAACGGAATAATTAATCAATGATATATTGATTTAAGCGAATTGGACCACATCAGGGTACATAGAGTTTGGGGGGCTAGCTAGTGATTAGATCCAAAGTTTTTTTCACCACATATCAATGTGGTCCATCCTAATTAAGTCCATTGTCTACCCCATTTTTGGTGGGGTCCCCAATCTATGAAAAA-TCGAATTCATTTTACTGCTCACTCACCAAAAATGTTCAACAGCCCCACGGTACATTATACAAGTAATAGTAATACAACAACATACATTAAACTTATATAG

>PI261671

AAGTTCAATTATCATCTTCAGATAAATCGGTAATTAACAAATTTAAATTTCAAATATGTTTTGAATCCTTGTATTTAG-----------GTTTGTCCTAACTCCTAGCCCTAACGAACTTCACTAAATTTTATGTGCCAAATAAATTTATAAAA-AAAAA--CATAAAGACATGATGCGG-TGGACTCACAAACTGAAGCATAC---------AAGTTATAAAGTTTTTGAAAGCGAAATAACGTCTTAAATTTTTTTAAATATTATAATCGAATATTTCTACGACTAACAACTGAACTGAATTAACGGAATAATTAATCTATCATATATTGATTTAAGCGAATTGGACCACATCAGGGTACATAGAGTTTGGGGGGCTAGCTAGTGATTAGATCCAAAGTTTTTTTCACCACATATCAATGTGGTCCATCCTAATTAAGTCCATTGTCTACCCCATTTTTGGTGGGGTCCCCAATCTATGAAAAA-TCGAATTCATTTTACTGCTCACTCACCAAAAATGTTCAACAGCCCCACGGTACATTATACAAGTAATAGTAATACAACAACATACATTAAACTTATATAG

>PI261677

AAGTTCAATTATCATCTTCAGGTAAATCTTTAATTAACAAATTTAAATATCAAATATGTTTTGAATCCTTGTATTTAG-----------GTTTGTCCTAACTCCTAACCCTAACGAACTTCCCTAAATTTTATGTGCCAAATAAATTTATAAAA-AAAAAA-CATAAAGACATGATACGG-TAGACTCACAAACTGAAGCATAC---------AAATTATAAAGTTTTTGAAAGCGAAATAACTTCTTAAATTTTTTTAAATATTATAATCGGATATTTCTACGACTAACAACTGAACTGAATTAACGGAATAATTAATCAATCATATATTGATTTAAGCGAATTGGACCACATCAGGGTACATAGAGTTTGGGGGGCTAGCTAGTGATTAGATCCAAAGTTTTTTTCACCACATATCAATGTGGTCCATCCTAATTAAGTCCATTGTCTACCCCATTTTTGGTGGGGTCCCCAATCTATGAAAAA-TCGAATTCATTTTACTGCTCACTCACCAAAAATGTTCAACAGCCCCACGGTACATTATACAAGTAATAGTAATACAACAACATACATTAAACTTATATAG

>PI263014

AAGTTCAATTATCATCTTCAGGTAAATCTTTAATTAACAAATTTAAATATCAAATATGTTTTGAATCCTTGTATTTAG-----------GTTTGTCCTAACTCCTAACCCTAACGAACTTCCCTAAATTTTATGTGCCAAATAAATTTATAAAA-AAAAA--CATAAAGACATGATACGG-TAGACTCACAAACTGAAGCATAC---------AAATTATAAAGTTTTTGAAAGCGAAATAACTTCTTAAATTTTTTTAAATATTATAATCGGATATTTCTACTACTAACAACTGAACTGAATTAACGGAATAATTAATCAATGATATATTGATTTAAGCGAATTGGACCACATCAGGGTACATAGAGTTTGGGGGGCTAGCTAGTGATTAGATCCAAAGTTTTTTTCACCACATATCAATGTGGTCCATCCTAATTAAGTCCATTGTCTACCCCATTTTTGGTGGGGTCCCCAATCTATGAAAAA-TCGAATTCATTTTACTGCTCACTCACCAAAAATGTTCAACAGCCCCACGGTACATTATACAAGTAATAGTAATACAACAACATACATTAAACTTATATAG

>PI273605

AAGTTCAATTATCATCTTCAGGTAAATCGGTAATTAACAAATTTAAATTTCAAATATGTTTTGAATCCTTGTATTTAG-----------GTTTGTCCTAACTCCTAGCCCTAACGAACTTCACTAAATTTTATGTGCCAAATAAATTTATAAAA-AAAAA--CATAAAGACATGATGCGG-TGGACTCACAAACTGAAGCATAC---------AAGTTATAAAGTTTTTGAAAGCGAAATAACGTCTTAAATTTTTTTAAATATTATAATCGAATATTTCTACGACTAACAACTGAACTGAATTAACGGAATAATTAATCTATCATATATTGATTTAAGCGAATTGGACCACATCAGGGTACATAGAGTTTGGGGGGCTAGCTAGTGATTAGATCCAAAGTTTTTCTCACCACATATCAATGTGGTCCATCCTAATTAAGTCCATTGTCTACCCCATTTTTGGTGGGGTCCCCAATCTATGAAAAA-TCGAATTCATTTTACTGCTCACTCACCAAAAATGTTCAACAGCCCCACGGTACATTATACAAGTAATAGTAATACAACAACATACATTAAACTTATATAG

>PI314794

AAGTTCAATTATCATCTTCAGATAAATCGGTAATTAACAAATTTAAATTTCAAATATGTTTTGAATCCTTGTATTTAG-----------GTTTGTCCTAACTCCTAGCCCTAACGAACTTCACTAAATTTTATGTGCCAAATAAATTTATAAAA-AAAAA--CATAAAGACATGATGCGG-TGGACTCACAAACTGAAGCATAC---------AAGTTATAAAGTTTTTGAAAGCGAAATAACGTCTTAAATTTTTTTAAATATTATAATCGAATATTTCTACGACTAACAACTGAACTGAATTAACGGAATAATTAATCTATCATATATTGATTTAAGCGAATTGGACCACATCAGGGTACATAGAGTTTGGGGGGCTAGCTAGTGATTAGATCCAAAGTTTTTTTCACCACATATCAATGTGGTCCATCCTAATTAAGTCCATTGTCTACCCCATTTTTGGTGGGGTCCCCAATCTATGAAAAA-TCGAATTCATTTTACTGCTCACTCACCAAAAATGTTCAACAGCCCCACGGTACATTATACAAGTAATAGTAATACAACAACATACATTAAACTTATATAG

>PI324700

AAGTTCAATTATCATCTTCAGATAAATCGGTAATTAACAAATTTAAATTTCAAATATGTTTTGAATCCTTGTATTTAG-----------GTTTGTCCTAACTCCTAGCCCTAACGAACTTCACTAAATTTTATGTGCCAAATAAATTTATAAAA-AAAAA--CATAAAGACATGATGCGG-TGGACTCACAAACTGAAGCATAC---------AAGTTATAAAGTTTTTGAAAGCGAAATAACGTCTTAAATTTTTTTAAATATTATAATCGAATATTTCTACGACTAACAACTGAACTGAATTAACGGAATAATTAATCTATCATATATTGATTTAAGCGAATTGGACCACATCAGGGTACATAGAGTTTGGGGGGCTAGCTAGTGATTAGATCCAAAGTTTTTTTCACCACATATCAATGTGGTCCATCCTAATTAAGTCCATTGTCTACCCCATTTTTGGTGGGGTCCCCAATCTATGAAAAA-TCGAATTCATTTTACTGCTCACTCACCAAAAATGTTCAACAGCCCCACGGTACATTATACAAGTAATAGTAATACAACAACATACATTAAACTTATATAG

>PI343979

AAGTTCAATTATCATCTTCAGGTAAATCGGTAATTAACAAATTTAAATTTCAAATATGTTTTGAATCCTTGTATTTAG-----------GTTTGTCCTAACTCCTAACCCTAACGAACTTCCCTAAATTTTATGTGCCAAATAAATTTATAAAA-AAAAAA-CATAAAGACATGATGCGG-TGCACTCACAAACTAAAGCATAC---------GAGTTATAAAGTTTTTGAAAGCGAAATAACTTCTTAAATTTTTTTAAATATTATAATCGGATATTTCTACTATTAACAACTGAACTGAATTAACGGAACAATTAATCAATCATATATTGATTTAAGCGAATTGGACCACATCAGGGTACATAGAGTTTGGGGGGCTAGCTAGTGATTAGATCCAAAGTTTTTCTCACCACATATCAATGTGGTCCATCCTAATTAAGTCCATTGTCTACCCCATTTTTGGTGGGGTCCCCAATCTATGAAAAA-TCGAATTCATTTTACTGCTCACTCACCAAAAATGTTCAACAGCCCCACGGTACATTATACAAGTAATAGTAATACAACAACATACATTAAACTTATATAG

>PI344012

AAGTTCAATTATCATCTTCAGGTAAATCGGTAATTAACAAATTTAAATTTCAAATATATTTTGATTCCTTGTATTTAG-----------ATTTGTCCTAACTCCTAACCCTAACGAGCTTCCCTAAATTTTATGTGTCAAACAAATTTATAAAA-AAAAAAACATAAACACATGATACAG-TGGACTCACAAACTAAAGCATAC---------AAGTTATAAAGTTTTTGAAAGCGAAATAACTTCTTAAAAAATTTTAAATATTATAATCGGATATTTCTACGGCAAACAACTGAACTGAATTAACGGAACAATTAATCAATCATATATTGATTTAAGCGAATTGGACCACATCAGG-TACATAGAGTTTGGGGGGCTAGCTAGTGATTAGATCCAAACTCTTTTTCACCACATATCAATGTGGTCCATCCTAATTAAGTCCATTGTCTACCCCATTTTTGGTGGGGTCCCCAATCTATGAAAAA-TCGAATTCATTTTACTGCTCAGTCACCAAAAATGTTCAACAGCCCCACGGTACATTATACAAGTAATAGTAATACAACAACATACATTAAACTTATATAG

>PI344538

AAGTTCAATTATCATCTTCAGGTAAATCGGTAATTAACAAATTTAAATTTCAAATATATTTTGATTCCTTGTATTTAG-----------GTTTGTCCTAACTCCTAACCCTAACTAGCTTCCCTAAATTTTATGTGCCAATCAAATTTATAAAA-AAAAAAACATAAACACATGATACAG-TGGACTCACAAACTAAAGCATAG---------AAGTTATAAAGTTTTTGAAAGCGAAATAACTTCTTAAATTTTTTTAAATATTATAATCGGATATTTCTACGGCTAACAACTGAACTGAATTAACGGAACAATTAATCAATGATATATTGATTTAAGCGAATTGGACCACATCAGG-TACATAGAGTTTGGGGGGCTAGCTAGTGATTAGATCCAAACTCTTTTTCACCACATATCAATGTGGTCCATCCTAATTAAGTCCATTGTCTACCCCATTTTTGGTGGGGTCCCCAATCTATGAAAAA-TCGAATTCATTTTACTGCTCAGTCACCAAAAATGTTCAACAGCCCCACGGTACATTATACAAGTAATAGTAATACAACAACATACATTAAACTTATATAG

>PI358613

AAGTTCAATTATCATCTTCAGGTAAATCGGTAATTAACAAATTTAAATTTCAAATATATTTTGATTCCTTGTATTTAG-----------GTTTGTCCTAACTCCTAACCCTAACGAGCTTCCCTAAATTTTATGTGCCAAACAAATTTATAAAA-AAAAAA-CATAAACACATGATACAG-TGGACTTACAAACTAAAGCATAA---------AAGTTATAAAGTTTTTGAAAGCGAAATAACTTCTTAAAATTTTTTAAATATTATAATCGGATATTTCTACGGCTAACAACTGAACTGAATTAACGGAACAATTAATCAATCATATATTGATTTAAGCGAATTGGACCACATCAGG-TACATAGAGTTTGGGGGGCTAGCTAGTGATTAGATCCAAACTCTTTTTCACCACATATCAATGTGGTCCATCCTAATTAAGTCCATTGTCTACCCCATTTTTGGTGGGGTCCCCAATCTATGAAAAA-TCGAATTCTTTTTACTGCTCAGTCACCAAAAATGTTCAACAGCCCCACGGTACATTATACAAGTAATAGTAATACAACAACATACATTAAACTTATATAG

>PI429839

AAGTTCAATTATCATCTTCAGGTAAATCTTTAATTAACAAATTTAAATATCAAATATGTTTTGAATCCTTGTATTTAG-----------GTTTGTCCTAACTCCTAACCCTAACGAACTTCCCTAAATTTTATGTGCCAAATAAATTTATAAAA-AAAAA--CATAAAGACATGATACGG-TAGACTCACAAACTGAAGCATAC---------AAATTATAAAGTTTTTGAAAGCGAAATAACTTCTTAAATTTTTTTAAATATTATAATCGGATATTTCTACGACTAACAACTGAACTGAATTAACGGAATAATTAATCTATCATATATTGATTTAAGCGAATTGGACCACAT

>PI429843

AAGTTCAATTATCATCTTCAGGTAAATCTTTAATTAACAAATTTAAATATCAAATATGTTTTGAATCCTTGTATTTAG-----------GTTTGTCCTAACTCATAACCCTAACGAACTTCCCTAAATTTTATGTGCCAAATAAATTTATAAAA-AAAAA--CATAAAGACATGATACGG-TAGACTCACAAACTAAAGCATAC---------AAATTATAAAGTTTTTGAAAGCGAAATAACTTCTTAAATTTTTTTAAATATTATAATCGGATATTTCTGCGACTAACAACTGAACTGAATTAACGGAATAATTAATCAATCATATATTGATTTAAGCGAATTGGACCACATCAGGGTACATAGAGTTTGGGGGGCTAGCTAGTGATTAGATCCAAAGTTTTTTTCACCACATATCAATGTGGTCCATCCTAATTAAGTCCATTGTCTACCCCATTTTTGGTGGGGTCCCCAATCTATGAAAAA-TCGAATTCATTTTACTGCTCACTCACCAAAAATGTTCAACAGCCCCACGGTACATTATACAAGTAATAGTAATACAACAACATACATTAAACTTATATAG
