# Supplementary material for: Identification of Mendel's White Flower Character
Source: PLoS One. 2010 Oct 11;5(10):e13230. doi: 10.1371/journal.pone.0013230 (PMC2952588; doi:10.1371/journal.pone.0013230)
Supplement: Figure S4 — Annotated sequence of the A2 gene from JI2822 and showing differences in various lines. The sequence from nt 160 to nt 217 (bold italic) is a direct duplication, only one copy of which is found in the PI 198074 allele. From nucleotide 530 to 644 (bold italic) is also deleted in the PI 198074 allele. Note the GCACA sequence is repeated at either end of this deleted segment. The G to A SNP at nucleotide 1207 in JI2673 that changes TGG (W) to TGA (stop) is indicated in bold, cv Melrose in which this mutation arose has the sequence TGG as expected. The deletions from nucleotide 1360 to 1381 in FN3171 and from nucleotide 1510 to 1514 in JI 3062 are highlighted in bold italics. The line JI 3061, which carries the progenitor allele of this mutation, has the same sequence as JI 2822 at that point. The encoded amino acid sequence and other features of note are indicated. Four SNPs identified in the lines PI 193578 PI 198074 and PI 266070 are indicated in bold and ‘in PIs’ (all carry the same SNP with respect to JI2822). (0.04 MB DOC) [file pone.0013230.s004.doc]

**Figure S4** Annotated sequence of the *A2* gene from JI2822 and showing differences in various lines.

The sequence from nt 160 to nt 217 (bold italic) is a direct duplication, only one copy of which is found in the PI 198074 allele. From nucleotide 530 to 644 (bold italic) is also deleted in the PI 198074 allele. Note the GCACA sequence is repeated at either end of this deleted segment. The G to A SNP at nucleotide 1207 in JI2673 that changes TGG (W) to TGA (stop) is indicated in bold, cv Melrose in which this mutation arose has the sequence TGG as expected. The deletions from nucleotide 1360 to 1381 in FN3171 and from nucleotide 1510 to 1514 in JI 3062 are highlighted in bold italics. The line JI 3061, which carries the progenitor allele of this mutation, has the same sequence as JI 2822 at that point. The encoded amino acid sequence and other features of note are indicated. Four SNPs identified in the lines PI 193578 PI 198074 and PI 266070 are indicated in bold and ‘in PIs’ (all carry the same SNP with respect to JI2822).

1 CTCCCAAGGACCGTCTTAATACTCCATAAATTCTTAACAATATGGTCAACCAAAAGGAGA

61 ATCATCAGTATTACCGAAGATGATAAACCACTATGTCGGTCGAACCCACCCTAAAATATG

121 GGTAAAGACAAAACTCCACCTTCCTATAAACTAATCCAC

160 ***TAAGCCCTTAAAGCACAAACAAGAAAAAA***

189 ***TAAGCCCTTAAAGCACAAACAAGAAAAAA*** **PI 198074 deletion**

218 AAAGAGTTAGGAGATCCCCATGT

241 TTCAAACCCCTTTAAATACTAATCTCTTGAGTTAGACACCCATTAACTAACATTGCAAAA

301 TTTTCATTAAGCAGACAAACTCTAATCCAAAATATTCACTTCTCATTTAAACCAAAATCT

**potential TATA box**

361 AATATA**CATATATAT**GTATCACTCTTTCCTCTCTTTTAAAAGTTCTCCCTATCTCCTTTT

421 GAGCCAATGTCTTCTAAAAGTTCTTTACGAGCATGCACTAATATGTCTTTTTTTTTCATT

481 CTTAGGAGTCTTGTATGGAACCAGTCCAAGTCCTTTCTTTCTTCGAGCA***CACATCTATCG***

**-----------**

541 ***ATCTCTCTAGCTTTCTCTATTTGCAAACACAAACATTATATTAAAACAAGGCATAGGAAA***

**------------------------------------------------------------**

601 ***TAATGRGACAAAATTGGTGGTTATGGTTTAATAATGGGTTGTTG***CACATCTTAGTGAGTT

**--------------------------------------------PI 198074 deletion**

661 CACAATTGCGAACGTGAAGGAACATTCCCGTCAATTTCGACTTACCACAAAACAAGAGCT

721 AAGTGAAGAAGACGCCGAAGAAGGAAGTTGAAAATTTAAGGCGCGTGCGATTCATTGTTT

781 CTTTGACCCCTTCTCTTCAATTTTATAGTTCCCACCTAACTTTTCTTTTTCTATCTTCTT

**M** D N S T Q E S H L

841 CTTCACCCCTCCTCTTCTTCCCACACCAAAAATGGATAATTCCACTCAAGAATCCCATCT

R S D N N S V T Y D S P H P L Y A M A F

901 CCGATCCGATAACAACTCCGTCACCTATGATTCTCCTCATCCTCTTTACGCCATGGCTTT

S S N P N P Q H H Q R I A V G S F I E E

961 CTCTTCAAACCCCAATCCCCAACACCACCAACGAATCGCCGTCGGTAGCTTCATCGAAGA

Y T N R V D I L S F N P D T L S I K P Q

1021 ATACACTAACCGCGTCGATATCCTCTCTTTCAACCCCGATACTCTCTCCATTAAACCTCA

P S L S F E S P Y P P T K L M F H P A T

1081 ACCTTCACTTTCATTCGAATCCCCTTACCCACCTACCAAACTCATGTTCCACCCCGCAAC

H S S L Q K T S S D L L A T S G D Y L R

1141 CCATTCCTCCCTCCAGAAAACATCCTCCGACCTCCTCGCCACCTCCGGCGACTATCTCCG

L W E V R E N S V E A L S L F N N S K T

1201 TCTCTG**G**GAAGTTCGTGAGAATTCCGTCGAGGCTCTTTCTCTCTTCAACAACAGCAAAAC

**A** **SNP in JI 2673**

S E F C A P L T S F D W N E I E P K R I

1261 CAGTGAGTTTTGTGCTCCGTTAACCTCCTTCGACTGGAACGAAATTGAGCCGAAACGCAT

**< WD40 domain start**

G T S S I D T T C T I W D I E R G V V E

1321 CGGAACTTCCAGTATCGACACTACTTGTACTATCTGGGA***CATTGAAAGAGGGGTTGTTGA***

**---------------------**

T Q L I A H D K E V Y D I A W G E S R V

1381 ***A***ACTCAGCTTATTGCACACGATAAAGAGGTTTACGATATCGCTTGGGGGGAATCCAGAGT

**-** **FN 3171 deletion**

F A S V S A D G S V R I F D L R D K E H

1441 TTTCGCTTCTGTTTCTGCTGATGGGTCTGTTAGGATCTTTGATCTAAGAGACAAAGAGCA

S T I I Y E S P Q P D T P L L R L A W N

1501 TTCTACAAT***TATCT***ATGAGAGTCCTCAACCAGATACTCCTTTGCTTCGTTTGGCTTGGAA

**----- JI 3062 deletion**

K K D L R Y M A T I L M D S N K V V I L

1561 CAAGAAGGATTTGAGATACATGGCAACTATTTTGATGGATAGTAATAAAGTTGTCATTTT

D I R S P T T P V A E L E R H R A G V N

1621 GGATATTCGATCACCAACCACGCCTGTCGCGGAATTGGAGAGGCATCGTGCTGGTGTTAA

A I A W A P R S S K H I C S A G D D T Q

1681 TGCGATTGCTTGGGCTCCAAGAAGCTCTAAGCATATTTGTTCTGCTGGAGATGATACGCA

**WD40 domain end >**

A L M W E L P T V A G P N G I D P M S M

1741 GGCTCTTATGTGGGAGTTACCTACGGTGGCTGGTCCAAATGGAATTGATCCAATGTCTAT

Y S S G Y E I N Q L Q W S A A Q P D W I

1801 GTATTCTTCTGGTTATGAGATTAATCAGCTTCAGTGGTCTGCTGCTCAGCCGGATTGGAT

A I A F A N K M Q L L R V *****

1861 TGCAATTGCTTTTGCAAACAAGATGCAGCTTTTGAGGGTTTGAATTTCAGGTCAGGGAAT

1921 AGTTTACTTGTAGTTTTGGAAAACCAAATTACTCATTGCAGTGTTGTGAGTTGTGACTGA

1981 TATGTATTGAGTAGTCTAGTTTAACTTGGTCATGCATTAATTAGAGACGACTCGATGATT

2041 CTTATGCTTGCCTTATACTTTAGTACTGTCTAATGTATGCAATGGCTTCTTTTGGTGGCC

2101 TTAAACTACCACTGTCTC**T**CATATGTAAGAGTTATAGTCGGGGCATACTAGTTTACCTTC

**C in PIs**

2161 ATGTTTTACTTATAGCTAAAATTGATGTTGCATTTGCTTGTTGAATTGAAGATGGAATTT

2221 ACCAGTTTAATATTGTAGTTGCTGCTCTTCTAGATGATCCTTGTTAAATTTCGAAGTTGA

2281 TTCTTTTTTGTATTGTTATTGAGCACCTTTGTATGTATGTCTTAGTAATAATTTTAATCA

2341 CAGTTCGACACTTCATTAGTTTTTGAGAAAGATGGTTTATATGTCGGAGTCGTGACAATG

2401 TAATGAATGCTTGTTTATTAAACTTTGCTAAAGCCTTCATTCATGCCATTTATGTAGATA

2461 TTTAGTCAATGAAGTATACTTGTATTCCTCGGTTATAAGAGCTGCAGTGTCGCATTGGAT

2521 TGGATTGATTATTTTTTCAGTAAAAAATAAGAAGTATACTTGGTACTGCAGAAATGAGAA

2581 TATTACAATG**T**AATAGTAATTACAGATGGTTATGTAAGATTTTG**T**AAAAGAGAAAATTGT

**C in PIs A in PIs**

2641 GTAGCCCTCAGTAAAGAAAACACAGTATAGTATTGTTTTTGGTGATTTGAGTATGTGTGG

2701 ATAAGTTTTGTAGAGCTAAGAAATGTTGACTGGATGGTGGAT**A**GTTCGGTAGTTAGAGTT

**G in PIs**

2761 GAAAGAAGGCTGAGGAAAGTTATATGCCAATAAGAGAGATTTATGACAGGATTTTTTCAA

2821 GCATTAATGGTATGGTCCTACATGCCACGGCTGCCAGAACATACTACGAATAGAGCAAGA

2881 TCCTTT
