# Supplementary material for: Identification of Mendel's White Flower Character
Source: PLoS One. 2010 Oct 11;5(10):e13230. doi: 10.1371/journal.pone.0013230 (PMC2952588; doi:10.1371/journal.pone.0013230)
Supplement: Figure S5 — Complementation of white pea petals by particle bombardment. Particle bombardment of petals of Greenfeast (PI 250447 that carries the G to A splice donor mutation) with [left to right], over-expression cassettes for PhAN1 (AN1), reproduced from Figure 3 for comparison, and PhAN2. All experiments included an over-expression cassette of green fluorescent protein (GFP) co-precipitated onto the gold particle prior to bombardment. Anthocyanin accumulation is shown (upper panels). Fluorescence due to expression of the GFP protein (lower panels) was recorded two days after bombardment. (4.74 MB DOC) [file pone.0013230.s005.doc]

**Figure S5.** Complementation of white pea petals by particle bombardment. Particle bombardment of petals of Greenfeast (PI 250447 that carries the G to A splice donor mutation) with [left to right], over-expression cassettes for PhAN1 (AN1), reproduced from Fig3 for comparison, and PhAN2. All experiments included an over-expression cassette of green fluorescent protein (GFP) co-precipitated onto the gold particle prior to bombardment. Anthocyanin accumulation is shown (*upper panels*). Fluorescence due to expression of the GFP protein (*lower panels*) was recorded two days after bombardment.

**
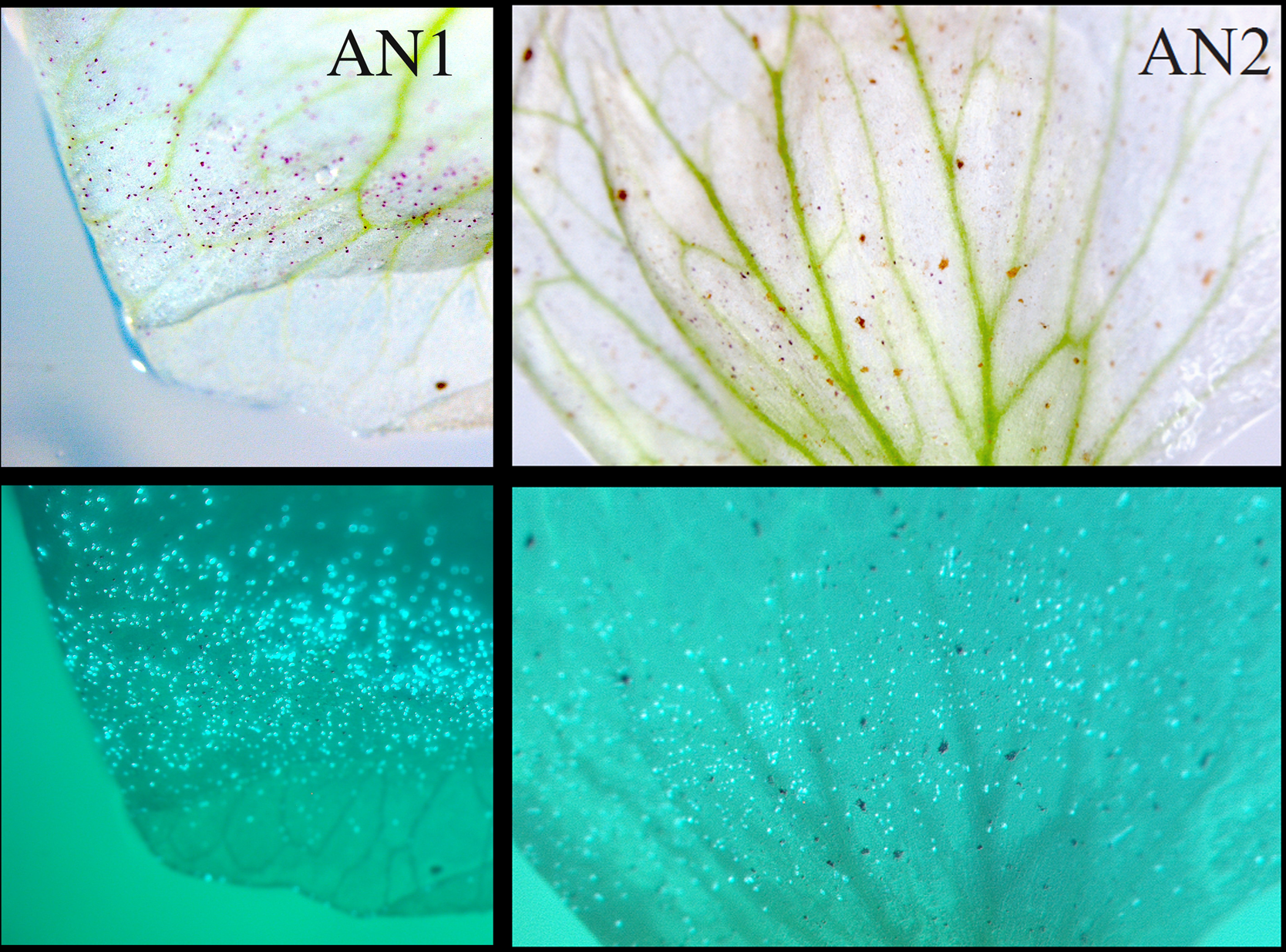
**
