# Supplementary material for: Identification of Mendel's White Flower Character
Source: PLoS One. 2010 Oct 11;5(10):e13230. doi: 10.1371/journal.pone.0013230 (PMC2952588; doi:10.1371/journal.pone.0013230)
Supplement: Figure S6 — Alignment of the predicted amino acid sequence of the N-terminal region of bHLH proteins. (0.04 MB DOC) [file pone.0013230.s006.doc]

**Fig. S6** Alignment of the predicted amino acid sequence of the N-terminal region of bHLH proteins.

PileUp

MSF: 207 Type: P Check: 9649 ..

Name: A Len: 207 Check: 5778 Weight: 0

Name: MtbHLH Len: 207 Check: 6211 Weight: 0

Name: PhAN1 Len: 207 Check: 3742 Weight: 0

Name: AtTT8 Len: 207 Check: 1114 Weight: 0

Name: AtMYC2 Len: 207 Check: 9357 Weight: 0

Name: AtMYC1 Len: 207 Check: 4020 Weight: 0

Name: AtEGL1 Len: 207 Check: 8371 Weight: 0

Name: AtGL3 Len: 207 Check: 655 Weight: 0

Name: AmDEL Len: 207 Check: 3669 Weight: 0

Name: PhJAF13 Len: 207 Check: 6732 Weight: 0

//

1 50

A GCNKLQNMLQ AAVQSVQWTY SLFWQICPQQ ...LILVWGD GYYNGAIKTR

MtbHLH ...KLQNMLQ AAVQSVQWTY SLFWQLCPQQ ...LILVWGD GYYNGSIKTR

PhAN1 ...QLQTMLR NAVQSVQWTY SLFWQLCPQQ ...GVLVWRD GYYNGAIKTR

AtTT8 EKKELQGLLK TAVQSVDWTY SVFWQFCPQQ ...RVLVWGN GYYNGAIKTR

AtMYC2 .TLQQRLQAL IEGTHEGWTY AIFWQPSYDF SGASVLGWGD GYYKGEEDKA

AtMYC1 QNSLLRKQLA LAVRSVQWSY AIFWSSSLTQ P..GVLEWGE GCYNGDMKKR

AtEGL1 VPDNLKKQLA VSVRNIQWSY GIFWSVSASQ P..GVLEWGD GYYNGDIKTR

AtGL3 VPENLKKHLA VSVRNIQWSY GIFWSVSASQ S..GVLEWGD GYYNGDIKTR

AmDEL VPENLRKQLA IAVRSIQWSY AIFWSNSVAQ P..GVLEWGD GFYNGDIKTR

PhJAF13 VPDNLREQLA FAVRGIQWSY AILWSTTVSQ P..GELEWSD SNYNGDIKTR

51 100

A KTVQPMEVSA EEASLQRSQQ LRELYESLSA GETN...... ........PP

MtbHLH KTVQPMEVSA EEASLQRSQQ LRELYESLSA GETN...... ........PP

PhAN1 KTVQPMEVSA EEASLHRSQQ LRELYESLSA GESN...... ........QP

AtTT8 KTTQPAEVTA EEAALERSQQ LRELYETLLA GEST...... ........SE

AtMYC2 NPRRRSSSPP FSTPADQEYR KKVLRELNSL ISGG...... ..........

AtMYC1 K...KSYESH YKYGLQKSKE LRKLYLSMLE GDSGTTVSTT HDNLNDDDDN

AtEGL1 KTIQAAEVKI DQLGLERSEQ LRELYESLSL AESSAS.... ....GSSQVT

AtGL3 KTIQASEIKA DQLGLRRSEQ LSELYESLSV AESSSSG.VA ....AGSQVT

AmDEL KTVQSVELNQ DQLGLQRSDQ LRELYESLSL GETN...... ........TQ

PhJAF13 KTVQAGEVDE DQLGLQRTEQ LRDLYSSLLI GEGEEDL... .......QPQ

101 150

A TRRPCASLSP EDLTESEWFY LMCVSFSFPP GVGLPGKAYA RRQHVWLTGA

MtbHLH TRRPCASLSP EDLTESEWFY LMCVSFSFPP GVGLPGRAYT KRQHIWLTGA

PhAN1 TRRPSAALSP EDLTESEWFY LMCVSFSFPA GIGLPGKAYS KKHHIWITGA

AtTT8 AR.ACTALSP EDLTETEWFY LMCVSFSFPP PSGMPGKAYA RRKHVWLSGA

AtMYC2 .VAPSDDAVD EEVTDTEWFF LVSMTQSFAC GAGLAGKAFA TGNAVWVSGS

AtMYC1 CHSTSMMLSP DDLSDEEWYY LVSMSYVFSP SQCLPGRASA TGETIWLCNA

AtEGL1 RRASAAALSP EDLTDTEWYY LVCMSFVFNI GEGIPGGALS NGEPIWLCNA

AtGL3 RRASAAALSP EDLADTEWYY LVCMSFVFNI GEGMPGRTFA NGEPIWLCNA

AmDEL AKRPTAALSP EDLTDAEWFF LVCMSFIFNI GQGLPGRTLA RNQAVWLCNA

PhJAF13 AKRPSAALSP EDLTDTVWYF LVCMSFVFNV GQGLPGKSLA RHETIWLCNA

151 200

A NEVDSKTFSR AILAK..... TVVCIPVLDG VVEIGTTDKI QEDLNFIKHV

MtbHLH NEVDSKIFSR AILAK..... TVVCIPVLDG VVEFGTTDKV QEDLNFIKHV

PhAN1 NEVESKVFCR AILAKSARVQ TVVCIPLLDG VVELGTTQRI QEDIGFINHV

AtTT8 NEVDSKTFSR AILAKSAKIQ TVVCIPMLDG VVELGTTKKV REDVEFVELT

AtMYC2 DQLSGSGCER AKQGGVFGMH TIACIPSANG VVEVGSTEPI RQSSDLINKV

AtMYC1 QYAENKLFSR SLLARSASIQ TVVCFPYLGG VIELGVTELI SEDHNLLRNI

AtEGL1 ETADSKVFTR SLLAKSASLQ TVVCFPFLGG VLEIGTTEHI KEDMNVIQSV

AtGL3 HTADSKVFSR SLLAKSAAVK TVVCFPFLGG VVEIGTTEHI TEDMNVIQCV

AmDEL HRADTKVFSR SLLAKSASIQ TVVCFPYSEG VVELGATELV PEDLNLIQHI

PhJAF13 HQAESSVFSR SLIAKSASIQ TVVCFPYLGG VIELGVTELV VEDPNLIQQI

201

A RSFFID.

MtbHLH KSFFLD.

PhAN1 KTFFIE.

AtTT8 KSFFYD.

AtMYC2 RILFNFD

AtMYC1 KSCLME.

AtEGL1 KTLFLE.

AtGL3 KTSFLE.

AmDEL KTSFLD.

PhJAF13 KISILK.
