# Supplementary material for: Identification of Mendel's White Flower Character
Source: PLoS One. 2010 Oct 11;5(10):e13230. doi: 10.1371/journal.pone.0013230 (PMC2952588; doi:10.1371/journal.pone.0013230)
Supplement: Table S1 — A) Primers used in the identification and characterization of the bHLH gene from pea (Ps) [GU132941] and M. truncatula (Mt) [GU132940]. (B) Primers used in the identification and characterization of the WD40 gene from pea. (0.11 MB DOC) [file pone.0013230.s007.doc]

**Table S1**. (*A*) Primers used in the identification and characterization of the *bHLH* gene from pea (Ps) [GU132941] and *M. truncatula* (Mt) [GU132940]

| **primer name** | **orientation** | 5'--+3' **sequence** | **designed on** | **location** | **comment** |
| --- | --- | --- | --- | --- | --- |
| RPH-445: MtbHLH-A1 | Forward | ATGGCTGCTCCATCTCCATTAGGCA | MtbHLHA | exon 1 | degenerate PCR on pea DNA |
| RPH-446: MtbHLH-A2 | Reverse | CCTACACCAGGAGGAAAAGAGAAAG | MtbHLHA | exon 2 | degenerate PCR on pea DNA |
| RPH-447: MtbHLH-A3 | Forward | GATTCTAGTATGGGGTGATGGATAT | MtbHLHA | exon 2 | degenerate PCR on pea DNA |
| RPH-448: MtbHLH-A4 | Reverse | CTTAGCTAGAATAGCTCTTGAAAAT | MtbHLHA | exon 3 | degenerate PCR on pea DNA |
| RPH-449: MtbHLH-A5 | Forward | GTTGCCTGGAAGGGCATACACAAAG | MtbHLHA | exon 3 | degenerate PCR on pea DNA |
| RPH-450: MtbHLH-A6 | Reverse | CTTATCCGTTGTGCCAAACTCAACG | MtbHLHA | exon 5 | degenerate PCR on pea DNA |
| RPH-451: MtbHLH-A7 | Forward | ACTGTGGTATGTATTCCTGTATTGG | MtbHLHA | exon 5 | degenerate PCR on pea DNA |
| RPH-452: MtbHLH-A8 | Reverse | CTGAGGATGGTAGTTGAACTTGTAG | MtbHLHA | exon6 | degenerate PCR on pea DNA |
| RPH-453: MtbHLH-A9 | Forward | GTTCAAGAAGACCTTAATTTCATCA | MtbHLHA | exon6 | degenerate PCR on pea DNA |
| RPH-454: MtbHLH-A10 | Reverse | CTAAATGTTGTGAGGTATAATTTGG | MtbHLHA | exon7 | degenerate PCR on pea DNA |
| PsbHLH intF | Forward | GTGGAGGTTGGCTACTATGC | Ps 281-399 | intran 2 | mapping and Caméor's BAC library screening |
| PsbHLH intR | Reverse | GAGGTTAATGTTTCATCTATTACACC | Ps 281-399 | intran 2 | mapping and Caméor's BAC library screening |
| PS_bHLH_5432_L | Forward | TCCAATCGAAGAACCTCTCG | BAC 112D23 | exon 6 | association studies in PI lines |
| PS_bHLH_6089_R | Reverse | GACAATCCAAATTTACACTCGACA | BAC 112D23 | inton 6 | association studies in PI lines |
| PsbHLHA F1 | Forward | GCTTCAAAACATGTTGCAGG | BAC 112D23 | exon 1 |  |
| PsbHLHAR1 | Reverse | AAGCGCTCTCTTAACTTCCAC | BAC 112D23 | exon 7 |  |
| PsbHLHAF2 | Forward | GATGAAGATGAGGAAGAGGATG | BAC 112D23 | exon 6 | used with R7 to get the 2 mutations |
| PsbHLHAR2 | Reverse | TGAAAGATGATTGGGTGGAG | BAC 112D23 | exon 7 |  |
| PsbHLHAF3 | Forward | CTGTCTGGTAGACCAACCAGTC | BAC 112D23 | 5'UTR |  |
| PsbHLHAR3 | Reverse | CTCATTTGCTCCCGTGAGC | BAC 112D23 | exon 3 |  |
| PsbHLHAF4 | Forward | TGTGTGTACCTAGCTAGGTGATGAG | BAC 112D23 | intron 2 |  |
| PsbHLHAF5 | Forward | GGAAGGGAATGGCACTGG | BAC 112D23 | exon 7 |  |
| PsbHLHAR5 | Reverse | TCACAATAGCTACCTAGATCCTCC | BAC 112D23 | intron 2 |  |
| PsbHLHAF6 | Forward | TGTTGGTGAAGTATTTAGGTGCAC | BAC 112D23 | intran 2 |  |
| PsbHLHAR6 | Reverse | TCCAACACAGGAATGCATACC | BAC 112D23 | exon 5 |  |
| PsbHLHAF7 | Forward | CCATGGCTTCTCAATGATGAC | BAC 112D23 | intran 3 |  |
| PsbHLHAR7 | Reverse | GATGGACCACATTGATATGTGG | BAC 112D23 | intran 6 | used with F2 to get the 2 mutations |
| PsbHLHAF8 | Reverse | GACAATGGCAGGTGCTAACC | BAC 112D23 | intron 6 |  |
| PsbHLHAR8 | Reverse | TCTTCTTGGTTAAGTAACCAACCC | BAC 112D23 | intran 6 |  |
| PsbHLHA R8rc | Forward | GGGTTGGTTACTTAACCAAGAAGA | BAC 112D23 | intron 6 |  |
| PsbHLHAF9 | Forward | CATGTGCTGCAGCATAGGG | BAC 112D23 | intron 6 |  |
| PsbHLHA F10 | Forward | GGGTGTTAAATCACATGAGTGG | BAC 112D23 | intran 6 |  |
| PsbHLHA R10 | Reverse | CATATTCATCAAAACCCTCAAGG | BAC 112D23 | intran 6 |  |
| PsbHLHAF11 | Forward | ACATGCCTGGAATTGGC | BAC 112D23 | intron 6 |  |
| PsbHLHAR11 | Reverse | ACTTTCCTTGTTCTCTAAGGCC | BAC 112D23 | intran 6 |  |
| PsbHLHA F12 | Forward | GAGACCTCCGATATGATTGG | BAC 112D23 | intran 6 |  |
| PsbHLHA R12 | Reverse | GGAGAGGTTTCGTCGTGG | BAC 112D23 | exon 7 |  |
| PsbHLHA F13 | Forward | ATCTGACAAGAGAGGGGAAGG | BAC112D23 and JI399 | intron 6 |  |
| PsbHLHA R13 | Reverse | CATGTGTGTTGGTTTTCCATC | Ps alignment JI15-281-399-2822 intran 6 | |  |
| PsbHLHAF14 | Forward | TTGTTGTTGGAGACACTAGAGTAGC | BAC 112D23 and 2822-281 | intran 6 |  |
| PsbHLHA R14 | Reverse | CACGGCTTCTTGTGAAAGG | BAC112D23 and JI2822 | intran 6 |  |
| PsbHLHA R15 | Reverse | AATACCATACTTCTGTAAGAAGCCTC | BAC112D23 and 2822-281 | intran 6 |  |
| PsbHLHA F15 | Reverse | CTCAAACAGTAACCACCATTCTCC | BAC112D23 | exon 7 |  |
| Ps-A-ex06 F | Forward | GGAACCGAGTGAACTCATGC | BAC112D23 | exon 6 | used with int6 R to get the G to A transition |
| Ps-A-int6 R | Reverse | GCTTCAGTTTGTGAGTCCACC | BAC112D23 | intron 6 | used with ex06 F to get the G to A transition |
| KL244F | Forward | TTCTCTACAAAGAAGCCAGCAACTAA | PsbHLHA | exon 2 | PsbHLHA gene, used for qPCR |
| KL246R | Reverse | CCGACACCAGGAGGAAATGAGAAAG | PsbHLHA | exon 2 | bHLHA gene, used for qPCR |
| PsEF1F | Forward | TGTGCCAGTGGGACGTGTTG | PsEF1a |  | used as a reference gene in qPCR |
| PsEF1R | Reverse | CTCGTGGTGCATCTCAACGG | PsEF1a |  | used as a reference gene in qPCR |
| KL255F | Forward | CCTCTCGATGATTCACTACAAATTCAAT | PsbHLHA | exon 6 | bHLHA gene, used for Endpoint PCR |
| KL256R | Reverse | TGTCTTCTTGTGTTAAGTCTTCTAATGGATG PsbHLHA | | exon 7 | bHLHA gene, used for Endpoint PCR |

(*B*) Primers used in the identification and characterization of the *WD40* gene from pea.

| **primer name** | **primer sequence** | **design** | **use** |
| --- | --- | --- | --- |
| Mt TTG1-F2: | CCTTCGATCACCCTTACCC | CR940305.16 from *Medicago truncatula*  based on the alignment with ArabidopsisTTG1= NM_180739.2 | target TTG1 in pea |
| Mt TTG1-R2: | CTCAAATCCTTCTTGTTCCAAGC | CR940305.16 from *Medicago truncatula*  based on the alignment with ArabidopsisTTG1= NM_180739.2 | target TTG1 in pea |
| PsTTG1-R4: | GGTCGGAGGATGTTTTCTGG | JI2822 seq ( from Mt TTG1-F2/R2 PCR) | first set of SPCR on 5' |
| PsTTG1-R3: | ACGAACTTCCCAGAGACGG | JI2822 seq ( from Mt TTG1-F2/R2 PCR) | first set of SPCR on 5' |
| PsTTG1-F3: | GCTTCTGTTTCTGCTGATGGG | JI2822 seq ( from Mt TTG1-F2/R2 PCR) | first set of SPCR on 3' |
| PsTTG1-F4: | CAATTATCTATGAGAGTCCTCAACCAG | JI2822 seq ( from Mt TTG1-F2/R2 PCR) | first set of SPCR on 3' |
| PsTTG1-F6: | TGTATGGAACCAGTCCAAGTCC | JI2822seq ( from sequence obtain from the first set of SPCR on 5') | to amplify the promoter |
| PsTTG1-F5: | CCTCCTCTTCTTCCCACACC | JI2822seq ( from sequence obtain from the first set of SPCR on 5') | to amplify the 5' of the gene |
| Mt-TTG1-R5 | CAATCCAATCAGGCTGAGC | EU040206.1= *Medicago truncatula* cDNA for WD40-1 ( based on the aligment with lotus LjTTG1 mRNA=AB490777.1 | to get the 3' of the gene as the S-PCR didn't work |
| Ps TTG1-F7 | GGATCTTTGATCTAAGAGACAAAGAGC | JI2822 seq ( from Mt TTG1-F2/R2 PCR) | to use with F3 or F4 to try to get the SPCR on 3' more specific |
| Ps TTG1 F8: | CACTATGTCGGTCGAACCC | JI2822 seq ( from sequence obtain from the first set of SPCR on 5') | to amplify more of the promoter |
| Ps TTG1-F9 | GTGGCTGGTCCAAATGG | JI2822 seq ( from sequence obtain from the second set of SPCR 3') | to amplify the 3' of the gene and what follows |
| Ps TTG1-R6: | CACCAAAAGAAGCCATTGC | JI2822 seq ( from sequence obtain from the second set of SPCR 3') | to amplify the 3' of the gene and what follows |
| Ps TTG1-R7: | GTGGCATGTAGGACCATACCA | JI2822 seq ( from sequence obtain from the second set of SPCR 3') | to amplify the 3' of the gene and what follows |
